# How much is enough? Exploring the dose-response relationship between cash transfers and surgical utilization in a resource-poor setting

**DOI:** 10.1371/journal.pone.0232761

**Published:** 2020-05-14

**Authors:** Christopher Strader, Joanna Ashby, Dominique Vervoort, Aref Ebrahimi, Shoghi Agbortoko, Melissa Lee, Naomi Reiner, Molly Zeme, Mark G. Shrime

**Affiliations:** 1 Program in Global Surgery and Social Change, Harvard Medical School, Boston, MA, United States of America; 2 Department of General Surgery, University of Massachusetts, Worcester, MA, United States of America; 3 School of Medicine, University of Glasgow, Scotland, United Kingdom; 4 Johns Hopkins Bloomberg School of Public Health, Baltimore, Maryland, United States of America; 5 Joslin Diabetes Center, Harvard Medical School, Boston, MA, United States of America; 6 Harvard College, Cambridge, MA, United States of America; 7 Harvard Graduate School of Education, Cambridge, MA, United States of America; 8 Center for Global Surgery Evaluation, Massachusetts Eye and Ear Infirmary, Boston, MA, United States of America; 9 Center for Health and Wellbeing, Princeton University, Princeton, NJ, United States of America; University of South Florida, UNITED STATES

## Abstract

**Objective:**

Cash transfers are a common intervention to incentivize salutary behavior in resource-constrained settings. Many cash transfer studies do not, however, account for the effect of the size of the cash transfer in design or analysis. A randomized, controlled trial of a cash-transfer intervention is planned to incentivize appropriate surgical utilization in Guinea. The aim of the current study is to determine the size of that cash transfer so as to maximize compliance while minimizing cost.

**Methods:**

Data were collected from nine coastal Guinean hospitals on their surgical capabilities and the cost of receiving surgery. These data were combined with publicly available data about the general Guinean population to create an agent-based model predicting surgical utilization. The model was validated to the available literature on surgical utilization. Cash transfer sizes from 0 to 1,000,000 Guinean francs were evaluated, with surgical compliance as the primary outcome.

**Results:**

Compliance with scheduled surgery increases as the size of a cash transfer increases. This increase is asymptotic, with a leveling in utilization occurring when the cash transfer pays for all the costs associated with surgical care. Below that cash transfer size, no other optima are found. Once a cash transfer completely covers the costs of surgery, other barriers to care such as distance and hospital quality dominate

**Conclusion:**

Cash transfers to incentivize health-promoting behavior appear to be dose-dependent. Maximal impact is likely only to occur when full patient costs are eliminated. These findings should be incorporated in the design of future cash transfer studies.

## Introduction

One-third of the world’s disease burden requires surgical treatment,[1] but five billion people lack access to safe, timely, and affordable surgical, obstetric, and anesthesic care.[2] Barriers to surgical care faced by patients are myriad, though cost is often among the most significant.[3]

Costs of surgery cause important downstream side effects. Up to 81 million surgical patients are driven into financial catastrophe each year as a result of accessing surgical care.[4,5] For only 40% (or 33 million) of these patients, however, the catastrophic expense can be attributed to the direct medical costs of care, such as doctor fees, surgical fees, medicines, supplies, laboratory tests, and imaging. For the majority—48 million people—financial catastrophe comes from non-medical costs of care, such as the cost of transport, food, and lodging.[5]

Since the publication of the *Lancet Commission on Global Surgery*, significant attention has been paid to improving access to surgery in low- and middle-income countries (LMICs). Much of this attention has focused on addressing the supply-side barriers to surgical access, including surgeon training,[6,7] task-shifting,[8,9] and quality improvement initiatives.[10,11] Little attention has been placed on the demand-side barriers such as affordability, despite their high prevalence in surveys of patients without surgical access.

Addressing demand-side barriers can be difficult.[12,13] Simply offering free surgery is insufficient: patients often continue to report cost barriers, even with free surgery.[14] A recent retrospective study of surgery at a charitable institution in three sub-Saharan African countries, where the majority of the population lives in rural settings, demonstrated that non-medical costs and a lack of transportation were a major barrier to care access. Controlling for confounders, patients seeking care at this institution, which provides surgery itself for free, had a no-show rate of 28% if transportation was not paid for. When transportation was provided, the no-show rate dropped to 15%.[14]

Cash transfers—in which participants are given a small bolus of cash, either unconditionally or conditional on their adherence to a behavior—have been used to incentivize salutary action around nutrition,[15,16] maternal care,[17,18] HIV prevention and care,[19,20] and education.[21] These studies have shown mixed effects, not all of which have been positive.[22] For example, a large, phase 3 randomized, controlled trial of a cash transfer in rural South Africa found no effect of the cash transfer on HIV incidence among its 2,500 participants.[20]

Unfortunately, interpreting these heterogenous results is difficult because many cash transfer studies do not report the methods by which the size of the cash transfer itself is chosen.[16,20] In other cases, a cash transfer dose is linked to a proportion of household income, without explicit methodological justification for the choice of proportion.[17] This method of dosing is inconsistent with dose design for other interventions such as drug development.

To our knowledge, no cash transfer trial has been introduced to increase access to care for patients requiring emergency or essential surgical care besides C-sections. Such a trial for surgical patients is planned in Guinea. The analysis in the current paper attempts to determine the optimal dose of such a cash transfer, in preparation for the planned trial. Our hypothesis is that an optimal cash transfer dose exists, one which can maximize adherence to planned surgical care but is smaller than maximal size. In other words, if an optimal dose exists, we hypothesize that it would be less than paying for the entirety of a patient’s medical and non-medical costs. We test this hypothesis through the use of an agent-based model of surgical delivery in coastal Guinea.

## Methods

### Model design

Agent-based modeling is a stochastic modeling framework in which individual actors—in this case, patients and hospitals—respond to their own internal stochastic rules. The stochasticity of these models allows the modeler to incorporate patient-level heterogeneity and parameter uncertainty, both of which are important to develop adequately optimized estimates for the size of a cash transfer for surgical patients.

The model description below follows the Overview, Design, and Details (ODD) protocol.[23] The purpose of this model was to determine the dose-response curve for cash transfers for surgical patients in Guinea and to discover an optimal cash transfer, if any. The model had two entities: hospitals and people. Hospitals were constructed with the following state variables: latitude, longitude, surgical cost, and quality score. The person entity contained the following state variables: latitude, longitude, wealth quintile, educational level, gender, age, income, and preference weights calculated based on a utilization function that we have used in previous agent-based models.[24] The model scaled to a 60km x 60km square, centered on Conakry, Guinea, encompassing the costal Guinean hospitals felt by the Ministry of Health to be able to perform surgery.

The model proceeded according to the following process and schedule: For each tested dose of the cash transfer, the individual agents faced the choice of whether and where to seek surgical treatment for their condition, or to forgo treatment, based on a utilization function that we have used in previous agent-based models.[24] The decision to seek care was based on the individual and provider factors listed above. The cash transfer was modeled as a decrease in the perceived cost of care within the choice function detailed below. Cash-transfer sizes of 0 to 1,000,000 Guinean francs (GNF) were tested (1 USD is approximately 9,000 GNF), in increments of 10,000 GNF. Income was modeled as a quantized income distribution, as we have previously done.[24] For each agent, preference weights were drawn stochastically from a normal distribution with mean and standard deviation as in prior models.[24] From this, a value score was calculated for care at each hospital and for declining to seek care, following the choice function below. Agents then chose to seek care at a hospital or to remain home probabilistically, following the logit function below.

With respect to the model’s design, the basic principle at play within the model is McFadden’s random utility theory[25], which postulates that utility can be decomposed into an observable, deterministic function (solved for above), and an unobservable, stochastic error term, which is incorporated into the final probabilistic decision made by each individual in the model.

Specifically, patient choice for care was modeled using the following choice function, in which *i* represents an individual and *j* a provider hospital. The *X* vector represents agent-specific characteristics (age, gender, wealth, education, etc). *Q* is a quality score incorporating the hospital-specific data, standardized from 0 to 1 against the best performing hospital in each domain. *D* represents distance, via the road networks in Guinea. And finally, *c*(·) is a cost function which includes an agent’s income, *Y*, and the price of care, *P*, the latter also derived from the previously-collected data. The variable *t* represents the cash transfer.

$$U_{ij}=\alpha +\beta X_{i}+\gamma Q_{j}+\delta D_{ij}+\lambda c(Y_{i}-P_{j}+t)+\varepsilon$$

An individual, *i*, chooses care at hospital *j* if their utility for care at that hospital, *U*_*ij*_, is higher than for care at other locations. However, because utility is not directly observable, the choice is probabilistic, with the probability of choosing care at hospital *k*, *P*_*ik*_, equal to:

$$P_{ik}=\frac{\exp\left[\alpha +\beta X_{i}+\gamma Q_{k}+\delta D_{ik}+\lambda c(Y_{i}-P_{k}+t)\right]}{\sum_{j\in J}\exp\left[\alpha +\beta X_{i}+\gamma Q_{j}+\delta D_{ij}+\lambda c(Y_{i}-P_{j}+t)\right]}$$

Care at home (*ie*, forgoing formal care) is included within the set *J*. For care at home, *Q*, *D*, and *P* are set to zero.

Through the use of agent-based modelling, we expect the emergence of the complex interplay of the various barriers to care faced by individuals, only one of which—cost—is alleviated by a cash transfer. Individuals adapt to their environment through the choice of care; the logit model makes individuals more likely (but not guaranteed) to choose the care provider that maximizes their utility, which is their objective function. Because it tracks a single decision, no learning or prediction is incorporated into the model. Individuals know their own age, gender, wealth, income, and education, and sense the perceived quality of each hospital.

Individuals interact with hospitals but not with each other. Although prior models have evaluated the effect of interventions on families, no collectives are formed in this model. Stochasticity has been discussed above. The primary observation is healthcare utilization. At initialization, 100,000 person agents are created to mimic the population of guinea. The nine hospitals are placed at their corresponding GPS coordinates.

### Input data

While patient-level demographics could be collected from publicly available datasets, including the Guinea DHS[26] and the WorldPop project,[27,28] public data for hospital quality was not available. Primary data was collected on the following information from the nine coastal Guinean hospitals that the Ministry of Health considered surgically capable: latitude, longitude, number of doctors, number of surgeons, number of anesthesiologists, number of surgical officers, number of anesthetic officers, number of nurses, number of operating rooms (ORs), total cases done in the last month, total general surgery cases in the last month, total obstetric cases in the last month, total otolaryngology/head and neck cases in the last month, and costs in GNF for laparotomy, cesarean section, goiter removal, and mandibulectomy. This data collection was performed in under the direction of and in conjunction with the Ministry of Health. Missing data was imputed using averages for each domain.

### Data analysis and validation

The model was validated against the known population density in Guinea, against demographic parameters for the Guinean population, and against previously-published no-show rates.[14]

The model was constructed using the AnyLogic modeling platform (v.8.1.0, St. Petersburg). Data analysis was performed in R (v.3.4.0). The simulation was run 10,000 times, using batches of 20,000 population per run, with simulation estimates reported as mean (95% uncertainty interval).

## Results

### Agent demographics

**Fig 1:**
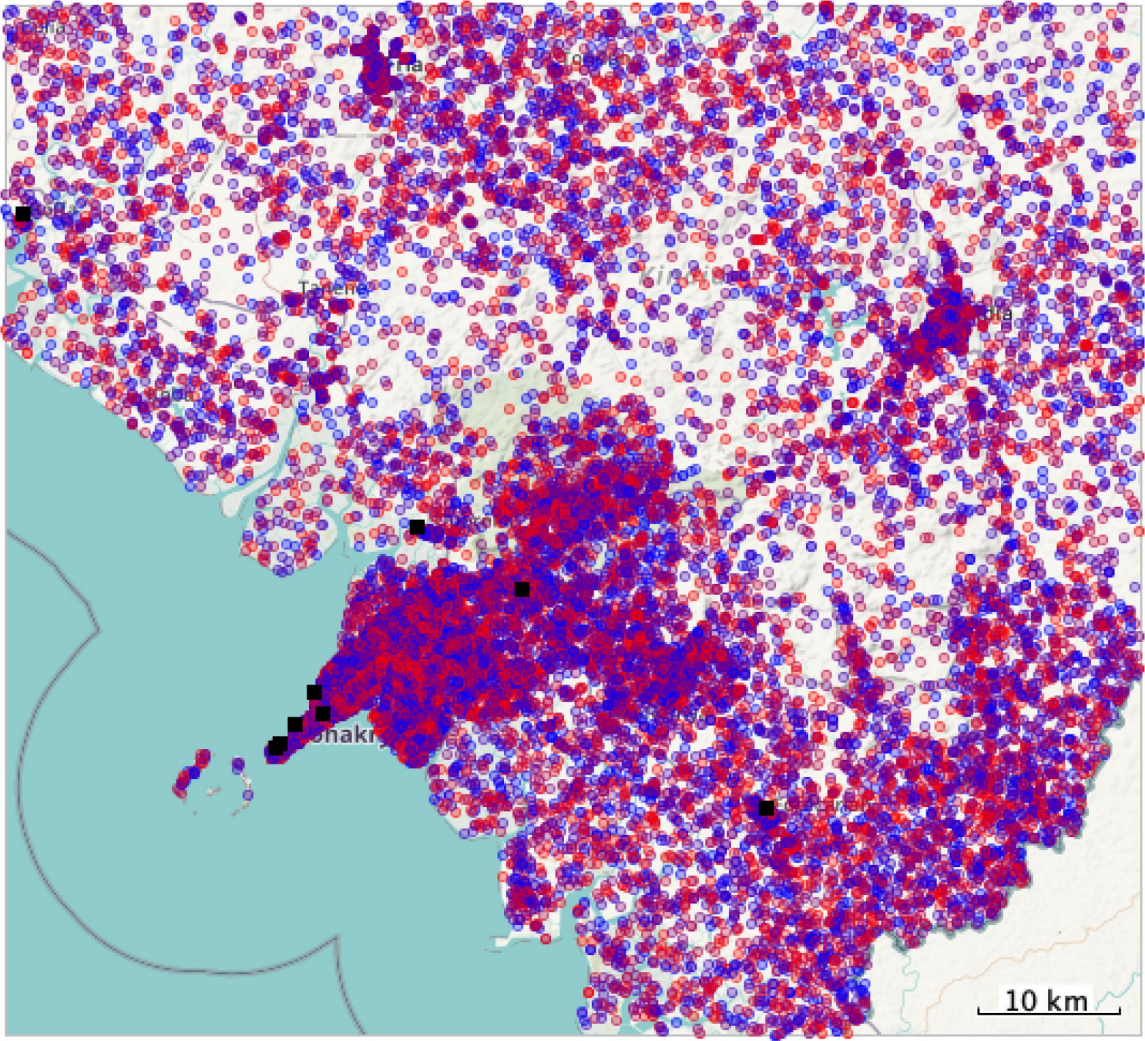
Population and hospital placement in the agent-based model. Hospitals are represented by black squares. The population is represented with circles shaded blue (poorest) to red (richest). Conakry, the capital of Guinea, is located on the densely-populated peninsula toward the southwest in this figure.

Agent demographics are shown in **Table 1**, and their geographic location relative to the evaluated hospitals is in **Fig 1**. No significant difference exists between the modeled agents and the Guinean population.

**Table 1:** Demographic characteristics of the model agents.

Population Demographics		
**Total Population** (N)	100,000	
**Male** (mean (sd))	0.5	0.5
**Education** (N, %)		
None	53422	53.4
Primary	16642	16.6
Secondary	29936	29.9
**Wealth** (N, %)		
Poorest	20,131	20.1
Poor	19,959	20.0
Middle	19,937	19.9
Rich	20,052	20.1
Richest	19,921	19.9
**Income** (median (IQR))	GNF 554,986	GNF 341,936–842,990
**Age** (N, %)		
5–14	33,261	33.3
15–21	8,264	8.3
22–49	17,547	17.5
50+	40,928	40.9

Demographic characteristics of the model agents.

### Hospital characteristics

Hospital characteristics are shown in **Table 2**. Costs varied widely depending on the operation performed. The mean cost of surgery was 274,125 GNF (approximately 30 USD). The average cost of a caesarean section was 71,428 GNF (approximately 8 USD), because the majority of surveyed hospitals offered the procedure for free. For other procedures, cost varied from 250,000–600,000 GNF (mean: 431,778; median: 450,000), or 28–67 USD. World Bank estimates of monthly household expenditure for an average Guinean household were 457,785 GNF (51 USD) in 2018.[29]

**Table 2:** Hospital characteristics. Costs are given in Guinean francs (GNF). “—” signifies that the value was unknown by the responding hospital representative or the Ministry of Health. “NA” signifies that the hospital did not offer that specific type of operation.

**Hospital ID**	MDs	Surgeons	Surgical officers	Anesthesia MDs	Anesthetic officers	Nurses	ORs	Total cases last month	Total general surgery	Total OB	Total ENT / head and neck	Cost: laparotomy	Cost: CS	Cost: Goitre	Cost: mandibulectomy
**A**	250	100	3	1	27	350	10	800	100	400	20	450,000	0	450,000	450,000
**B**	200	70	0	5	10	102	10	—	—	—	—	486,000	0	—	—
**C**	5	2	0	—	—	—	1	—	—	—	—	—	—	—	—
**D**	17	8	0	5	3	12	0*	40	23	17	0	600,000	0	NA	250,000
**E**	15	—	—	—	—	—	1	—	—	—	—	—	—	—	—
**F**	23	2	0	0	1	12	2	—	—	—	—	400,000	0	NA	NA
**G**	2	1	0	0	1	4	1	282	4	0	0	—	500,000	NA	NA
**H**	23	2	0	0	1	12	—	—	—	—	—	400,000	0	NA	NA
**I**	12	4	5	0	1	15	2		35	22	0	400,000	0	NA	NA

*Although this hospital reported 40 cases in the prior month, they also reported no formal operating rooms.

### Dose-response curve for cash transfers

**Fig 2:**
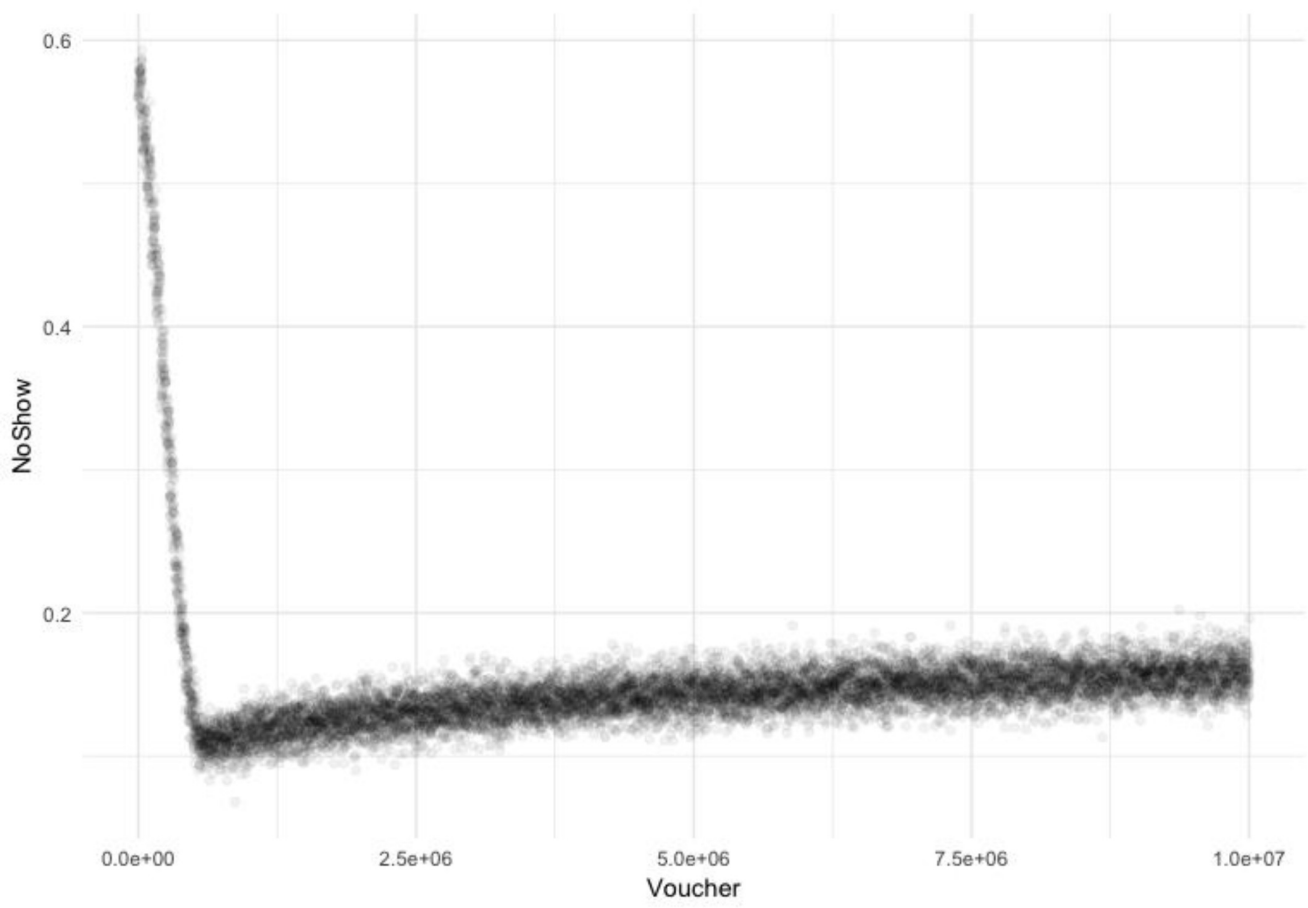
Predicted no-show rate by cash transfer size. Cash transfer given in Guinean francs (GNF). Each point represents a single model run with a single cash transfer (“voucher”) dose.

The dose-response curve for the effect of cash transfers on surgical utilization is shown in **Fig 2**. Without any cash transfer—that is, the agent would face the full brunt of the cost of surgery—the no-show rate for surgery is expected to be 56.6% (95% UI: 55.6%– 57.5%). At cash transfer doses equal to the average cost of surgery, the no-show rate mirrors the expected, published no-show rate of 28% for a non-governmental organization (NGO) providing free surgery.[14] With a cash transfer of 280,000, the predicted no-show rate is 30.0% (95% UI: 24.5%– 35.4%).

Fig 2 shows that there is an optimal cash transfer dose. Beginning at a cash transfer size of 600,000 GNF, the no-show rate flattens, then begins to rise again slowly. It should be noted that 600,000 GNF (67 USD) is also the highest cost for surgery in any of the hospitals.

### Heterogeneity

We performed a subgroup analysis for the effect of the cash transfer. Although small differences were seen in the effect of the cash transfer size on no-show rates between gender, education, and age groups, none of these differences were statistically significant.

## Discussion

In this paper, we explore the dose-response relationship between cash transfers and surgical utilization through an agent-based model. We show, in a model validated to the status quo of surgical utilization, that the only optimal cash transfer dose is “pay for everything”. That is, until full costs are covered, any incremental increase in the dose of a cash transfer is expected to improve compliance. However, once full costs are covered, further increases do not appear to incentivize more salutary behavior. In those cases, other barriers to care—such as distance, education, and hospital quality—dominate. A slow increase in the no-show rate above the asymptote is observed, especially as the cash transfer size overshadows the income of the agents.

In 2015, the World Health Assembly (WHA) adopted Resolution WHA68.15, which declared surgery, obstetric, and anesthesia care a key “component of universal health coverage”.[30] Despite this resolution, and despite the fact that financial protection is one of the three pillars of universal health coverage, financial burden remains a primary obstacle to surgical access.[2–4,4,14]

Cash transfers have been shown previously to increase compliance in health, nutrition, and educational domains, but are rare in surgery. A randomized controlled trial has been planned for cash transfers in surgery, and this study was performed to find the correct dose of the cash transfer.

Cash transfers are common instruments in LMICs.[29–48] Over the last 10 years, scientific publications have evaluated cash transfer programs in 31 countries. The studies looked at the impact of cash transfers on maternal health,[29,30] maternal mental health,[31] neonatal and infant mortality,[32,33] childhood malnutrition,[34,35] childhood vaccinations,[36,37] childhood illness,[38] childhood and adolescent education,[39,40] sexual and reproductive health practices of sex workers, mothers and adolescents,[41,42] geriatric health,[43] HIV incidence[44] and prevalence of high risk behavior for HIV,[45] and incidence of TB.[46]

Only one study evaluated the impact of cash transfers on the uptake of surgical services, specifically C-sections in Mexico.[47] A second study looked at the ability of a $10 conditional cash transfer to increase on uptake of voluntary male medical circumcision counselling.[48]

Few studies have evaluated the dose-response of a cash transfer. In the male circumcision counseling study, for example, no explanation is given for the $10 dose of the cash transfer, and the response is interpreted in comparison with other LMIC studies using approximately equivalent transfer doses. This study aimed to rectify this gap in knowledge. We aimed to identify any optima in the dose-response curve for cash transfers. Our results suggest that the only optimum is to maximize the cash transfer so as to cover all patient costs—both medical and non-medical. It also suggests that any further incentive is likely to be overpowered by other barriers to care facing patients.

### Limitations and strengths

Like all studies, this one has limitations. Although this study uses a modeling technique, we have used the technique multiple times previously to evaluate successfully the effects of policies on utilization and patient behavior.[23,49] However, although our current model proved calibrated to published patient behavior, all models should be viewed as hypothesis-generating rather than hypothesis-testing. Secondly, the outcome of interest was binary—show vs. no-show. Despite this being the outcome most likely to be driven by a cash transfer, it only partially describes the downstream impacts of salutary health behavior.

Third, Guinea, like all LMICs, cannot be seen as an isolated economic system. Regional differences in infrastructure and uptake are likely to exist. Many NGOs, development assistance for health bodies, and parallel markets influence patients’ decisions to seek care in specific places (e.g., outside Conakry) or to wait for other opportunities (e.g., nearby NGO care). This model does not include these externalities because it takes a single-decision time horizon. In addition, individuals within the model are assigned wealth and income at random, weighted to reflect the country-wide distribution of wealth and income. This most certainly does not reflect reality: wealth and income correlates with other important factors such as location. Unfortunately, Guiena does not produce publicly available data on the geographic dispersion of these sociodemographic indicators. Finally, patient choice can never fully be explained by a mathematical choice function. Although our model describes the status quo well, caution should be taken against overinterpreting the results.

Nevertheless, this study has several strengths. It is the first to propose a continuous dose-response curve for cash transfers and to demonstrate the asymptotic relationship of dose with behavior. It is also the first to evaluate cash transfers in a surgical context. Finally, the methodology is simple, allowing replication in other countries.

## Conclusion

We demonstrate that cash transfers are likely to improve access to surgical care, and are likely to be dose-dependent, and that maximal impact only occurs when full patient costs are eliminated. Our results suggest that, prior to the implementation of a cash transfer experiment *in vivo*, consideration should be taken to design the dose of this transfer with this dose-response curve in mind.

## Supporting information

S1 File (RTF)
