# Supplementary material for: How much is enough? Exploring the dose-response relationship between cash transfers and surgical utilization in a resource-poor setting
Source: PLoS One. 2020 May 14;15(5):e0232761. doi: 10.1371/journal.pone.0232761 (PMC7224483; doi:10.1371/journal.pone.0232761)
Supplement: S1 File — (RTF) [file pone.0232761.s001.rtf]

package malawi_recentralization_model_2017_06_04;import java.io.Serializable;import java.sql.Connection;import java.sql.SQLException;import java.util.ArrayDeque;import java.util.ArrayList;import java.util.Arrays;import java.util.Calendar;import java.util.Collection;import java.util.Collections;import java.util.Comparator;import java.util.Currency;import java.util.Date;import java.util.Enumeration;import java.util.HashMap;import java.util.HashSet;import java.util.Hashtable;import java.util.Iterator;import java.util.LinkedHashMap;import java.util.LinkedHashSet;import java.util.LinkedList;import java.util.List;import java.util.ListIterator;import java.util.Locale;import java.util.Map;import java.util.PriorityQueue;import java.util.Random;import java.util.Set;import java.util.SortedMap;import java.util.SortedSet;import java.util.Stack;import java.util.Timer;import java.util.TreeMap;import java.util.TreeSet;import java.util.Vector;import java.awt.Color;import java.awt.Font;import java.awt.Graphics2D;import java.awt.geom.AffineTransform;import com.anylogic.engine.connectivity.ResultSet;import com.anylogic.engine.connectivity.Statement;import com.anylogic.engine.elements.*;import com.anylogic.engine.markup.Network;import com.anylogic.engine.Position;import com.anylogic.engine.markup.PedFlowStatistics;import com.anylogic.engine.markup.DensityMap;import static java.lang.Math.*;import static com.anylogic.engine.UtilitiesArray.*;import static com.anylogic.engine.UtilitiesCollection.*;import static com.anylogic.engine.presentation.UtilitiesColor.*;import static com.anylogic.engine.HyperArray.*;import com.anylogic.engine.*;import com.anylogic.engine.analysis.*;import com.anylogic.engine.connectivity.*;import com.anylogic.engine.database.*;import com.anylogic.engine.gis.*;import com.anylogic.engine.markup.*;import com.anylogic.engine.presentation.*;import com.mysema.query.Tuple;import com.mysema.query.sql.SQLBindings;import static malawi_recentralization_model_2017_06_04.DBDescriptor.*;import javax.swing.JApplet;public class ParamVar extends ExperimentParamVariation<Main> {  @AnyLogicInternalCodegenAPI  public static String[] COMMAND_LINE_ARGUMENTS_xjal = new String[0];  {    setCommandLineArguments_xjal( COMMAND_LINE_ARGUMENTS_xjal );  }  // Excel Files  public ExcelFile paramVar = new ExcelFile( ParamVar.this, "/malawi_recentralization_model_2017_06_04/", "/Users/Mark/Dropbox/@Papers/@Runyon/Anylogic Model/Model output/ParamVar.xlsx", false );  @AnyLogicInternalCodegenAPI  private static Map<String, IElementDescriptor> elementDesciptors_xjal = createElementDescriptors( ParamVar.class );    @AnyLogicInternalCodegenAPI  @Override  public Map<String, IElementDescriptor> getElementDesciptors() {    return elementDesciptors_xjal;  }  // View areas  @Override  @AnyLogicInternalCodegenAPI  public void drawModelElements(Panel _panel, Graphics2D _g, boolean _publicOnly, boolean _isSuperClass ) {    if (!_publicOnly) {      drawExcelFile( _panel, _g, -210, 20, 10, 0, "paramVar", paramVar );    }}  @Override  @AnyLogicInternalCodegenAPI  public boolean onClickModelAt( Panel _panel, double _x, double _y, int _clickCount, boolean _publicOnly, boolean _isSuperClass ) {    if( !_publicOnly && modelElementContains(_x, _y, -210, 20) ){      _panel.addInspect( -210, 20, this, "paramVar" );       return true;    }    return false;  }  @AnyLogicInternalCodegenAPI  protected static final Font _button_Font = new Font("Dialog", 0, 11 );  @AnyLogicInternalCodegenAPI  protected static final Font _text_Font = new Font("SansSerif", 0, 24 );  @AnyLogicInternalCodegenAPI  protected static final Font _text1_Font = new Font("SansSerif", 0, 12 );  @AnyLogicInternalCodegenAPI  protected static final Font _text2_Font = _text1_Font;  @AnyLogicInternalCodegenAPI  protected static final Font _text3_Font = _text1_Font;  @AnyLogicInternalCodegenAPI  protected static final Font _text4_Font = _text1_Font;  @AnyLogicInternalCodegenAPI  protected static final Font _text5_Font = new Font("SansSerif", 1, 12 );  @AnyLogicInternalCodegenAPI  protected static final Font _text6_Font = _text1_Font;  @AnyLogicInternalCodegenAPI  protected static final Font _text7_Font = _text1_Font;  @AnyLogicInternalCodegenAPI  protected static final Font _text8_Font = _text1_Font;  @AnyLogicInternalCodegenAPI  protected static final Font _text9_Font = _text1_Font;  @AnyLogicInternalCodegenAPI  protected static final Font _text10_Font = _text1_Font;  @AnyLogicInternalCodegenAPI  protected static final Font _text11_Font = _text1_Font;  @AnyLogicInternalCodegenAPI  protected static final Font _text12_Font = _text1_Font;  @AnyLogicInternalCodegenAPI  protected static final Font _text13_Font = _text1_Font;  @AnyLogicInternalCodegenAPI  protected static final Font _text14_Font = _text1_Font;  @AnyLogicInternalCodegenAPI  protected static final Font _text15_Font = _text1_Font;  @AnyLogicInternalCodegenAPI  protected static final Font _text16_Font = _text1_Font;  @AnyLogicInternalCodegenAPI  protected static final Font _text17_Font = _text1_Font;  @AnyLogicInternalCodegenAPI  protected static final Font _text18_Font = _text1_Font;  @AnyLogicInternalCodegenAPI  protected static final Font _text19_Font = _text1_Font;  @AnyLogicInternalCodegenAPI  protected static final Font _text20_Font = _text1_Font;  @AnyLogicInternalCodegenAPI  protected static final Font _text21_Font = _text1_Font;  @AnyLogicInternalCodegenAPI  protected static final Font _text22_Font = _text1_Font;  @AnyLogicInternalCodegenAPI  protected static final Font _text23_Font = _text1_Font;  @AnyLogicInternalCodegenAPI  protected static final Font _text24_Font = _text1_Font;  @AnyLogicInternalCodegenAPI  protected static final Font _text25_Font = _text1_Font;  @AnyLogicInternalCodegenAPI  protected static final Font _text26_Font = _text1_Font;  @AnyLogicInternalCodegenAPI  protected static final Font _text27_Font = _text1_Font;  @AnyLogicInternalCodegenAPI  protected static final Font _text28_Font = _text1_Font;  @AnyLogicInternalCodegenAPI  protected static final Font _text29_Font = _text1_Font;  @AnyLogicInternalCodegenAPI  protected static final Font _text30_Font = _text1_Font;  @AnyLogicInternalCodegenAPI  protected static final Font _text31_Font = _text1_Font;  @AnyLogicInternalCodegenAPI  protected static final Font _text32_Font = _text1_Font;  @AnyLogicInternalCodegenAPI  protected static final Font _text33_Font = _text1_Font;  @AnyLogicInternalCodegenAPI  protected static final Font _text34_Font = _text1_Font;  @AnyLogicInternalCodegenAPI  protected static final Font _text35_Font = _text1_Font;  @AnyLogicInternalCodegenAPI  protected static final Font _text36_Font = _text1_Font;  @AnyLogicInternalCodegenAPI  protected static final Font _text37_Font = _text1_Font;  @AnyLogicInternalCodegenAPI  protected static final Font _text38_Font = _text1_Font;  @AnyLogicInternalCodegenAPI  protected static final Font _text39_Font = _text1_Font;  @AnyLogicInternalCodegenAPI  protected static final Font _text40_Font = _text1_Font;  @AnyLogicInternalCodegenAPI  protected static final Font _text41_Font = _text1_Font;  @AnyLogicInternalCodegenAPI  protected static final Font _text42_Font = _text1_Font;  @AnyLogicInternalCodegenAPI  protected static final Font _text43_Font = _text1_Font;  @AnyLogicInternalCodegenAPI  protected static final Font _text44_Font = _text1_Font;  @AnyLogicInternalCodegenAPI  protected static final Font _text45_Font = _text1_Font;  @AnyLogicInternalCodegenAPI  protected static final Font _text46_Font = _text1_Font;  @AnyLogicInternalCodegenAPI  protected static final Font _text47_Font = _text1_Font;  @AnyLogicInternalCodegenAPI  protected static final Font _text48_Font = _text1_Font;  @AnyLogicInternalCodegenAPI  protected static final Font _text49_Font = _text1_Font;  @AnyLogicInternalCodegenAPI  protected static final Font _text50_Font = _text1_Font;  @AnyLogicInternalCodegenAPI  protected static final Font _text51_Font = _text1_Font;  @AnyLogicInternalCodegenAPI  protected static final Font _text52_Font = _text1_Font;  @AnyLogicInternalCodegenAPI  protected static final Font _text53_Font = _text1_Font;  @AnyLogicInternalCodegenAPI  protected static final Font _text54_Font = _text1_Font;  @AnyLogicInternalCodegenAPI  protected static final Font _text55_Font = _text1_Font;  @AnyLogicInternalCodegenAPI  protected static final Font _text56_Font = _text1_Font;  @AnyLogicInternalCodegenAPI  protected static final Font _text57_Font = _text1_Font;  @AnyLogicInternalCodegenAPI  protected static final Font _text58_Font = _text1_Font;  @AnyLogicInternalCodegenAPI  protected static final Font _text59_Font = _text1_Font;  @AnyLogicInternalCodegenAPI  protected static final Font _text60_Font = _text1_Font;  @AnyLogicInternalCodegenAPI  protected static final Font _text61_Font = _text1_Font;  @AnyLogicInternalCodegenAPI  protected static final Font _text62_Font = _text1_Font;  @AnyLogicInternalCodegenAPI  protected static final Font _text63_Font = _text1_Font;  @AnyLogicInternalCodegenAPI  protected static final Font _text64_Font = _text1_Font;  @AnyLogicInternalCodegenAPI  protected static final Font _text65_Font = _text1_Font;  @AnyLogicInternalCodegenAPI  protected static final Font _text66_Font = _text1_Font;  @AnyLogicInternalCodegenAPI  protected static final Font _text67_Font = _text1_Font;  @AnyLogicInternalCodegenAPI  protected static final Font _text68_Font = _text1_Font;  @AnyLogicInternalCodegenAPI  protected static final Font _text69_Font = _text1_Font;  @AnyLogicInternalCodegenAPI  protected static final int _button = 1;  @AnyLogicInternalCodegenAPI  protected static final int _text = 2;  @AnyLogicInternalCodegenAPI  protected static final int _text1 = 3;  @AnyLogicInternalCodegenAPI  protected static final int _text2 = 4;  @AnyLogicInternalCodegenAPI  protected static final int _text3 = 5;  @AnyLogicInternalCodegenAPI  protected static final int _text4 = 6;  @AnyLogicInternalCodegenAPI  protected static final int _line = 7;  @AnyLogicInternalCodegenAPI  protected static final int _text5 = 8;  @AnyLogicInternalCodegenAPI  protected static final int _text6 = 9;  @AnyLogicInternalCodegenAPI  protected static final int _text7 = 10;  @AnyLogicInternalCodegenAPI  protected static final int _text8 = 11;  @AnyLogicInternalCodegenAPI  protected static final int _text9 = 12;  @AnyLogicInternalCodegenAPI  protected static final int _text10 = 13;  @AnyLogicInternalCodegenAPI  protected static final int _text11 = 14;  @AnyLogicInternalCodegenAPI  protected static final int _text12 = 15;  @AnyLogicInternalCodegenAPI  protected static final int _text13 = 16;  @AnyLogicInternalCodegenAPI  protected static final int _text14 = 17;  @AnyLogicInternalCodegenAPI  protected static final int _text15 = 18;  @AnyLogicInternalCodegenAPI  protected static final int _text16 = 19;  @AnyLogicInternalCodegenAPI  protected static final int _text17 = 20;  @AnyLogicInternalCodegenAPI  protected static final int _text18 = 21;  @AnyLogicInternalCodegenAPI  protected static final int _text19 = 22;  @AnyLogicInternalCodegenAPI  protected static final int _text20 = 23;  @AnyLogicInternalCodegenAPI  protected static final int _text21 = 24;  @AnyLogicInternalCodegenAPI  protected static final int _text22 = 25;  @AnyLogicInternalCodegenAPI  protected static final int _text23 = 26;  @AnyLogicInternalCodegenAPI  protected static final int _text24 = 27;  @AnyLogicInternalCodegenAPI  protected static final int _text25 = 28;  @AnyLogicInternalCodegenAPI  protected static final int _text26 = 29;  @AnyLogicInternalCodegenAPI  protected static final int _text27 = 30;  @AnyLogicInternalCodegenAPI  protected static final int _text28 = 31;  @AnyLogicInternalCodegenAPI  protected static final int _text29 = 32;  @AnyLogicInternalCodegenAPI  protected static final int _text30 = 33;  @AnyLogicInternalCodegenAPI  protected static final int _text31 = 34;  @AnyLogicInternalCodegenAPI  protected static final int _text32 = 35;  @AnyLogicInternalCodegenAPI  protected static final int _text33 = 36;  @AnyLogicInternalCodegenAPI  protected static final int _text34 = 37;  @AnyLogicInternalCodegenAPI  protected static final int _text35 = 38;  @AnyLogicInternalCodegenAPI  protected static final int _text36 = 39;  @AnyLogicInternalCodegenAPI  protected static final int _text37 = 40;  @AnyLogicInternalCodegenAPI  protected static final int _text38 = 41;  @AnyLogicInternalCodegenAPI  protected static final int _text39 = 42;  @AnyLogicInternalCodegenAPI  protected static final int _text40 = 43;  @AnyLogicInternalCodegenAPI  protected static final int _text41 = 44;  @AnyLogicInternalCodegenAPI  protected static final int _text42 = 45;  @AnyLogicInternalCodegenAPI  protected static final int _text43 = 46;  @AnyLogicInternalCodegenAPI  protected static final int _text44 = 47;  @AnyLogicInternalCodegenAPI  protected static final int _text45 = 48;  @AnyLogicInternalCodegenAPI  protected static final int _text46 = 49;  @AnyLogicInternalCodegenAPI  protected static final int _text47 = 50;  @AnyLogicInternalCodegenAPI  protected static final int _text48 = 51;  @AnyLogicInternalCodegenAPI  protected static final int _text49 = 52;  @AnyLogicInternalCodegenAPI  protected static final int _text50 = 53;  @AnyLogicInternalCodegenAPI  protected static final int _text51 = 54;  @AnyLogicInternalCodegenAPI  protected static final int _text52 = 55;  @AnyLogicInternalCodegenAPI  protected static final int _text53 = 56;  @AnyLogicInternalCodegenAPI  protected static final int _text54 = 57;  @AnyLogicInternalCodegenAPI  protected static final int _text55 = 58;  @AnyLogicInternalCodegenAPI  protected static final int _text56 = 59;  @AnyLogicInternalCodegenAPI  protected static final int _text57 = 60;  @AnyLogicInternalCodegenAPI  protected static final int _text58 = 61;  @AnyLogicInternalCodegenAPI  protected static final int _text59 = 62;  @AnyLogicInternalCodegenAPI  protected static final int _text60 = 63;  @AnyLogicInternalCodegenAPI  protected static final int _text61 = 64;  @AnyLogicInternalCodegenAPI  protected static final int _text62 = 65;  @AnyLogicInternalCodegenAPI  protected static final int _text63 = 66;  @AnyLogicInternalCodegenAPI  protected static final int _text64 = 67;  @AnyLogicInternalCodegenAPI  protected static final int _text65 = 68;  @AnyLogicInternalCodegenAPI  protected static final int _text66 = 69;  @AnyLogicInternalCodegenAPI  protected static final int _text67 = 70;  @AnyLogicInternalCodegenAPI  protected static final int _text68 = 71;  @AnyLogicInternalCodegenAPI  protected static final int _text69 = 72;  /** Internal constant, shouldn't be accessed by user */  @AnyLogicInternalCodegenAPI  protected static final int _SHAPE_NEXT_ID_xjal = 73;   /**   * Top-level presentation group id   */	   @AnyLogicInternalCodegenAPI  protected static final int _presentation = 0;  /**   * Top-level icon group id   */	   @AnyLogicInternalCodegenAPI  protected static final int _icon = -1;    @Override  @AnyLogicInternalCodegenAPI  public void executeShapeControlAction( int _shape, int index ) {    switch( _shape ) {      case _button: {          ShapeButton self = this.button;run(); ;}        break;      default:        super.executeShapeControlAction( _shape, index );        break;    }  }      /**   * <i>This method should not be called by user</i>   */  @AnyLogicInternalCodegenAPI  private void _button_SetDynamicParams_xjal( ShapeButton shape ) {    shape.setEnabled(getState() == IDLE );  }    protected ShapeButton button;  protected ShapeText text;  protected ShapeText text1;    /**   * <i>This method should not be called by user</i>   */  @AnyLogicInternalCodegenAPI  private void _text2_SetDynamicParams_xjal( ShapeText shape ) {    boolean _visible = getCurrentIteration() > 0 ;    shape.setVisible( _visible ); 	if ( _visible ) {    shape.setText(format(getCurrentIteration()) ); 	}  }    protected ShapeText text2;  protected ShapeText text3;    /**   * <i>This method should not be called by user</i>   */  @AnyLogicInternalCodegenAPI  private void _text4_SetDynamicParams_xjal( ShapeText shape ) {    boolean _visible = getCurrentIteration() > 0 ;    shape.setVisible( _visible ); 	if ( _visible ) {    shape.setText(format(getCurrentReplication()) ); 	}  }    protected ShapeText text4;  protected ShapeLine line;  protected ShapeText text5;  protected ShapeText text6;    /**   * <i>This method should not be called by user</i>   */  @AnyLogicInternalCodegenAPI  private void _text7_SetDynamicParams_xjal( ShapeText shape ) {    boolean _visible = getCurrentIteration() > 0 ;    shape.setVisible( _visible ); 	if ( _visible ) {    shape.setText(format( male ) ); 	}  }    protected ShapeText text7;  protected ShapeText text8;    /**   * <i>This method should not be called by user</i>   */  @AnyLogicInternalCodegenAPI  private void _text9_SetDynamicParams_xjal( ShapeText shape ) {    boolean _visible = getCurrentIteration() > 0 ;    shape.setVisible( _visible ); 	if ( _visible ) {    shape.setText(format( distance ) ); 	}  }    protected ShapeText text9;  protected ShapeText text10;    /**   * <i>This method should not be called by user</i>   */  @AnyLogicInternalCodegenAPI  private void _text11_SetDynamicParams_xjal( ShapeText shape ) {    boolean _visible = getCurrentIteration() > 0 ;    shape.setVisible( _visible ); 	if ( _visible ) {    shape.setText(format( educPrimaryComplete ) ); 	}  }    protected ShapeText text11;  protected ShapeText text12;    /**   * <i>This method should not be called by user</i>   */  @AnyLogicInternalCodegenAPI  private void _text13_SetDynamicParams_xjal( ShapeText shape ) {    boolean _visible = getCurrentIteration() > 0 ;    shape.setVisible( _visible ); 	if ( _visible ) {    shape.setText(format( educPrimary ) ); 	}  }    protected ShapeText text13;  protected ShapeText text14;    /**   * <i>This method should not be called by user</i>   */  @AnyLogicInternalCodegenAPI  private void _text15_SetDynamicParams_xjal( ShapeText shape ) {    boolean _visible = getCurrentIteration() > 0 ;    shape.setVisible( _visible ); 	if ( _visible ) {    shape.setText(format( age50 ) ); 	}  }    protected ShapeText text15;  protected ShapeText text16;    /**   * <i>This method should not be called by user</i>   */  @AnyLogicInternalCodegenAPI  private void _text17_SetDynamicParams_xjal( ShapeText shape ) {    boolean _visible = getCurrentIteration() > 0 ;    shape.setVisible( _visible ); 	if ( _visible ) {    shape.setText(format( age2249 ) ); 	}  }    protected ShapeText text17;  protected ShapeText text18;    /**   * <i>This method should not be called by user</i>   */  @AnyLogicInternalCodegenAPI  private void _text19_SetDynamicParams_xjal( ShapeText shape ) {    boolean _visible = getCurrentIteration() > 0 ;    shape.setVisible( _visible ); 	if ( _visible ) {    shape.setText(format( age1521 ) ); 	}  }    protected ShapeText text19;  protected ShapeText text20;    /**   * <i>This method should not be called by user</i>   */  @AnyLogicInternalCodegenAPI  private void _text21_SetDynamicParams_xjal( ShapeText shape ) {    boolean _visible = getCurrentIteration() > 0 ;    shape.setVisible( _visible ); 	if ( _visible ) {    shape.setText(format( age514 ) ); 	}  }    protected ShapeText text21;  protected ShapeText text22;    /**   * <i>This method should not be called by user</i>   */  @AnyLogicInternalCodegenAPI  private void _text23_SetDynamicParams_xjal( ShapeText shape ) {    boolean _visible = getCurrentIteration() > 0 ;    shape.setVisible( _visible ); 	if ( _visible ) {    shape.setText(format( q ) ); 	}  }    protected ShapeText text23;  protected ShapeText text24;    /**   * <i>This method should not be called by user</i>   */  @AnyLogicInternalCodegenAPI  private void _text25_SetDynamicParams_xjal( ShapeText shape ) {    boolean _visible = getCurrentIteration() > 0 ;    shape.setVisible( _visible ); 	if ( _visible ) {    shape.setText(format( intercept ) ); 	}  }    protected ShapeText text25;  protected ShapeText text26;    /**   * <i>This method should not be called by user</i>   */  @AnyLogicInternalCodegenAPI  private void _text27_SetDynamicParams_xjal( ShapeText shape ) {    boolean _visible = getCurrentIteration() > 0 ;    shape.setVisible( _visible ); 	if ( _visible ) {    shape.setText(format( educSecondaryComplete ) ); 	}  }    protected ShapeText text27;  protected ShapeText text28;    /**   * <i>This method should not be called by user</i>   */  @AnyLogicInternalCodegenAPI  private void _text29_SetDynamicParams_xjal( ShapeText shape ) {    boolean _visible = getCurrentIteration() > 0 ;    shape.setVisible( _visible ); 	if ( _visible ) {    shape.setText(format( educSecondary ) ); 	}  }    protected ShapeText text29;  protected ShapeText text30;    /**   * <i>This method should not be called by user</i>   */  @AnyLogicInternalCodegenAPI  private void _text31_SetDynamicParams_xjal( ShapeText shape ) {    boolean _visible = getCurrentIteration() > 0 ;    shape.setVisible( _visible ); 	if ( _visible ) {    shape.setText(format( logIncomeSq ) ); 	}  }    protected ShapeText text31;  protected ShapeText text32;    /**   * <i>This method should not be called by user</i>   */  @AnyLogicInternalCodegenAPI  private void _text33_SetDynamicParams_xjal( ShapeText shape ) {    boolean _visible = getCurrentIteration() > 0 ;    shape.setVisible( _visible ); 	if ( _visible ) {    shape.setText(format( logIncome ) ); 	}  }    protected ShapeText text33;  protected ShapeText text34;    /**   * <i>This method should not be called by user</i>   */  @AnyLogicInternalCodegenAPI  private void _text35_SetDynamicParams_xjal( ShapeText shape ) {    boolean _visible = getCurrentIteration() > 0 ;    shape.setVisible( _visible ); 	if ( _visible ) {    shape.setText(format( maleSE ) ); 	}  }    protected ShapeText text35;  protected ShapeText text36;    /**   * <i>This method should not be called by user</i>   */  @AnyLogicInternalCodegenAPI  private void _text37_SetDynamicParams_xjal( ShapeText shape ) {    boolean _visible = getCurrentIteration() > 0 ;    shape.setVisible( _visible ); 	if ( _visible ) {    shape.setText(format( distanceSE ) ); 	}  }    protected ShapeText text37;  protected ShapeText text38;    /**   * <i>This method should not be called by user</i>   */  @AnyLogicInternalCodegenAPI  private void _text39_SetDynamicParams_xjal( ShapeText shape ) {    boolean _visible = getCurrentIteration() > 0 ;    shape.setVisible( _visible ); 	if ( _visible ) {    shape.setText(format( educPrimaryCompleteSE ) ); 	}  }    protected ShapeText text39;  protected ShapeText text40;    /**   * <i>This method should not be called by user</i>   */  @AnyLogicInternalCodegenAPI  private void _text41_SetDynamicParams_xjal( ShapeText shape ) {    boolean _visible = getCurrentIteration() > 0 ;    shape.setVisible( _visible ); 	if ( _visible ) {    shape.setText(format( educPrimarySE ) ); 	}  }    protected ShapeText text41;  protected ShapeText text42;    /**   * <i>This method should not be called by user</i>   */  @AnyLogicInternalCodegenAPI  private void _text43_SetDynamicParams_xjal( ShapeText shape ) {    boolean _visible = getCurrentIteration() > 0 ;    shape.setVisible( _visible ); 	if ( _visible ) {    shape.setText(format( age50SE ) ); 	}  }    protected ShapeText text43;  protected ShapeText text44;    /**   * <i>This method should not be called by user</i>   */  @AnyLogicInternalCodegenAPI  private void _text45_SetDynamicParams_xjal( ShapeText shape ) {    boolean _visible = getCurrentIteration() > 0 ;    shape.setVisible( _visible ); 	if ( _visible ) {    shape.setText(format( age2249SE ) ); 	}  }    protected ShapeText text45;  protected ShapeText text46;    /**   * <i>This method should not be called by user</i>   */  @AnyLogicInternalCodegenAPI  private void _text47_SetDynamicParams_xjal( ShapeText shape ) {    boolean _visible = getCurrentIteration() > 0 ;    shape.setVisible( _visible ); 	if ( _visible ) {    shape.setText(format( age1521SE ) ); 	}  }    protected ShapeText text47;  protected ShapeText text48;    /**   * <i>This method should not be called by user</i>   */  @AnyLogicInternalCodegenAPI  private void _text49_SetDynamicParams_xjal( ShapeText shape ) {    boolean _visible = getCurrentIteration() > 0 ;    shape.setVisible( _visible ); 	if ( _visible ) {    shape.setText(format( age514SE ) ); 	}  }    protected ShapeText text49;  protected ShapeText text50;    /**   * <i>This method should not be called by user</i>   */  @AnyLogicInternalCodegenAPI  private void _text51_SetDynamicParams_xjal( ShapeText shape ) {    boolean _visible = getCurrentIteration() > 0 ;    shape.setVisible( _visible ); 	if ( _visible ) {    shape.setText(format( qSE ) ); 	}  }    protected ShapeText text51;  protected ShapeText text52;    /**   * <i>This method should not be called by user</i>   */  @AnyLogicInternalCodegenAPI  private void _text53_SetDynamicParams_xjal( ShapeText shape ) {    boolean _visible = getCurrentIteration() > 0 ;    shape.setVisible( _visible ); 	if ( _visible ) {    shape.setText(format( interceptSE ) ); 	}  }    protected ShapeText text53;  protected ShapeText text54;    /**   * <i>This method should not be called by user</i>   */  @AnyLogicInternalCodegenAPI  private void _text55_SetDynamicParams_xjal( ShapeText shape ) {    boolean _visible = getCurrentIteration() > 0 ;    shape.setVisible( _visible ); 	if ( _visible ) {    shape.setText(format( educSecondaryCompleteSE ) ); 	}  }    protected ShapeText text55;  protected ShapeText text56;    /**   * <i>This method should not be called by user</i>   */  @AnyLogicInternalCodegenAPI  private void _text57_SetDynamicParams_xjal( ShapeText shape ) {    boolean _visible = getCurrentIteration() > 0 ;    shape.setVisible( _visible ); 	if ( _visible ) {    shape.setText(format( educSecondarySE ) ); 	}  }    protected ShapeText text57;  protected ShapeText text58;    /**   * <i>This method should not be called by user</i>   */  @AnyLogicInternalCodegenAPI  private void _text59_SetDynamicParams_xjal( ShapeText shape ) {    boolean _visible = getCurrentIteration() > 0 ;    shape.setVisible( _visible ); 	if ( _visible ) {    shape.setText(format( logIncomeSqSE ) ); 	}  }    protected ShapeText text59;  protected ShapeText text60;    /**   * <i>This method should not be called by user</i>   */  @AnyLogicInternalCodegenAPI  private void _text61_SetDynamicParams_xjal( ShapeText shape ) {    boolean _visible = getCurrentIteration() > 0 ;    shape.setVisible( _visible ); 	if ( _visible ) {    shape.setText(format( logIncomeSE ) ); 	}  }    protected ShapeText text61;  protected ShapeText text62;    /**   * <i>This method should not be called by user</i>   */  @AnyLogicInternalCodegenAPI  private void _text63_SetDynamicParams_xjal( ShapeText shape ) {    boolean _visible = getCurrentIteration() > 0 ;    shape.setVisible( _visible ); 	if ( _visible ) {    shape.setText(format( iv ) ); 	}  }    protected ShapeText text63;  protected ShapeText text64;    /**   * <i>This method should not be called by user</i>   */  @AnyLogicInternalCodegenAPI  private void _text65_SetDynamicParams_xjal( ShapeText shape ) {    boolean _visible = getCurrentIteration() > 0 ;    shape.setVisible( _visible ); 	if ( _visible ) {    shape.setText(format( ivSE ) ); 	}  }    protected ShapeText text65;  protected ShapeText text66;    /**   * <i>This method should not be called by user</i>   */  @AnyLogicInternalCodegenAPI  private void _text67_SetDynamicParams_xjal( ShapeText shape ) {    boolean _visible = getCurrentIteration() > 0 ;    shape.setVisible( _visible ); 	if ( _visible ) {    shape.setText(format( phi ) ); 	}  }    protected ShapeText text67;  protected ShapeText text68;    /**   * <i>This method should not be called by user</i>   */  @AnyLogicInternalCodegenAPI  private void _text69_SetDynamicParams_xjal( ShapeText shape ) {    boolean _visible = getCurrentIteration() > 0 ;    shape.setVisible( _visible ); 	if ( _visible ) {    shape.setText(format( voucher ) ); 	}  }    protected ShapeText text69;  @AnyLogicInternalCodegenAPI  private void _createPersistentElementsBP0_xjal() {    button = new ShapeButton(			ParamVar.this, true, 40.0, 80.0, 			100.0, 30.0,            controlDefault, controlDefault, true,            _button_Font, 			"Run" ) {      @Override      public void updateDynamicProperties(boolean publicOnly) {        _button_SetDynamicParams_xjal( this );        super.updateDynamicProperties(publicOnly);      }      @Override      @AnyLogicInternalCodegenAPI      public void action(){        executeShapeControlAction( _button, 0 );      }    };    text = new ShapeText(        SHAPE_DRAW_2D, true,40.0, 30.0, 0.0, 0.0,         royalBlue,"Guinea CTI optimization 2018-01-22 : ParamVar",        _text_Font, ALIGNMENT_LEFT );    text1 = new ShapeText(        SHAPE_DRAW_2D, true,50.0, 130.0, 0.0, 0.0,         black,"Iteration:",        _text1_Font, ALIGNMENT_LEFT );    text2 = new ShapeText(        SHAPE_DRAW_2D, true,270.0, 130.0, 0.0, 0.0,         darkSlateBlue,"?",        _text2_Font, ALIGNMENT_RIGHT ) {      @Override      public void updateDynamicProperties(boolean publicOnly) {        _text2_SetDynamicParams_xjal( this );        super.updateDynamicProperties(publicOnly);      }    };    text3 = new ShapeText(        SHAPE_DRAW_2D, true,50.0, 150.0, 0.0, 0.0,         black,"Replication:",        _text3_Font, ALIGNMENT_LEFT );    text4 = new ShapeText(        SHAPE_DRAW_2D, true,270.0, 150.0, 0.0, 0.0,         darkSlateBlue,"?",        _text4_Font, ALIGNMENT_RIGHT ) {      @Override      public void updateDynamicProperties(boolean publicOnly) {        _text4_SetDynamicParams_xjal( this );        super.updateDynamicProperties(publicOnly);      }    };    line = new ShapeLine(		SHAPE_DRAW_2D, true, 40.0, 170.0, 0.0, black,  		240.0,	0.0, 0.0, 1.0, 10.0, LINE_STYLE_SOLID );    text5 = new ShapeText(        SHAPE_DRAW_2D, true,50.0, 180.0, 0.0, 0.0,         black,"Parameters",        _text5_Font, ALIGNMENT_LEFT );    text6 = new ShapeText(        SHAPE_DRAW_2D, true,50.0, 210.0, 0.0, 0.0,         black,"male",        _text6_Font, ALIGNMENT_LEFT );    text7 = new ShapeText(        SHAPE_DRAW_2D, true,270.0, 210.0, 0.0, 0.0,         darkSlateBlue,"?",        _text7_Font, ALIGNMENT_RIGHT ) {      @Override      public void updateDynamicProperties(boolean publicOnly) {        _text7_SetDynamicParams_xjal( this );        super.updateDynamicProperties(publicOnly);      }    };    text8 = new ShapeText(        SHAPE_DRAW_2D, true,50.0, 230.0, 0.0, 0.0,         black,"distance",        _text8_Font, ALIGNMENT_LEFT );    text9 = new ShapeText(        SHAPE_DRAW_2D, true,270.0, 230.0, 0.0, 0.0,         darkSlateBlue,"?",        _text9_Font, ALIGNMENT_RIGHT ) {      @Override      public void updateDynamicProperties(boolean publicOnly) {        _text9_SetDynamicParams_xjal( this );        super.updateDynamicProperties(publicOnly);      }    };    text10 = new ShapeText(        SHAPE_DRAW_2D, true,50.0, 250.0, 0.0, 0.0,         black,"educPrimaryComplete",        _text10_Font, ALIGNMENT_LEFT );    text11 = new ShapeText(        SHAPE_DRAW_2D, true,270.0, 250.0, 0.0, 0.0,         darkSlateBlue,"?",        _text11_Font, ALIGNMENT_RIGHT ) {      @Override      public void updateDynamicProperties(boolean publicOnly) {        _text11_SetDynamicParams_xjal( this );        super.updateDynamicProperties(publicOnly);      }    };    text12 = new ShapeText(        SHAPE_DRAW_2D, true,50.0, 270.0, 0.0, 0.0,         black,"educPrimary",        _text12_Font, ALIGNMENT_LEFT );    text13 = new ShapeText(        SHAPE_DRAW_2D, true,270.0, 270.0, 0.0, 0.0,         darkSlateBlue,"?",        _text13_Font, ALIGNMENT_RIGHT ) {      @Override      public void updateDynamicProperties(boolean publicOnly) {        _text13_SetDynamicParams_xjal( this );        super.updateDynamicProperties(publicOnly);      }    };    text14 = new ShapeText(        SHAPE_DRAW_2D, true,50.0, 290.0, 0.0, 0.0,         black,"age50",        _text14_Font, ALIGNMENT_LEFT );    text15 = new ShapeText(        SHAPE_DRAW_2D, true,270.0, 290.0, 0.0, 0.0,         darkSlateBlue,"?",        _text15_Font, ALIGNMENT_RIGHT ) {      @Override      public void updateDynamicProperties(boolean publicOnly) {        _text15_SetDynamicParams_xjal( this );        super.updateDynamicProperties(publicOnly);      }    };    text16 = new ShapeText(        SHAPE_DRAW_2D, true,50.0, 310.0, 0.0, 0.0,         black,"age2249",        _text16_Font, ALIGNMENT_LEFT );    text17 = new ShapeText(        SHAPE_DRAW_2D, true,270.0, 310.0, 0.0, 0.0,         darkSlateBlue,"?",        _text17_Font, ALIGNMENT_RIGHT ) {      @Override      public void updateDynamicProperties(boolean publicOnly) {        _text17_SetDynamicParams_xjal( this );        super.updateDynamicProperties(publicOnly);      }    };    text18 = new ShapeText(        SHAPE_DRAW_2D, true,50.0, 330.0, 0.0, 0.0,         black,"age1521",        _text18_Font, ALIGNMENT_LEFT );    text19 = new ShapeText(        SHAPE_DRAW_2D, true,270.0, 330.0, 0.0, 0.0,         darkSlateBlue,"?",        _text19_Font, ALIGNMENT_RIGHT ) {      @Override      public void updateDynamicProperties(boolean publicOnly) {        _text19_SetDynamicParams_xjal( this );        super.updateDynamicProperties(publicOnly);      }    };    text20 = new ShapeText(        SHAPE_DRAW_2D, true,50.0, 350.0, 0.0, 0.0,         black,"age514",        _text20_Font, ALIGNMENT_LEFT );    text21 = new ShapeText(        SHAPE_DRAW_2D, true,270.0, 350.0, 0.0, 0.0,         darkSlateBlue,"?",        _text21_Font, ALIGNMENT_RIGHT ) {      @Override      public void updateDynamicProperties(boolean publicOnly) {        _text21_SetDynamicParams_xjal( this );        super.updateDynamicProperties(publicOnly);      }    };    text22 = new ShapeText(        SHAPE_DRAW_2D, true,50.0, 370.0, 0.0, 0.0,         black,"q",        _text22_Font, ALIGNMENT_LEFT );    text23 = new ShapeText(        SHAPE_DRAW_2D, true,270.0, 370.0, 0.0, 0.0,         darkSlateBlue,"?",        _text23_Font, ALIGNMENT_RIGHT ) {      @Override      public void updateDynamicProperties(boolean publicOnly) {        _text23_SetDynamicParams_xjal( this );        super.updateDynamicProperties(publicOnly);      }    };    text24 = new ShapeText(        SHAPE_DRAW_2D, true,50.0, 390.0, 0.0, 0.0,         black,"intercept",        _text24_Font, ALIGNMENT_LEFT );    text25 = new ShapeText(        SHAPE_DRAW_2D, true,270.0, 390.0, 0.0, 0.0,         darkSlateBlue,"?",        _text25_Font, ALIGNMENT_RIGHT ) {      @Override      public void updateDynamicProperties(boolean publicOnly) {        _text25_SetDynamicParams_xjal( this );        super.updateDynamicProperties(publicOnly);      }    };    text26 = new ShapeText(        SHAPE_DRAW_2D, true,50.0, 410.0, 0.0, 0.0,         black,"educSecondaryComplete",        _text26_Font, ALIGNMENT_LEFT );    text27 = new ShapeText(        SHAPE_DRAW_2D, true,270.0, 410.0, 0.0, 0.0,         darkSlateBlue,"?",        _text27_Font, ALIGNMENT_RIGHT ) {      @Override      public void updateDynamicProperties(boolean publicOnly) {        _text27_SetDynamicParams_xjal( this );        super.updateDynamicProperties(publicOnly);      }    };    text28 = new ShapeText(        SHAPE_DRAW_2D, true,50.0, 430.0, 0.0, 0.0,         black,"educSecondary",        _text28_Font, ALIGNMENT_LEFT );    text29 = new ShapeText(        SHAPE_DRAW_2D, true,270.0, 430.0, 0.0, 0.0,         darkSlateBlue,"?",        _text29_Font, ALIGNMENT_RIGHT ) {      @Override      public void updateDynamicProperties(boolean publicOnly) {        _text29_SetDynamicParams_xjal( this );        super.updateDynamicProperties(publicOnly);      }    };    text30 = new ShapeText(        SHAPE_DRAW_2D, true,50.0, 450.0, 0.0, 0.0,         black,"logIncomeSq",        _text30_Font, ALIGNMENT_LEFT );    text31 = new ShapeText(        SHAPE_DRAW_2D, true,270.0, 450.0, 0.0, 0.0,         darkSlateBlue,"?",        _text31_Font, ALIGNMENT_RIGHT ) {      @Override      public void updateDynamicProperties(boolean publicOnly) {        _text31_SetDynamicParams_xjal( this );        super.updateDynamicProperties(publicOnly);      }    };    text32 = new ShapeText(        SHAPE_DRAW_2D, true,50.0, 470.0, 0.0, 0.0,         black,"logIncome",        _text32_Font, ALIGNMENT_LEFT );    text33 = new ShapeText(        SHAPE_DRAW_2D, true,270.0, 470.0, 0.0, 0.0,         darkSlateBlue,"?",        _text33_Font, ALIGNMENT_RIGHT ) {      @Override      public void updateDynamicProperties(boolean publicOnly) {        _text33_SetDynamicParams_xjal( this );        super.updateDynamicProperties(publicOnly);      }    };    text34 = new ShapeText(        SHAPE_DRAW_2D, true,50.0, 490.0, 0.0, 0.0,         black,"maleSE",        _text34_Font, ALIGNMENT_LEFT );    text35 = new ShapeText(        SHAPE_DRAW_2D, true,270.0, 490.0, 0.0, 0.0,         darkSlateBlue,"?",        _text35_Font, ALIGNMENT_RIGHT ) {      @Override      public void updateDynamicProperties(boolean publicOnly) {        _text35_SetDynamicParams_xjal( this );        super.updateDynamicProperties(publicOnly);      }    };    text36 = new ShapeText(        SHAPE_DRAW_2D, true,50.0, 510.0, 0.0, 0.0,         black,"distanceSE",        _text36_Font, ALIGNMENT_LEFT );    text37 = new ShapeText(        SHAPE_DRAW_2D, true,270.0, 510.0, 0.0, 0.0,         darkSlateBlue,"?",        _text37_Font, ALIGNMENT_RIGHT ) {      @Override      public void updateDynamicProperties(boolean publicOnly) {        _text37_SetDynamicParams_xjal( this );        super.updateDynamicProperties(publicOnly);      }    };    text38 = new ShapeText(        SHAPE_DRAW_2D, true,50.0, 530.0, 0.0, 0.0,         black,"educPrimaryCompleteSE",        _text38_Font, ALIGNMENT_LEFT );    text39 = new ShapeText(        SHAPE_DRAW_2D, true,270.0, 530.0, 0.0, 0.0,         darkSlateBlue,"?",        _text39_Font, ALIGNMENT_RIGHT ) {      @Override      public void updateDynamicProperties(boolean publicOnly) {        _text39_SetDynamicParams_xjal( this );        super.updateDynamicProperties(publicOnly);      }    };    text40 = new ShapeText(        SHAPE_DRAW_2D, true,50.0, 550.0, 0.0, 0.0,         black,"educPrimarySE",        _text40_Font, ALIGNMENT_LEFT );    text41 = new ShapeText(        SHAPE_DRAW_2D, true,270.0, 550.0, 0.0, 0.0,         darkSlateBlue,"?",        _text41_Font, ALIGNMENT_RIGHT ) {      @Override      public void updateDynamicProperties(boolean publicOnly) {        _text41_SetDynamicParams_xjal( this );        super.updateDynamicProperties(publicOnly);      }    };    text42 = new ShapeText(        SHAPE_DRAW_2D, true,50.0, 570.0, 0.0, 0.0,         black,"age50SE",        _text42_Font, ALIGNMENT_LEFT );    text43 = new ShapeText(        SHAPE_DRAW_2D, true,270.0, 570.0, 0.0, 0.0,         darkSlateBlue,"?",        _text43_Font, ALIGNMENT_RIGHT ) {      @Override      public void updateDynamicProperties(boolean publicOnly) {        _text43_SetDynamicParams_xjal( this );        super.updateDynamicProperties(publicOnly);      }    };    text44 = new ShapeText(        SHAPE_DRAW_2D, true,50.0, 590.0, 0.0, 0.0,         black,"age2249SE",        _text44_Font, ALIGNMENT_LEFT );    text45 = new ShapeText(        SHAPE_DRAW_2D, true,270.0, 590.0, 0.0, 0.0,         darkSlateBlue,"?",        _text45_Font, ALIGNMENT_RIGHT ) {      @Override      public void updateDynamicProperties(boolean publicOnly) {        _text45_SetDynamicParams_xjal( this );        super.updateDynamicProperties(publicOnly);      }    };    text46 = new ShapeText(        SHAPE_DRAW_2D, true,50.0, 610.0, 0.0, 0.0,         black,"age1521SE",        _text46_Font, ALIGNMENT_LEFT );    text47 = new ShapeText(        SHAPE_DRAW_2D, true,270.0, 610.0, 0.0, 0.0,         darkSlateBlue,"?",        _text47_Font, ALIGNMENT_RIGHT ) {      @Override      public void updateDynamicProperties(boolean publicOnly) {        _text47_SetDynamicParams_xjal( this );        super.updateDynamicProperties(publicOnly);      }    };    text48 = new ShapeText(        SHAPE_DRAW_2D, true,50.0, 630.0, 0.0, 0.0,         black,"age514SE",        _text48_Font, ALIGNMENT_LEFT );    text49 = new ShapeText(        SHAPE_DRAW_2D, true,270.0, 630.0, 0.0, 0.0,         darkSlateBlue,"?",        _text49_Font, ALIGNMENT_RIGHT ) {      @Override      public void updateDynamicProperties(boolean publicOnly) {        _text49_SetDynamicParams_xjal( this );        super.updateDynamicProperties(publicOnly);      }    };    text50 = new ShapeText(        SHAPE_DRAW_2D, true,50.0, 650.0, 0.0, 0.0,         black,"qSE",        _text50_Font, ALIGNMENT_LEFT );    text51 = new ShapeText(        SHAPE_DRAW_2D, true,270.0, 650.0, 0.0, 0.0,         darkSlateBlue,"?",        _text51_Font, ALIGNMENT_RIGHT ) {      @Override      public void updateDynamicProperties(boolean publicOnly) {        _text51_SetDynamicParams_xjal( this );        super.updateDynamicProperties(publicOnly);      }    };    text52 = new ShapeText(        SHAPE_DRAW_2D, true,50.0, 670.0, 0.0, 0.0,         black,"interceptSE",        _text52_Font, ALIGNMENT_LEFT );    text53 = new ShapeText(        SHAPE_DRAW_2D, true,270.0, 670.0, 0.0, 0.0,         darkSlateBlue,"?",        _text53_Font, ALIGNMENT_RIGHT ) {      @Override      public void updateDynamicProperties(boolean publicOnly) {        _text53_SetDynamicParams_xjal( this );        super.updateDynamicProperties(publicOnly);      }    };    text54 = new ShapeText(        SHAPE_DRAW_2D, true,50.0, 690.0, 0.0, 0.0,         black,"educSecondaryCompleteSE",        _text54_Font, ALIGNMENT_LEFT );    text55 = new ShapeText(        SHAPE_DRAW_2D, true,270.0, 690.0, 0.0, 0.0,         darkSlateBlue,"?",        _text55_Font, ALIGNMENT_RIGHT ) {      @Override      public void updateDynamicProperties(boolean publicOnly) {        _text55_SetDynamicParams_xjal( this );        super.updateDynamicProperties(publicOnly);      }    };    text56 = new ShapeText(        SHAPE_DRAW_2D, true,50.0, 710.0, 0.0, 0.0,         black,"educSecondarySE",        _text56_Font, ALIGNMENT_LEFT );    text57 = new ShapeText(        SHAPE_DRAW_2D, true,270.0, 710.0, 0.0, 0.0,         darkSlateBlue,"?",        _text57_Font, ALIGNMENT_RIGHT ) {      @Override      public void updateDynamicProperties(boolean publicOnly) {        _text57_SetDynamicParams_xjal( this );        super.updateDynamicProperties(publicOnly);      }    };    text58 = new ShapeText(        SHAPE_DRAW_2D, true,50.0, 730.0, 0.0, 0.0,         black,"logIncomeSqSE",        _text58_Font, ALIGNMENT_LEFT );    text59 = new ShapeText(        SHAPE_DRAW_2D, true,270.0, 730.0, 0.0, 0.0,         darkSlateBlue,"?",        _text59_Font, ALIGNMENT_RIGHT ) {      @Override      public void updateDynamicProperties(boolean publicOnly) {        _text59_SetDynamicParams_xjal( this );        super.updateDynamicProperties(publicOnly);      }    };    text60 = new ShapeText(        SHAPE_DRAW_2D, true,50.0, 750.0, 0.0, 0.0,         black,"logIncomeSE",        _text60_Font, ALIGNMENT_LEFT );    text61 = new ShapeText(        SHAPE_DRAW_2D, true,270.0, 750.0, 0.0, 0.0,         darkSlateBlue,"?",        _text61_Font, ALIGNMENT_RIGHT ) {      @Override      public void updateDynamicProperties(boolean publicOnly) {        _text61_SetDynamicParams_xjal( this );        super.updateDynamicProperties(publicOnly);      }    };    text62 = new ShapeText(        SHAPE_DRAW_2D, true,50.0, 770.0, 0.0, 0.0,         black,"iv",        _text62_Font, ALIGNMENT_LEFT );    text63 = new ShapeText(        SHAPE_DRAW_2D, true,270.0, 770.0, 0.0, 0.0,         darkSlateBlue,"?",        _text63_Font, ALIGNMENT_RIGHT ) {      @Override      public void updateDynamicProperties(boolean publicOnly) {        _text63_SetDynamicParams_xjal( this );        super.updateDynamicProperties(publicOnly);      }    };    text64 = new ShapeText(        SHAPE_DRAW_2D, true,50.0, 790.0, 0.0, 0.0,         black,"ivSE",        _text64_Font, ALIGNMENT_LEFT );    text65 = new ShapeText(        SHAPE_DRAW_2D, true,270.0, 790.0, 0.0, 0.0,         darkSlateBlue,"?",        _text65_Font, ALIGNMENT_RIGHT ) {      @Override      public void updateDynamicProperties(boolean publicOnly) {        _text65_SetDynamicParams_xjal( this );        super.updateDynamicProperties(publicOnly);      }    };    text66 = new ShapeText(        SHAPE_DRAW_2D, true,50.0, 810.0, 0.0, 0.0,         black,"phi",        _text66_Font, ALIGNMENT_LEFT );    text67 = new ShapeText(        SHAPE_DRAW_2D, true,270.0, 810.0, 0.0, 0.0,         darkSlateBlue,"?",        _text67_Font, ALIGNMENT_RIGHT ) {      @Override      public void updateDynamicProperties(boolean publicOnly) {        _text67_SetDynamicParams_xjal( this );        super.updateDynamicProperties(publicOnly);      }    };    text68 = new ShapeText(        SHAPE_DRAW_2D, true,290.0, 150.0, 0.0, 0.0,         black,"voucher",        _text68_Font, ALIGNMENT_LEFT );    text69 = new ShapeText(        SHAPE_DRAW_2D, true,510.0, 150.0, 0.0, 0.0,         darkSlateBlue,"?",        _text69_Font, ALIGNMENT_RIGHT ) {      @Override      public void updateDynamicProperties(boolean publicOnly) {        _text69_SetDynamicParams_xjal( this );        super.updateDynamicProperties(publicOnly);      }    };  }  @AnyLogicInternalCodegenAPI  private void _createPersistentElementsAP0_xjal() {  }  protected ShapeTopLevelPresentationGroup presentation;  protected ShapeModelElementsGroup icon;   @Override  @AnyLogicInternalCodegenAPI  public ShapeTopLevelPresentationGroup getPresentationShape() {    return presentation;  }  @Override  @AnyLogicInternalCodegenAPI  public ShapeModelElementsGroup getModelElementsShape() {    return icon;  }  @Override  @AnyLogicInternalCodegenAPI  public Object getPersistentShape( int _shape ) {    switch (_shape) {      case _presentation: return presentation;      case _icon: return icon;      case _button: return button;      case _text: return text;      case _text1: return text1;      case _text2: return text2;      case _text3: return text3;      case _text4: return text4;      case _line: return line;      case _text5: return text5;      case _text6: return text6;      case _text7: return text7;      case _text8: return text8;      case _text9: return text9;      case _text10: return text10;      case _text11: return text11;      case _text12: return text12;      case _text13: return text13;      case _text14: return text14;      case _text15: return text15;      case _text16: return text16;      case _text17: return text17;      case _text18: return text18;      case _text19: return text19;      case _text20: return text20;      case _text21: return text21;      case _text22: return text22;      case _text23: return text23;      case _text24: return text24;      case _text25: return text25;      case _text26: return text26;      case _text27: return text27;      case _text28: return text28;      case _text29: return text29;      case _text30: return text30;      case _text31: return text31;      case _text32: return text32;      case _text33: return text33;      case _text34: return text34;      case _text35: return text35;      case _text36: return text36;      case _text37: return text37;      case _text38: return text38;      case _text39: return text39;      case _text40: return text40;      case _text41: return text41;      case _text42: return text42;      case _text43: return text43;      case _text44: return text44;      case _text45: return text45;      case _text46: return text46;      case _text47: return text47;      case _text48: return text48;      case _text49: return text49;      case _text50: return text50;      case _text51: return text51;      case _text52: return text52;      case _text53: return text53;      case _text54: return text54;      case _text55: return text55;      case _text56: return text56;      case _text57: return text57;      case _text58: return text58;      case _text59: return text59;      case _text60: return text60;      case _text61: return text61;      case _text62: return text62;      case _text63: return text63;      case _text64: return text64;      case _text65: return text65;      case _text66: return text66;      case _text67: return text67;      case _text68: return text68;      case _text69: return text69;      default: return super.getPersistentShape( _shape );     }  }  @Override  @AnyLogicInternalCodegenAPI  public String getNameOfShape_xjal( Object _shape ) {    try {      if ( _shape == null ) return null;      String _name_xjal;      _name_xjal = checkNameOfShape_xjal( _shape, presentation, "presentation" ); if (_name_xjal != null) return _name_xjal;      _name_xjal = checkNameOfShape_xjal( _shape, icon, "icon" ); if (_name_xjal != null) return _name_xjal;      _name_xjal = checkNameOfShape_xjal( _shape, button, "button" ); if (_name_xjal != null) return _name_xjal;      _name_xjal = checkNameOfShape_xjal( _shape, text, "text" ); if (_name_xjal != null) return _name_xjal;      _name_xjal = checkNameOfShape_xjal( _shape, text1, "text1" ); if (_name_xjal != null) return _name_xjal;      _name_xjal = checkNameOfShape_xjal( _shape, text2, "text2" ); if (_name_xjal != null) return _name_xjal;      _name_xjal = checkNameOfShape_xjal( _shape, text3, "text3" ); if (_name_xjal != null) return _name_xjal;      _name_xjal = checkNameOfShape_xjal( _shape, text4, "text4" ); if (_name_xjal != null) return _name_xjal;      _name_xjal = checkNameOfShape_xjal( _shape, line, "line" ); if (_name_xjal != null) return _name_xjal;      _name_xjal = checkNameOfShape_xjal( _shape, text5, "text5" ); if (_name_xjal != null) return _name_xjal;      _name_xjal = checkNameOfShape_xjal( _shape, text6, "text6" ); if (_name_xjal != null) return _name_xjal;      _name_xjal = checkNameOfShape_xjal( _shape, text7, "text7" ); if (_name_xjal != null) return _name_xjal;      _name_xjal = checkNameOfShape_xjal( _shape, text8, "text8" ); if (_name_xjal != null) return _name_xjal;      _name_xjal = checkNameOfShape_xjal( _shape, text9, "text9" ); if (_name_xjal != null) return _name_xjal;      _name_xjal = checkNameOfShape_xjal( _shape, text10, "text10" ); if (_name_xjal != null) return _name_xjal;      _name_xjal = checkNameOfShape_xjal( _shape, text11, "text11" ); if (_name_xjal != null) return _name_xjal;      _name_xjal = checkNameOfShape_xjal( _shape, text12, "text12" ); if (_name_xjal != null) return _name_xjal;      _name_xjal = checkNameOfShape_xjal( _shape, text13, "text13" ); if (_name_xjal != null) return _name_xjal;      _name_xjal = checkNameOfShape_xjal( _shape, text14, "text14" ); if (_name_xjal != null) return _name_xjal;      _name_xjal = checkNameOfShape_xjal( _shape, text15, "text15" ); if (_name_xjal != null) return _name_xjal;      _name_xjal = checkNameOfShape_xjal( _shape, text16, "text16" ); if (_name_xjal != null) return _name_xjal;      _name_xjal = checkNameOfShape_xjal( _shape, text17, "text17" ); if (_name_xjal != null) return _name_xjal;      _name_xjal = checkNameOfShape_xjal( _shape, text18, "text18" ); if (_name_xjal != null) return _name_xjal;      _name_xjal = checkNameOfShape_xjal( _shape, text19, "text19" ); if (_name_xjal != null) return _name_xjal;      _name_xjal = checkNameOfShape_xjal( _shape, text20, "text20" ); if (_name_xjal != null) return _name_xjal;      _name_xjal = checkNameOfShape_xjal( _shape, text21, "text21" ); if (_name_xjal != null) return _name_xjal;      _name_xjal = checkNameOfShape_xjal( _shape, text22, "text22" ); if (_name_xjal != null) return _name_xjal;      _name_xjal = checkNameOfShape_xjal( _shape, text23, "text23" ); if (_name_xjal != null) return _name_xjal;      _name_xjal = checkNameOfShape_xjal( _shape, text24, "text24" ); if (_name_xjal != null) return _name_xjal;      _name_xjal = checkNameOfShape_xjal( _shape, text25, "text25" ); if (_name_xjal != null) return _name_xjal;      _name_xjal = checkNameOfShape_xjal( _shape, text26, "text26" ); if (_name_xjal != null) return _name_xjal;      _name_xjal = checkNameOfShape_xjal( _shape, text27, "text27" ); if (_name_xjal != null) return _name_xjal;      _name_xjal = checkNameOfShape_xjal( _shape, text28, "text28" ); if (_name_xjal != null) return _name_xjal;      _name_xjal = checkNameOfShape_xjal( _shape, text29, "text29" ); if (_name_xjal != null) return _name_xjal;      _name_xjal = checkNameOfShape_xjal( _shape, text30, "text30" ); if (_name_xjal != null) return _name_xjal;      _name_xjal = checkNameOfShape_xjal( _shape, text31, "text31" ); if (_name_xjal != null) return _name_xjal;      _name_xjal = checkNameOfShape_xjal( _shape, text32, "text32" ); if (_name_xjal != null) return _name_xjal;      _name_xjal = checkNameOfShape_xjal( _shape, text33, "text33" ); if (_name_xjal != null) return _name_xjal;      _name_xjal = checkNameOfShape_xjal( _shape, text34, "text34" ); if (_name_xjal != null) return _name_xjal;      _name_xjal = checkNameOfShape_xjal( _shape, text35, "text35" ); if (_name_xjal != null) return _name_xjal;      _name_xjal = checkNameOfShape_xjal( _shape, text36, "text36" ); if (_name_xjal != null) return _name_xjal;      _name_xjal = checkNameOfShape_xjal( _shape, text37, "text37" ); if (_name_xjal != null) return _name_xjal;      _name_xjal = checkNameOfShape_xjal( _shape, text38, "text38" ); if (_name_xjal != null) return _name_xjal;      _name_xjal = checkNameOfShape_xjal( _shape, text39, "text39" ); if (_name_xjal != null) return _name_xjal;      _name_xjal = checkNameOfShape_xjal( _shape, text40, "text40" ); if (_name_xjal != null) return _name_xjal;      _name_xjal = checkNameOfShape_xjal( _shape, text41, "text41" ); if (_name_xjal != null) return _name_xjal;      _name_xjal = checkNameOfShape_xjal( _shape, text42, "text42" ); if (_name_xjal != null) return _name_xjal;      _name_xjal = checkNameOfShape_xjal( _shape, text43, "text43" ); if (_name_xjal != null) return _name_xjal;      _name_xjal = checkNameOfShape_xjal( _shape, text44, "text44" ); if (_name_xjal != null) return _name_xjal;      _name_xjal = checkNameOfShape_xjal( _shape, text45, "text45" ); if (_name_xjal != null) return _name_xjal;      _name_xjal = checkNameOfShape_xjal( _shape, text46, "text46" ); if (_name_xjal != null) return _name_xjal;      _name_xjal = checkNameOfShape_xjal( _shape, text47, "text47" ); if (_name_xjal != null) return _name_xjal;      _name_xjal = checkNameOfShape_xjal( _shape, text48, "text48" ); if (_name_xjal != null) return _name_xjal;      _name_xjal = checkNameOfShape_xjal( _shape, text49, "text49" ); if (_name_xjal != null) return _name_xjal;      _name_xjal = checkNameOfShape_xjal( _shape, text50, "text50" ); if (_name_xjal != null) return _name_xjal;      _name_xjal = checkNameOfShape_xjal( _shape, text51, "text51" ); if (_name_xjal != null) return _name_xjal;      _name_xjal = checkNameOfShape_xjal( _shape, text52, "text52" ); if (_name_xjal != null) return _name_xjal;      _name_xjal = checkNameOfShape_xjal( _shape, text53, "text53" ); if (_name_xjal != null) return _name_xjal;      _name_xjal = checkNameOfShape_xjal( _shape, text54, "text54" ); if (_name_xjal != null) return _name_xjal;      _name_xjal = checkNameOfShape_xjal( _shape, text55, "text55" ); if (_name_xjal != null) return _name_xjal;      _name_xjal = checkNameOfShape_xjal( _shape, text56, "text56" ); if (_name_xjal != null) return _name_xjal;      _name_xjal = checkNameOfShape_xjal( _shape, text57, "text57" ); if (_name_xjal != null) return _name_xjal;      _name_xjal = checkNameOfShape_xjal( _shape, text58, "text58" ); if (_name_xjal != null) return _name_xjal;      _name_xjal = checkNameOfShape_xjal( _shape, text59, "text59" ); if (_name_xjal != null) return _name_xjal;      _name_xjal = checkNameOfShape_xjal( _shape, text60, "text60" ); if (_name_xjal != null) return _name_xjal;      _name_xjal = checkNameOfShape_xjal( _shape, text61, "text61" ); if (_name_xjal != null) return _name_xjal;      _name_xjal = checkNameOfShape_xjal( _shape, text62, "text62" ); if (_name_xjal != null) return _name_xjal;      _name_xjal = checkNameOfShape_xjal( _shape, text63, "text63" ); if (_name_xjal != null) return _name_xjal;      _name_xjal = checkNameOfShape_xjal( _shape, text64, "text64" ); if (_name_xjal != null) return _name_xjal;      _name_xjal = checkNameOfShape_xjal( _shape, text65, "text65" ); if (_name_xjal != null) return _name_xjal;      _name_xjal = checkNameOfShape_xjal( _shape, text66, "text66" ); if (_name_xjal != null) return _name_xjal;      _name_xjal = checkNameOfShape_xjal( _shape, text67, "text67" ); if (_name_xjal != null) return _name_xjal;      _name_xjal = checkNameOfShape_xjal( _shape, text68, "text68" ); if (_name_xjal != null) return _name_xjal;      _name_xjal = checkNameOfShape_xjal( _shape, text69, "text69" ); if (_name_xjal != null) return _name_xjal;    } catch (Exception e) {      return null;    }    return super.getNameOfShape_xjal( _shape );  }  @Override  public int getWindowWidth() {    return 1550;  }  @Override  public int getWindowHeight() {    return 890;  }    @Override  @AnyLogicInternalCodegenAPI  public void onDestroy_xjal() {    // Destroy database objects    paramVar.writeFile();    super.onDestroy_xjal();  }  /**   * Applet class to run experiment as java applet   */    @AnyLogicInternalCodegenAPI  public static class Applet extends JApplet {  @AnyLogicInternalCodegenAPI    ParamVar ex;    @Override    public void init() {      ex = new ParamVar();      ex.setup( this );    }    @Override    public void destroy() {      ex.close();    }  }  @Override  @AnyLogicInternalCodegenAPI  public void initDefaultRandomNumberGenerator(Engine _e) {    _e.setDefaultRandomGenerator( new java.util.Random() );  }  // Parameter values (read-only)  public double male;  public double distance;  public double educPrimaryComplete;  public double educPrimary;  public double age50;  public double age2249;  public double age1521;  public double age514;  public double q;  public double intercept;  public double educSecondaryComplete;  public double educSecondary;  public double logIncomeSq;  public double logIncome;  public double maleSE;  public double distanceSE;  public double educPrimaryCompleteSE;  public double educPrimarySE;  public double age50SE;  public double age2249SE;  public double age1521SE;  public double age514SE;  public double qSE;  public double interceptSE;  public double educSecondaryCompleteSE;  public double educSecondarySE;  public double logIncomeSqSE;  public double logIncomeSE;  public double iv;  public double ivSE;  public double phi;  public double voucher;  /**   * Engine setup   */  @Override  @AnyLogicInternalCodegenAPI  public void setupEngine(Engine engine) {    engine.setATOL( 1.0E-5 );    engine.setRTOL( 1.0E-5 );    engine.setTTOL( 1.0E-5 );    engine.setHTOL( 0.001 );    engine.setSolverODE( Engine.SOLVER_ODE_EULER );    engine.setSolverNAE( Engine.SOLVER_NAE_MODIFIED_NEWTON );    engine.setSolverDAE( Engine.SOLVER_DAE_RK45_NEWTON );    engine.setVMethods( 427829 );    engine.setSimultaneousEventsSelectionMode( Engine.EVENT_SELECTION_LIFO );    engine.setStartTime( 0.0 );    engine.setTimeUnit( SECOND );    engine.setStartDate( toDate( 2017, JUNE, 4, 0, 0, 0 ) );    engine.setStopTime( 100.0 );  }  /**   * Experiment setup   */  @Override  @AnyLogicInternalCodegenAPI  public void setup( java.awt.Container container ) {    setName( "Guinea CTI optimization 2018-01-22 : ParamVar" );    Presentation _p = new Presentation( this, container instanceof JApplet ? Presentation.MODE_APPLET :    	container != null ? Presentation.MODE_COMPONENT :        Presentation.MODE_APPLICATION, container );    // Static initialization of persistent elements    _createPersistentElementsBP0_xjal();    paramVar.readFile();    // Dynamic initialization of persistent elements    _createPersistentElementsAP0_xjal();    presentation = new ShapeTopLevelPresentationGroup( ParamVar.this, true, 0, 0, 0, 0 , text, text1, text2, text3, text4, line, text5, text6, text7, text8, text9, text10, text11, text12, text13, text14, text15, text16, text17, text18, text19, text20, text21, text22, text23, text24, text25, text26, text27, text28, text29, text30, text31, text32, text33, text34, text35, text36, text37, text38, text39, text40, text41, text42, text43, text44, text45, text46, text47, text48, text49, text50, text51, text52, text53, text54, text55, text56, text57, text58, text59, text60, text61, text62, text63, text64, text65, text66, text67, text68, text69, button );    icon = new ShapeModelElementsGroup( ParamVar.this, getElementProperty( "malawi_recentralization_model_2017_06_04.ParamVar.icon", IElementDescriptor.MODEL_ELEMENT_DESCRIPTORS )  );    // Setup presentation    _p.start();    Panel _panel = _p.getPanel();    ToolBar _tb = _p.getToolBar();    StatusBar _sb = _p.getStatusBar();    _panel.setFrameManagementBalance( 2.0 );    _sb.setSectionVisible( StatusBar.EPS, false );    _sb.setSectionVisible( StatusBar.EXPERIMENT, true );    _sb.setSectionVisible( StatusBar.FPS, false );    _sb.setSectionVisible( StatusBar.MEMORY, true );    _sb.setSectionVisible( StatusBar.SECONDS, true );    _sb.setSectionVisible( StatusBar.SIMULATION, true );    _sb.setSectionVisible( StatusBar.STATUS, true );    _tb.setSectionVisible( ToolBar.ANIMATION, false );    _tb.setSectionVisible( ToolBar.EXECUTION, true );    _tb.setSectionVisible( ToolBar.FILE, false );    _tb.setSectionEnabled( ToolBar.NAVIGATION, false );    _tb.setSectionVisible( ToolBar.NAVIGATION, false );    _tb.setSectionEnabled( ToolBar.TIME_SCALE, false );    _tb.setSectionVisible( ToolBar.TIME_SCALE, false );    _tb.setSectionVisible( ToolBar.VIEW, false );  }    @Override  @AnyLogicInternalCodegenAPI  public Main createRoot( Engine engine ) {    // Create the root object    return new Main( engine, null, null );  }    @Override  @AnyLogicInternalCodegenAPI  public void onBeforeSimulationRun(Main root) {    male = root.male;    distance = root.distance;    educPrimaryComplete = root.educPrimaryComplete;    educPrimary = root.educPrimary;    age50 = root.age50;    age2249 = root.age2249;    age1521 = root.age1521;    age514 = root.age514;    q = root.q;    intercept = root.intercept;    educSecondaryComplete = root.educSecondaryComplete;    educSecondary = root.educSecondary;    logIncomeSq = root.logIncomeSq;    logIncome = root.logIncome;    maleSE = root.maleSE;    distanceSE = root.distanceSE;    educPrimaryCompleteSE = root.educPrimaryCompleteSE;    educPrimarySE = root.educPrimarySE;    age50SE = root.age50SE;    age2249SE = root.age2249SE;    age1521SE = root.age1521SE;    age514SE = root.age514SE;    qSE = root.qSE;    interceptSE = root.interceptSE;    educSecondaryCompleteSE = root.educSecondaryCompleteSE;    educSecondarySE = root.educSecondarySE;    logIncomeSqSE = root.logIncomeSqSE;    logIncomeSE = root.logIncomeSE;    iv = root.iv;    ivSE = root.ivSE;    phi = root.phi;    voucher = root.voucher;  }    @Override  @AnyLogicInternalCodegenAPI  public void onEngineFinished() {    final Main root = (Main) getEngine().getRoot();    // After simulation run code    paramVar.setCellValue(getCurrentIteration(), "Sheet1", ((getCurrentIteration()-1)*10 + getCurrentReplication()+1), 1);paramVar.setCellValue(voucher, "Sheet1", ((getCurrentIteration()-1)*10 + getCurrentReplication()+1), 2);paramVar.setCellValue(root.nNoCare, "Sheet1", ((getCurrentIteration()-1)*10 + getCurrentReplication()+1), 3);   }    @Override  @AnyLogicInternalCodegenAPI  public void onAfterExperiment() {    // After experiment code        paramVar.writeFile(true);   }    @Override  @AnyLogicInternalCodegenAPI  public void reset() {    // Replications setup    setUseReplications( false );  }    @Override  public int getRangeParametersNumber() {    return 1;  }    @AnyLogicInternalCodegenAPI  private double _voucher_From() {    double _value;    _value = 0 ;    return _value;  }  @AnyLogicInternalCodegenAPI  private double _voucher_To() {    double _value;    _value = 10000000 ;    return _value;  }  @AnyLogicInternalCodegenAPI  private double _voucher_Step() {    double _value;    _value = 10000 ;    return _value;  }    @Override  @AnyLogicInternalCodegenAPI  public int[] calculateRangeParameterValuesNumbers() {    int[] res = new int[1];    res[0] = (int) ((_voucher_To() - _voucher_From()) / _voucher_Step() + 1);    return res;  }    @Override  @AnyLogicInternalCodegenAPI  public void setupRangeVariedParameter(Main root, int paramIndex, int valueIndex, boolean callOnChangeActions) {    switch (paramIndex) {      case 0 :        double voucher_xjal = _voucher_From() + valueIndex * _voucher_Step();        if (callOnChangeActions) {          root.set_voucher( voucher_xjal );        } else {          root.voucher = voucher_xjal;        }        break;      default:        super.setupRangeVariedParameter(root, paramIndex, valueIndex, callOnChangeActions);        break;    }  }    @Override  @AnyLogicInternalCodegenAPI  public void setupRootParameters( final Main self, int index, boolean callOnChangeActions ) {    final Main root = self; // for compatibility    super.setupRootParameters( self, index, callOnChangeActions );    double age1521_xjal;    age1521_xjal = self._age1521_DefaultValue_xjal();    if (callOnChangeActions) {      self.set_age1521( age1521_xjal );    } else {      self.age1521 = age1521_xjal;    }    double age1521SE_xjal;    age1521SE_xjal = self._age1521SE_DefaultValue_xjal();    if (callOnChangeActions) {      self.set_age1521SE( age1521SE_xjal );    } else {      self.age1521SE = age1521SE_xjal;    }    double age2249_xjal;    age2249_xjal = self._age2249_DefaultValue_xjal();    if (callOnChangeActions) {      self.set_age2249( age2249_xjal );    } else {      self.age2249 = age2249_xjal;    }    double age2249SE_xjal;    age2249SE_xjal = self._age2249SE_DefaultValue_xjal();    if (callOnChangeActions) {      self.set_age2249SE( age2249SE_xjal );    } else {      self.age2249SE = age2249SE_xjal;    }    double age50_xjal;    age50_xjal = self._age50_DefaultValue_xjal();    if (callOnChangeActions) {      self.set_age50( age50_xjal );    } else {      self.age50 = age50_xjal;    }    double age50SE_xjal;    age50SE_xjal = self._age50SE_DefaultValue_xjal();    if (callOnChangeActions) {      self.set_age50SE( age50SE_xjal );    } else {      self.age50SE = age50SE_xjal;    }    double age514_xjal;    age514_xjal = self._age514_DefaultValue_xjal();    if (callOnChangeActions) {      self.set_age514( age514_xjal );    } else {      self.age514 = age514_xjal;    }    double age514SE_xjal;    age514SE_xjal = self._age514SE_DefaultValue_xjal();    if (callOnChangeActions) {      self.set_age514SE( age514SE_xjal );    } else {      self.age514SE = age514SE_xjal;    }    double distance_xjal;    distance_xjal = self._distance_DefaultValue_xjal();    if (callOnChangeActions) {      self.set_distance( distance_xjal );    } else {      self.distance = distance_xjal;    }    double distanceSE_xjal;    distanceSE_xjal = self._distanceSE_DefaultValue_xjal();    if (callOnChangeActions) {      self.set_distanceSE( distanceSE_xjal );    } else {      self.distanceSE = distanceSE_xjal;    }    double educPrimary_xjal;    educPrimary_xjal = self._educPrimary_DefaultValue_xjal();    if (callOnChangeActions) {      self.set_educPrimary( educPrimary_xjal );    } else {      self.educPrimary = educPrimary_xjal;    }    double educPrimaryComplete_xjal;    educPrimaryComplete_xjal = self._educPrimaryComplete_DefaultValue_xjal();    if (callOnChangeActions) {      self.set_educPrimaryComplete( educPrimaryComplete_xjal );    } else {      self.educPrimaryComplete = educPrimaryComplete_xjal;    }    double educPrimaryCompleteSE_xjal;    educPrimaryCompleteSE_xjal = self._educPrimaryCompleteSE_DefaultValue_xjal();    if (callOnChangeActions) {      self.set_educPrimaryCompleteSE( educPrimaryCompleteSE_xjal );    } else {      self.educPrimaryCompleteSE = educPrimaryCompleteSE_xjal;    }    double educPrimarySE_xjal;    educPrimarySE_xjal = self._educPrimarySE_DefaultValue_xjal();    if (callOnChangeActions) {      self.set_educPrimarySE( educPrimarySE_xjal );    } else {      self.educPrimarySE = educPrimarySE_xjal;    }    double educSecondary_xjal;    educSecondary_xjal = self._educSecondary_DefaultValue_xjal();    if (callOnChangeActions) {      self.set_educSecondary( educSecondary_xjal );    } else {      self.educSecondary = educSecondary_xjal;    }    double educSecondaryComplete_xjal;    educSecondaryComplete_xjal = self._educSecondaryComplete_DefaultValue_xjal();    if (callOnChangeActions) {      self.set_educSecondaryComplete( educSecondaryComplete_xjal );    } else {      self.educSecondaryComplete = educSecondaryComplete_xjal;    }    double educSecondaryCompleteSE_xjal;    educSecondaryCompleteSE_xjal = self._educSecondaryCompleteSE_DefaultValue_xjal();    if (callOnChangeActions) {      self.set_educSecondaryCompleteSE( educSecondaryCompleteSE_xjal );    } else {      self.educSecondaryCompleteSE = educSecondaryCompleteSE_xjal;    }    double educSecondarySE_xjal;    educSecondarySE_xjal = self._educSecondarySE_DefaultValue_xjal();    if (callOnChangeActions) {      self.set_educSecondarySE( educSecondarySE_xjal );    } else {      self.educSecondarySE = educSecondarySE_xjal;    }    double intercept_xjal;    intercept_xjal = self._intercept_DefaultValue_xjal();    if (callOnChangeActions) {      self.set_intercept( intercept_xjal );    } else {      self.intercept = intercept_xjal;    }    double interceptSE_xjal;    interceptSE_xjal = self._interceptSE_DefaultValue_xjal();    if (callOnChangeActions) {      self.set_interceptSE( interceptSE_xjal );    } else {      self.interceptSE = interceptSE_xjal;    }    double iv_xjal;    iv_xjal = self._iv_DefaultValue_xjal();    if (callOnChangeActions) {      self.set_iv( iv_xjal );    } else {      self.iv = iv_xjal;    }    double ivSE_xjal;    ivSE_xjal = self._ivSE_DefaultValue_xjal();    if (callOnChangeActions) {      self.set_ivSE( ivSE_xjal );    } else {      self.ivSE = ivSE_xjal;    }    double logIncome_xjal;    logIncome_xjal = self._logIncome_DefaultValue_xjal();    if (callOnChangeActions) {      self.set_logIncome( logIncome_xjal );    } else {      self.logIncome = logIncome_xjal;    }    double logIncomeSE_xjal;    logIncomeSE_xjal = self._logIncomeSE_DefaultValue_xjal();    if (callOnChangeActions) {      self.set_logIncomeSE( logIncomeSE_xjal );    } else {      self.logIncomeSE = logIncomeSE_xjal;    }    double logIncomeSq_xjal;    logIncomeSq_xjal = self._logIncomeSq_DefaultValue_xjal();    if (callOnChangeActions) {      self.set_logIncomeSq( logIncomeSq_xjal );    } else {      self.logIncomeSq = logIncomeSq_xjal;    }    double logIncomeSqSE_xjal;    logIncomeSqSE_xjal = self._logIncomeSqSE_DefaultValue_xjal();    if (callOnChangeActions) {      self.set_logIncomeSqSE( logIncomeSqSE_xjal );    } else {      self.logIncomeSqSE = logIncomeSqSE_xjal;    }    double male_xjal;    male_xjal = self._male_DefaultValue_xjal();    if (callOnChangeActions) {      self.set_male( male_xjal );    } else {      self.male = male_xjal;    }    double maleSE_xjal;    maleSE_xjal = self._maleSE_DefaultValue_xjal();    if (callOnChangeActions) {      self.set_maleSE( maleSE_xjal );    } else {      self.maleSE = maleSE_xjal;    }    double phi_xjal;    phi_xjal = self._phi_DefaultValue_xjal();    if (callOnChangeActions) {      self.set_phi( phi_xjal );    } else {      self.phi = phi_xjal;    }    double q_xjal;    q_xjal = self._q_DefaultValue_xjal();    if (callOnChangeActions) {      self.set_q( q_xjal );    } else {      self.q = q_xjal;    }    double qSE_xjal;    qSE_xjal = self._qSE_DefaultValue_xjal();    if (callOnChangeActions) {      self.set_qSE( qSE_xjal );    } else {      self.qSE = qSE_xjal;    }  }  }package malawi_recentralization_model_2017_06_04;import java.io.Serializable;import java.sql.Connection;import java.sql.SQLException;import java.util.ArrayDeque;import java.util.ArrayList;import java.util.Arrays;import java.util.Calendar;import java.util.Collection;import java.util.Collections;import java.util.Comparator;import java.util.Currency;import java.util.Date;import java.util.Enumeration;import java.util.HashMap;import java.util.HashSet;import java.util.Hashtable;import java.util.Iterator;import java.util.LinkedHashMap;import java.util.LinkedHashSet;import java.util.LinkedList;import java.util.List;import java.util.ListIterator;import java.util.Locale;import java.util.Map;import java.util.PriorityQueue;import java.util.Random;import java.util.Set;import java.util.SortedMap;import java.util.SortedSet;import java.util.Stack;import java.util.Timer;import java.util.TreeMap;import java.util.TreeSet;import java.util.Vector;import java.awt.Color;import java.awt.Font;import java.awt.Graphics2D;import java.awt.geom.AffineTransform;import com.anylogic.engine.connectivity.ResultSet;import com.anylogic.engine.connectivity.Statement;import com.anylogic.engine.elements.*;import com.anylogic.engine.markup.Network;import com.anylogic.engine.Position;import com.anylogic.engine.markup.PedFlowStatistics;import com.anylogic.engine.markup.DensityMap;import static java.lang.Math.*;import static com.anylogic.engine.UtilitiesArray.*;import static com.anylogic.engine.UtilitiesCollection.*;import static com.anylogic.engine.presentation.UtilitiesColor.*;import static com.anylogic.engine.HyperArray.*;import com.anylogic.engine.*;import com.anylogic.engine.analysis.*;import com.anylogic.engine.connectivity.*;import com.anylogic.engine.database.*;import com.anylogic.engine.gis.*;import com.anylogic.engine.markup.*;import com.anylogic.engine.presentation.*;import com.mysema.query.Tuple;import com.mysema.query.sql.SQLBindings;import static malawi_recentralization_model_2017_06_04.DBDescriptor.*;import java.awt.geom.Arc2D;import java.sql.Time;import org.uncommons.maths.random.MersenneTwisterRNG;import org.uncommons.maths.random.GaussianGenerator; public class Main extends Agent{  // Parameters  public double  male;  /**   * Returns default value for parameter <code>male</code>.   * <i>This method should not be called by user</i>   */  @AnyLogicInternalCodegenAPI  public double _male_DefaultValue_xjal() {    final Main self = this;    return (double) selectFrom(parameters)	.where(parameters.param.eq("Male"))	.uniqueResult(parameters.value) ;  }  public void set_male( double male ) {    if (male == this.male) {      return;    }    double _oldValue_xjal = this.male;    this.male = male;    onChange_male_xjal( _oldValue_xjal );    onChange();  }  /**   * Calls "On change" action for parameter male.<br>   * Note that 'oldValue' in that action will be unavailable if this method is called by user   * (current parameter value will be passed as 'oldValue').<br>   * Please call <code>set_male()</code> method instead.   */  protected void onChange_male() {    onChange_male_xjal( male );  }  @AnyLogicInternalCodegenAPI  protected void onChange_male_xjal( double oldValue ) {    }  public double  distance;  /**   * Returns default value for parameter <code>distance</code>.   * <i>This method should not be called by user</i>   */  @AnyLogicInternalCodegenAPI  public double _distance_DefaultValue_xjal() {    final Main self = this;    return (double) selectFrom(parameters)	.where(parameters.param.eq("Distance"))	.uniqueResult(parameters.value) ;  }  public void set_distance( double distance ) {    if (distance == this.distance) {      return;    }    double _oldValue_xjal = this.distance;    this.distance = distance;    onChange_distance_xjal( _oldValue_xjal );    onChange();  }  /**   * Calls "On change" action for parameter distance.<br>   * Note that 'oldValue' in that action will be unavailable if this method is called by user   * (current parameter value will be passed as 'oldValue').<br>   * Please call <code>set_distance()</code> method instead.   */  protected void onChange_distance() {    onChange_distance_xjal( distance );  }  @AnyLogicInternalCodegenAPI  protected void onChange_distance_xjal( double oldValue ) {    }  public double  educPrimaryComplete;  /**   * Returns default value for parameter <code>educPrimaryComplete</code>.   * <i>This method should not be called by user</i>   */  @AnyLogicInternalCodegenAPI  public double _educPrimaryComplete_DefaultValue_xjal() {    final Main self = this;    return (double) selectFrom(parameters)	.where(parameters.param.eq("EducPrimaryComplete"))	.uniqueResult(parameters.value) ;  }  public void set_educPrimaryComplete( double educPrimaryComplete ) {    if (educPrimaryComplete == this.educPrimaryComplete) {      return;    }    double _oldValue_xjal = this.educPrimaryComplete;    this.educPrimaryComplete = educPrimaryComplete;    onChange_educPrimaryComplete_xjal( _oldValue_xjal );    onChange();  }  /**   * Calls "On change" action for parameter educPrimaryComplete.<br>   * Note that 'oldValue' in that action will be unavailable if this method is called by user   * (current parameter value will be passed as 'oldValue').<br>   * Please call <code>set_educPrimaryComplete()</code> method instead.   */  protected void onChange_educPrimaryComplete() {    onChange_educPrimaryComplete_xjal( educPrimaryComplete );  }  @AnyLogicInternalCodegenAPI  protected void onChange_educPrimaryComplete_xjal( double oldValue ) {    }  public double  educPrimary;  /**   * Returns default value for parameter <code>educPrimary</code>.   * <i>This method should not be called by user</i>   */  @AnyLogicInternalCodegenAPI  public double _educPrimary_DefaultValue_xjal() {    final Main self = this;    return (double) selectFrom(parameters)	.where(parameters.param.eq("EducPrimary"))	.uniqueResult(parameters.value) ;  }  public void set_educPrimary( double educPrimary ) {    if (educPrimary == this.educPrimary) {      return;    }    double _oldValue_xjal = this.educPrimary;    this.educPrimary = educPrimary;    onChange_educPrimary_xjal( _oldValue_xjal );    onChange();  }  /**   * Calls "On change" action for parameter educPrimary.<br>   * Note that 'oldValue' in that action will be unavailable if this method is called by user   * (current parameter value will be passed as 'oldValue').<br>   * Please call <code>set_educPrimary()</code> method instead.   */  protected void onChange_educPrimary() {    onChange_educPrimary_xjal( educPrimary );  }  @AnyLogicInternalCodegenAPI  protected void onChange_educPrimary_xjal( double oldValue ) {    }  public double  age50;  /**   * Returns default value for parameter <code>age50</code>.   * <i>This method should not be called by user</i>   */  @AnyLogicInternalCodegenAPI  public double _age50_DefaultValue_xjal() {    final Main self = this;    return (double) selectFrom(parameters)	.where(parameters.param.eq("Age50"))	.uniqueResult(parameters.value) ;  }  public void set_age50( double age50 ) {    if (age50 == this.age50) {      return;    }    double _oldValue_xjal = this.age50;    this.age50 = age50;    onChange_age50_xjal( _oldValue_xjal );    onChange();  }  /**   * Calls "On change" action for parameter age50.<br>   * Note that 'oldValue' in that action will be unavailable if this method is called by user   * (current parameter value will be passed as 'oldValue').<br>   * Please call <code>set_age50()</code> method instead.   */  protected void onChange_age50() {    onChange_age50_xjal( age50 );  }  @AnyLogicInternalCodegenAPI  protected void onChange_age50_xjal( double oldValue ) {    }  public double  age2249;  /**   * Returns default value for parameter <code>age2249</code>.   * <i>This method should not be called by user</i>   */  @AnyLogicInternalCodegenAPI  public double _age2249_DefaultValue_xjal() {    final Main self = this;    return (double) selectFrom(parameters)	.where(parameters.param.eq("Age2249"))	.uniqueResult(parameters.value) ;  }  public void set_age2249( double age2249 ) {    if (age2249 == this.age2249) {      return;    }    double _oldValue_xjal = this.age2249;    this.age2249 = age2249;    onChange_age2249_xjal( _oldValue_xjal );    onChange();  }  /**   * Calls "On change" action for parameter age2249.<br>   * Note that 'oldValue' in that action will be unavailable if this method is called by user   * (current parameter value will be passed as 'oldValue').<br>   * Please call <code>set_age2249()</code> method instead.   */  protected void onChange_age2249() {    onChange_age2249_xjal( age2249 );  }  @AnyLogicInternalCodegenAPI  protected void onChange_age2249_xjal( double oldValue ) {    }  public double  age1521;  /**   * Returns default value for parameter <code>age1521</code>.   * <i>This method should not be called by user</i>   */  @AnyLogicInternalCodegenAPI  public double _age1521_DefaultValue_xjal() {    final Main self = this;    return (double) selectFrom(parameters)	.where(parameters.param.eq("Age1521"))	.uniqueResult(parameters.value) ;  }  public void set_age1521( double age1521 ) {    if (age1521 == this.age1521) {      return;    }    double _oldValue_xjal = this.age1521;    this.age1521 = age1521;    onChange_age1521_xjal( _oldValue_xjal );    onChange();  }  /**   * Calls "On change" action for parameter age1521.<br>   * Note that 'oldValue' in that action will be unavailable if this method is called by user   * (current parameter value will be passed as 'oldValue').<br>   * Please call <code>set_age1521()</code> method instead.   */  protected void onChange_age1521() {    onChange_age1521_xjal( age1521 );  }  @AnyLogicInternalCodegenAPI  protected void onChange_age1521_xjal( double oldValue ) {    }  public double  age514;  /**   * Returns default value for parameter <code>age514</code>.   * <i>This method should not be called by user</i>   */  @AnyLogicInternalCodegenAPI  public double _age514_DefaultValue_xjal() {    final Main self = this;    return (double) selectFrom(parameters)	.where(parameters.param.eq("Age514"))	.uniqueResult(parameters.value) ;  }  public void set_age514( double age514 ) {    if (age514 == this.age514) {      return;    }    double _oldValue_xjal = this.age514;    this.age514 = age514;    onChange_age514_xjal( _oldValue_xjal );    onChange();  }  /**   * Calls "On change" action for parameter age514.<br>   * Note that 'oldValue' in that action will be unavailable if this method is called by user   * (current parameter value will be passed as 'oldValue').<br>   * Please call <code>set_age514()</code> method instead.   */  protected void onChange_age514() {    onChange_age514_xjal( age514 );  }  @AnyLogicInternalCodegenAPI  protected void onChange_age514_xjal( double oldValue ) {    }  public double  q;  /**   * Returns default value for parameter <code>q</code>.   * <i>This method should not be called by user</i>   */  @AnyLogicInternalCodegenAPI  public double _q_DefaultValue_xjal() {    final Main self = this;    return (double) selectFrom(parameters)	.where(parameters.param.eq("Q"))	.uniqueResult(parameters.value) ;  }  public void set_q( double q ) {    if (q == this.q) {      return;    }    double _oldValue_xjal = this.q;    this.q = q;    onChange_q_xjal( _oldValue_xjal );    onChange();  }  /**   * Calls "On change" action for parameter q.<br>   * Note that 'oldValue' in that action will be unavailable if this method is called by user   * (current parameter value will be passed as 'oldValue').<br>   * Please call <code>set_q()</code> method instead.   */  protected void onChange_q() {    onChange_q_xjal( q );  }  @AnyLogicInternalCodegenAPI  protected void onChange_q_xjal( double oldValue ) {    }  public double  intercept;  /**   * Returns default value for parameter <code>intercept</code>.   * <i>This method should not be called by user</i>   */  @AnyLogicInternalCodegenAPI  public double _intercept_DefaultValue_xjal() {    final Main self = this;    return (double) selectFrom(parameters)	.where(parameters.param.eq("Constant"))	.uniqueResult(parameters.value) ;  }  public void set_intercept( double intercept ) {    if (intercept == this.intercept) {      return;    }    double _oldValue_xjal = this.intercept;    this.intercept = intercept;    onChange_intercept_xjal( _oldValue_xjal );    onChange();  }  /**   * Calls "On change" action for parameter intercept.<br>   * Note that 'oldValue' in that action will be unavailable if this method is called by user   * (current parameter value will be passed as 'oldValue').<br>   * Please call <code>set_intercept()</code> method instead.   */  protected void onChange_intercept() {    onChange_intercept_xjal( intercept );  }  @AnyLogicInternalCodegenAPI  protected void onChange_intercept_xjal( double oldValue ) {    }  public double  educSecondaryComplete;  /**   * Returns default value for parameter <code>educSecondaryComplete</code>.   * <i>This method should not be called by user</i>   */  @AnyLogicInternalCodegenAPI  public double _educSecondaryComplete_DefaultValue_xjal() {    final Main self = this;    return (double) selectFrom(parameters)	.where(parameters.param.eq("EducSecondaryComplete"))	.uniqueResult(parameters.value) ;  }  public void set_educSecondaryComplete( double educSecondaryComplete ) {    if (educSecondaryComplete == this.educSecondaryComplete) {      return;    }    double _oldValue_xjal = this.educSecondaryComplete;    this.educSecondaryComplete = educSecondaryComplete;    onChange_educSecondaryComplete_xjal( _oldValue_xjal );    onChange();  }  /**   * Calls "On change" action for parameter educSecondaryComplete.<br>   * Note that 'oldValue' in that action will be unavailable if this method is called by user   * (current parameter value will be passed as 'oldValue').<br>   * Please call <code>set_educSecondaryComplete()</code> method instead.   */  protected void onChange_educSecondaryComplete() {    onChange_educSecondaryComplete_xjal( educSecondaryComplete );  }  @AnyLogicInternalCodegenAPI  protected void onChange_educSecondaryComplete_xjal( double oldValue ) {    }  public double  educSecondary;  /**   * Returns default value for parameter <code>educSecondary</code>.   * <i>This method should not be called by user</i>   */  @AnyLogicInternalCodegenAPI  public double _educSecondary_DefaultValue_xjal() {    final Main self = this;    return (double) selectFrom(parameters)	.where(parameters.param.eq("EducSecondary"))	.uniqueResult(parameters.value) ;  }  public void set_educSecondary( double educSecondary ) {    if (educSecondary == this.educSecondary) {      return;    }    double _oldValue_xjal = this.educSecondary;    this.educSecondary = educSecondary;    onChange_educSecondary_xjal( _oldValue_xjal );    onChange();  }  /**   * Calls "On change" action for parameter educSecondary.<br>   * Note that 'oldValue' in that action will be unavailable if this method is called by user   * (current parameter value will be passed as 'oldValue').<br>   * Please call <code>set_educSecondary()</code> method instead.   */  protected void onChange_educSecondary() {    onChange_educSecondary_xjal( educSecondary );  }  @AnyLogicInternalCodegenAPI  protected void onChange_educSecondary_xjal( double oldValue ) {    }  public double  logIncomeSq;  /**   * Returns default value for parameter <code>logIncomeSq</code>.   * <i>This method should not be called by user</i>   */  @AnyLogicInternalCodegenAPI  public double _logIncomeSq_DefaultValue_xjal() {    final Main self = this;    return (double) selectFrom(parameters)	.where(parameters.param.eq("LogIncomeSq"))	.uniqueResult(parameters.value) ;  }  public void set_logIncomeSq( double logIncomeSq ) {    if (logIncomeSq == this.logIncomeSq) {      return;    }    double _oldValue_xjal = this.logIncomeSq;    this.logIncomeSq = logIncomeSq;    onChange_logIncomeSq_xjal( _oldValue_xjal );    onChange();  }  /**   * Calls "On change" action for parameter logIncomeSq.<br>   * Note that 'oldValue' in that action will be unavailable if this method is called by user   * (current parameter value will be passed as 'oldValue').<br>   * Please call <code>set_logIncomeSq()</code> method instead.   */  protected void onChange_logIncomeSq() {    onChange_logIncomeSq_xjal( logIncomeSq );  }  @AnyLogicInternalCodegenAPI  protected void onChange_logIncomeSq_xjal( double oldValue ) {    }  public double  logIncome;  /**   * Returns default value for parameter <code>logIncome</code>.   * <i>This method should not be called by user</i>   */  @AnyLogicInternalCodegenAPI  public double _logIncome_DefaultValue_xjal() {    final Main self = this;    return (double) selectFrom(parameters)	.where(parameters.param.eq("LogIncome"))	.uniqueResult(parameters.value) ;  }  public void set_logIncome( double logIncome ) {    if (logIncome == this.logIncome) {      return;    }    double _oldValue_xjal = this.logIncome;    this.logIncome = logIncome;    onChange_logIncome_xjal( _oldValue_xjal );    onChange();  }  /**   * Calls "On change" action for parameter logIncome.<br>   * Note that 'oldValue' in that action will be unavailable if this method is called by user   * (current parameter value will be passed as 'oldValue').<br>   * Please call <code>set_logIncome()</code> method instead.   */  protected void onChange_logIncome() {    onChange_logIncome_xjal( logIncome );  }  @AnyLogicInternalCodegenAPI  protected void onChange_logIncome_xjal( double oldValue ) {    }  public double  maleSE;  /**   * Returns default value for parameter <code>maleSE</code>.   * <i>This method should not be called by user</i>   */  @AnyLogicInternalCodegenAPI  public double _maleSE_DefaultValue_xjal() {    final Main self = this;    return (double) selectFrom(parameters)	.where(parameters.param.eq("Male"))	.uniqueResult(parameters.sd) ;  }  public void set_maleSE( double maleSE ) {    if (maleSE == this.maleSE) {      return;    }    double _oldValue_xjal = this.maleSE;    this.maleSE = maleSE;    onChange_maleSE_xjal( _oldValue_xjal );    onChange();  }  /**   * Calls "On change" action for parameter maleSE.<br>   * Note that 'oldValue' in that action will be unavailable if this method is called by user   * (current parameter value will be passed as 'oldValue').<br>   * Please call <code>set_maleSE()</code> method instead.   */  protected void onChange_maleSE() {    onChange_maleSE_xjal( maleSE );  }  @AnyLogicInternalCodegenAPI  protected void onChange_maleSE_xjal( double oldValue ) {    }  public double  distanceSE;  /**   * Returns default value for parameter <code>distanceSE</code>.   * <i>This method should not be called by user</i>   */  @AnyLogicInternalCodegenAPI  public double _distanceSE_DefaultValue_xjal() {    final Main self = this;    return (double) selectFrom(parameters)	.where(parameters.param.eq("Distance"))	.uniqueResult(parameters.sd) ;  }  public void set_distanceSE( double distanceSE ) {    if (distanceSE == this.distanceSE) {      return;    }    double _oldValue_xjal = this.distanceSE;    this.distanceSE = distanceSE;    onChange_distanceSE_xjal( _oldValue_xjal );    onChange();  }  /**   * Calls "On change" action for parameter distanceSE.<br>   * Note that 'oldValue' in that action will be unavailable if this method is called by user   * (current parameter value will be passed as 'oldValue').<br>   * Please call <code>set_distanceSE()</code> method instead.   */  protected void onChange_distanceSE() {    onChange_distanceSE_xjal( distanceSE );  }  @AnyLogicInternalCodegenAPI  protected void onChange_distanceSE_xjal( double oldValue ) {    }  public double  educPrimaryCompleteSE;  /**   * Returns default value for parameter <code>educPrimaryCompleteSE</code>.   * <i>This method should not be called by user</i>   */  @AnyLogicInternalCodegenAPI  public double _educPrimaryCompleteSE_DefaultValue_xjal() {    final Main self = this;    return (double) selectFrom(parameters)	.where(parameters.param.eq("EducPrimaryComplete"))	.uniqueResult(parameters.sd) ;  }  public void set_educPrimaryCompleteSE( double educPrimaryCompleteSE ) {    if (educPrimaryCompleteSE == this.educPrimaryCompleteSE) {      return;    }    double _oldValue_xjal = this.educPrimaryCompleteSE;    this.educPrimaryCompleteSE = educPrimaryCompleteSE;    onChange_educPrimaryCompleteSE_xjal( _oldValue_xjal );    onChange();  }  /**   * Calls "On change" action for parameter educPrimaryCompleteSE.<br>   * Note that 'oldValue' in that action will be unavailable if this method is called by user   * (current parameter value will be passed as 'oldValue').<br>   * Please call <code>set_educPrimaryCompleteSE()</code> method instead.   */  protected void onChange_educPrimaryCompleteSE() {    onChange_educPrimaryCompleteSE_xjal( educPrimaryCompleteSE );  }  @AnyLogicInternalCodegenAPI  protected void onChange_educPrimaryCompleteSE_xjal( double oldValue ) {    }  public double  educPrimarySE;  /**   * Returns default value for parameter <code>educPrimarySE</code>.   * <i>This method should not be called by user</i>   */  @AnyLogicInternalCodegenAPI  public double _educPrimarySE_DefaultValue_xjal() {    final Main self = this;    return (double) selectFrom(parameters)	.where(parameters.param.eq("EducPrimary"))	.uniqueResult(parameters.sd) ;  }  public void set_educPrimarySE( double educPrimarySE ) {    if (educPrimarySE == this.educPrimarySE) {      return;    }    double _oldValue_xjal = this.educPrimarySE;    this.educPrimarySE = educPrimarySE;    onChange_educPrimarySE_xjal( _oldValue_xjal );    onChange();  }  /**   * Calls "On change" action for parameter educPrimarySE.<br>   * Note that 'oldValue' in that action will be unavailable if this method is called by user   * (current parameter value will be passed as 'oldValue').<br>   * Please call <code>set_educPrimarySE()</code> method instead.   */  protected void onChange_educPrimarySE() {    onChange_educPrimarySE_xjal( educPrimarySE );  }  @AnyLogicInternalCodegenAPI  protected void onChange_educPrimarySE_xjal( double oldValue ) {    }  public double  age50SE;  /**   * Returns default value for parameter <code>age50SE</code>.   * <i>This method should not be called by user</i>   */  @AnyLogicInternalCodegenAPI  public double _age50SE_DefaultValue_xjal() {    final Main self = this;    return (double) selectFrom(parameters)	.where(parameters.param.eq("Age50"))	.uniqueResult(parameters.sd) ;  }  public void set_age50SE( double age50SE ) {    if (age50SE == this.age50SE) {      return;    }    double _oldValue_xjal = this.age50SE;    this.age50SE = age50SE;    onChange_age50SE_xjal( _oldValue_xjal );    onChange();  }  /**   * Calls "On change" action for parameter age50SE.<br>   * Note that 'oldValue' in that action will be unavailable if this method is called by user   * (current parameter value will be passed as 'oldValue').<br>   * Please call <code>set_age50SE()</code> method instead.   */  protected void onChange_age50SE() {    onChange_age50SE_xjal( age50SE );  }  @AnyLogicInternalCodegenAPI  protected void onChange_age50SE_xjal( double oldValue ) {    }  public double  age2249SE;  /**   * Returns default value for parameter <code>age2249SE</code>.   * <i>This method should not be called by user</i>   */  @AnyLogicInternalCodegenAPI  public double _age2249SE_DefaultValue_xjal() {    final Main self = this;    return (double) selectFrom(parameters)	.where(parameters.param.eq("Age2249"))	.uniqueResult(parameters.sd) ;  }  public void set_age2249SE( double age2249SE ) {    if (age2249SE == this.age2249SE) {      return;    }    double _oldValue_xjal = this.age2249SE;    this.age2249SE = age2249SE;    onChange_age2249SE_xjal( _oldValue_xjal );    onChange();  }  /**   * Calls "On change" action for parameter age2249SE.<br>   * Note that 'oldValue' in that action will be unavailable if this method is called by user   * (current parameter value will be passed as 'oldValue').<br>   * Please call <code>set_age2249SE()</code> method instead.   */  protected void onChange_age2249SE() {    onChange_age2249SE_xjal( age2249SE );  }  @AnyLogicInternalCodegenAPI  protected void onChange_age2249SE_xjal( double oldValue ) {    }  public double  age1521SE;  /**   * Returns default value for parameter <code>age1521SE</code>.   * <i>This method should not be called by user</i>   */  @AnyLogicInternalCodegenAPI  public double _age1521SE_DefaultValue_xjal() {    final Main self = this;    return (double) selectFrom(parameters)	.where(parameters.param.eq("Age1521"))	.uniqueResult(parameters.sd) ;  }  public void set_age1521SE( double age1521SE ) {    if (age1521SE == this.age1521SE) {      return;    }    double _oldValue_xjal = this.age1521SE;    this.age1521SE = age1521SE;    onChange_age1521SE_xjal( _oldValue_xjal );    onChange();  }  /**   * Calls "On change" action for parameter age1521SE.<br>   * Note that 'oldValue' in that action will be unavailable if this method is called by user   * (current parameter value will be passed as 'oldValue').<br>   * Please call <code>set_age1521SE()</code> method instead.   */  protected void onChange_age1521SE() {    onChange_age1521SE_xjal( age1521SE );  }  @AnyLogicInternalCodegenAPI  protected void onChange_age1521SE_xjal( double oldValue ) {    }  public double  age514SE;  /**   * Returns default value for parameter <code>age514SE</code>.   * <i>This method should not be called by user</i>   */  @AnyLogicInternalCodegenAPI  public double _age514SE_DefaultValue_xjal() {    final Main self = this;    return (double) selectFrom(parameters)	.where(parameters.param.eq("Age514"))	.uniqueResult(parameters.sd) ;  }  public void set_age514SE( double age514SE ) {    if (age514SE == this.age514SE) {      return;    }    double _oldValue_xjal = this.age514SE;    this.age514SE = age514SE;    onChange_age514SE_xjal( _oldValue_xjal );    onChange();  }  /**   * Calls "On change" action for parameter age514SE.<br>   * Note that 'oldValue' in that action will be unavailable if this method is called by user   * (current parameter value will be passed as 'oldValue').<br>   * Please call <code>set_age514SE()</code> method instead.   */  protected void onChange_age514SE() {    onChange_age514SE_xjal( age514SE );  }  @AnyLogicInternalCodegenAPI  protected void onChange_age514SE_xjal( double oldValue ) {    }  public double  qSE;  /**   * Returns default value for parameter <code>qSE</code>.   * <i>This method should not be called by user</i>   */  @AnyLogicInternalCodegenAPI  public double _qSE_DefaultValue_xjal() {    final Main self = this;    return (double) selectFrom(parameters)	.where(parameters.param.eq("Q"))	.uniqueResult(parameters.sd) ;  }  public void set_qSE( double qSE ) {    if (qSE == this.qSE) {      return;    }    double _oldValue_xjal = this.qSE;    this.qSE = qSE;    onChange_qSE_xjal( _oldValue_xjal );    onChange();  }  /**   * Calls "On change" action for parameter qSE.<br>   * Note that 'oldValue' in that action will be unavailable if this method is called by user   * (current parameter value will be passed as 'oldValue').<br>   * Please call <code>set_qSE()</code> method instead.   */  protected void onChange_qSE() {    onChange_qSE_xjal( qSE );  }  @AnyLogicInternalCodegenAPI  protected void onChange_qSE_xjal( double oldValue ) {    }  public double  interceptSE;  /**   * Returns default value for parameter <code>interceptSE</code>.   * <i>This method should not be called by user</i>   */  @AnyLogicInternalCodegenAPI  public double _interceptSE_DefaultValue_xjal() {    final Main self = this;    return (double) selectFrom(parameters)	.where(parameters.param.eq("Constant"))	.uniqueResult(parameters.sd) ;  }  public void set_interceptSE( double interceptSE ) {    if (interceptSE == this.interceptSE) {      return;    }    double _oldValue_xjal = this.interceptSE;    this.interceptSE = interceptSE;    onChange_interceptSE_xjal( _oldValue_xjal );    onChange();  }  /**   * Calls "On change" action for parameter interceptSE.<br>   * Note that 'oldValue' in that action will be unavailable if this method is called by user   * (current parameter value will be passed as 'oldValue').<br>   * Please call <code>set_interceptSE()</code> method instead.   */  protected void onChange_interceptSE() {    onChange_interceptSE_xjal( interceptSE );  }  @AnyLogicInternalCodegenAPI  protected void onChange_interceptSE_xjal( double oldValue ) {    }  public double  educSecondaryCompleteSE;  /**   * Returns default value for parameter <code>educSecondaryCompleteSE</code>.   * <i>This method should not be called by user</i>   */  @AnyLogicInternalCodegenAPI  public double _educSecondaryCompleteSE_DefaultValue_xjal() {    final Main self = this;    return (double) selectFrom(parameters)	.where(parameters.param.eq("EducSecondaryComplete"))	.uniqueResult(parameters.sd) ;  }  public void set_educSecondaryCompleteSE( double educSecondaryCompleteSE ) {    if (educSecondaryCompleteSE == this.educSecondaryCompleteSE) {      return;    }    double _oldValue_xjal = this.educSecondaryCompleteSE;    this.educSecondaryCompleteSE = educSecondaryCompleteSE;    onChange_educSecondaryCompleteSE_xjal( _oldValue_xjal );    onChange();  }  /**   * Calls "On change" action for parameter educSecondaryCompleteSE.<br>   * Note that 'oldValue' in that action will be unavailable if this method is called by user   * (current parameter value will be passed as 'oldValue').<br>   * Please call <code>set_educSecondaryCompleteSE()</code> method instead.   */  protected void onChange_educSecondaryCompleteSE() {    onChange_educSecondaryCompleteSE_xjal( educSecondaryCompleteSE );  }  @AnyLogicInternalCodegenAPI  protected void onChange_educSecondaryCompleteSE_xjal( double oldValue ) {    }  public double  educSecondarySE;  /**   * Returns default value for parameter <code>educSecondarySE</code>.   * <i>This method should not be called by user</i>   */  @AnyLogicInternalCodegenAPI  public double _educSecondarySE_DefaultValue_xjal() {    final Main self = this;    return (double) selectFrom(parameters)	.where(parameters.param.eq("EducSecondary"))	.uniqueResult(parameters.sd) ;  }  public void set_educSecondarySE( double educSecondarySE ) {    if (educSecondarySE == this.educSecondarySE) {      return;    }    double _oldValue_xjal = this.educSecondarySE;    this.educSecondarySE = educSecondarySE;    onChange_educSecondarySE_xjal( _oldValue_xjal );    onChange();  }  /**   * Calls "On change" action for parameter educSecondarySE.<br>   * Note that 'oldValue' in that action will be unavailable if this method is called by user   * (current parameter value will be passed as 'oldValue').<br>   * Please call <code>set_educSecondarySE()</code> method instead.   */  protected void onChange_educSecondarySE() {    onChange_educSecondarySE_xjal( educSecondarySE );  }  @AnyLogicInternalCodegenAPI  protected void onChange_educSecondarySE_xjal( double oldValue ) {    }  public double  logIncomeSqSE;  /**   * Returns default value for parameter <code>logIncomeSqSE</code>.   * <i>This method should not be called by user</i>   */  @AnyLogicInternalCodegenAPI  public double _logIncomeSqSE_DefaultValue_xjal() {    final Main self = this;    return (double) selectFrom(parameters)	.where(parameters.param.eq("LogIncomeSq"))	.uniqueResult(parameters.sd) ;  }  public void set_logIncomeSqSE( double logIncomeSqSE ) {    if (logIncomeSqSE == this.logIncomeSqSE) {      return;    }    double _oldValue_xjal = this.logIncomeSqSE;    this.logIncomeSqSE = logIncomeSqSE;    onChange_logIncomeSqSE_xjal( _oldValue_xjal );    onChange();  }  /**   * Calls "On change" action for parameter logIncomeSqSE.<br>   * Note that 'oldValue' in that action will be unavailable if this method is called by user   * (current parameter value will be passed as 'oldValue').<br>   * Please call <code>set_logIncomeSqSE()</code> method instead.   */  protected void onChange_logIncomeSqSE() {    onChange_logIncomeSqSE_xjal( logIncomeSqSE );  }  @AnyLogicInternalCodegenAPI  protected void onChange_logIncomeSqSE_xjal( double oldValue ) {    }  public double  logIncomeSE;  /**   * Returns default value for parameter <code>logIncomeSE</code>.   * <i>This method should not be called by user</i>   */  @AnyLogicInternalCodegenAPI  public double _logIncomeSE_DefaultValue_xjal() {    final Main self = this;    return (double) selectFrom(parameters)	.where(parameters.param.eq("LogIncome"))	.uniqueResult(parameters.sd) ;  }  public void set_logIncomeSE( double logIncomeSE ) {    if (logIncomeSE == this.logIncomeSE) {      return;    }    double _oldValue_xjal = this.logIncomeSE;    this.logIncomeSE = logIncomeSE;    onChange_logIncomeSE_xjal( _oldValue_xjal );    onChange();  }  /**   * Calls "On change" action for parameter logIncomeSE.<br>   * Note that 'oldValue' in that action will be unavailable if this method is called by user   * (current parameter value will be passed as 'oldValue').<br>   * Please call <code>set_logIncomeSE()</code> method instead.   */  protected void onChange_logIncomeSE() {    onChange_logIncomeSE_xjal( logIncomeSE );  }  @AnyLogicInternalCodegenAPI  protected void onChange_logIncomeSE_xjal( double oldValue ) {    }  public double  iv;  /**   * Returns default value for parameter <code>iv</code>.   * <i>This method should not be called by user</i>   */  @AnyLogicInternalCodegenAPI  public double _iv_DefaultValue_xjal() {    final Main self = this;    return (double) selectFrom(parameters)	.where(parameters.param.eq("IV"))	.uniqueResult(parameters.value) ;  }  public void set_iv( double iv ) {    if (iv == this.iv) {      return;    }    double _oldValue_xjal = this.iv;    this.iv = iv;    onChange_iv_xjal( _oldValue_xjal );    onChange();  }  /**   * Calls "On change" action for parameter iv.<br>   * Note that 'oldValue' in that action will be unavailable if this method is called by user   * (current parameter value will be passed as 'oldValue').<br>   * Please call <code>set_iv()</code> method instead.   */  protected void onChange_iv() {    onChange_iv_xjal( iv );  }  @AnyLogicInternalCodegenAPI  protected void onChange_iv_xjal( double oldValue ) {    }  public double  ivSE;  /**   * Returns default value for parameter <code>ivSE</code>.   * <i>This method should not be called by user</i>   */  @AnyLogicInternalCodegenAPI  public double _ivSE_DefaultValue_xjal() {    final Main self = this;    return (double) selectFrom(parameters)	.where(parameters.param.eq("IV"))	.uniqueResult(parameters.sd) ;  }  public void set_ivSE( double ivSE ) {    if (ivSE == this.ivSE) {      return;    }    double _oldValue_xjal = this.ivSE;    this.ivSE = ivSE;    onChange_ivSE_xjal( _oldValue_xjal );    onChange();  }  /**   * Calls "On change" action for parameter ivSE.<br>   * Note that 'oldValue' in that action will be unavailable if this method is called by user   * (current parameter value will be passed as 'oldValue').<br>   * Please call <code>set_ivSE()</code> method instead.   */  protected void onChange_ivSE() {    onChange_ivSE_xjal( ivSE );  }  @AnyLogicInternalCodegenAPI  protected void onChange_ivSE_xjal( double oldValue ) {    }  /**   * Shape for the gamma distribution   */  public double  phi;  /**   * Returns default value for parameter <code>phi</code>.   * <i>This method should not be called by user</i>   */  @AnyLogicInternalCodegenAPI  public double _phi_DefaultValue_xjal() {    final Main self = this;    return 2.561818 ;  }  public void set_phi( double phi ) {    if (phi == this.phi) {      return;    }    double _oldValue_xjal = this.phi;    this.phi = phi;    onChange_phi_xjal( _oldValue_xjal );    onChange();  }  /**   * Calls "On change" action for parameter phi.<br>   * Note that 'oldValue' in that action will be unavailable if this method is called by user   * (current parameter value will be passed as 'oldValue').<br>   * Please call <code>set_phi()</code> method instead.   */  protected void onChange_phi() {    onChange_phi_xjal( phi );  }  @AnyLogicInternalCodegenAPI  protected void onChange_phi_xjal( double oldValue ) {    }  public double  voucher;  /**   * Returns default value for parameter <code>voucher</code>.   * <i>This method should not be called by user</i>   */  @AnyLogicInternalCodegenAPI  public double _voucher_DefaultValue_xjal() {    final Main self = this;    return 508034.0 ;  }  public void set_voucher( double voucher ) {    if (voucher == this.voucher) {      return;    }    double _oldValue_xjal = this.voucher;    this.voucher = voucher;    onChange_voucher_xjal( _oldValue_xjal );    onChange();  }  /**   * Calls "On change" action for parameter voucher.<br>   * Note that 'oldValue' in that action will be unavailable if this method is called by user   * (current parameter value will be passed as 'oldValue').<br>   * Please call <code>set_voucher()</code> method instead.   */  protected void onChange_voucher() {    onChange_voucher_xjal( voucher );  }  @AnyLogicInternalCodegenAPI  protected void onChange_voucher_xjal( double oldValue ) {    }  @Override  public void setParametersToDefaultValues() {    super.setParametersToDefaultValues();    male = _male_DefaultValue_xjal();    distance = _distance_DefaultValue_xjal();    educPrimaryComplete = _educPrimaryComplete_DefaultValue_xjal();    educPrimary = _educPrimary_DefaultValue_xjal();    age50 = _age50_DefaultValue_xjal();    age2249 = _age2249_DefaultValue_xjal();    age1521 = _age1521_DefaultValue_xjal();    age514 = _age514_DefaultValue_xjal();    q = _q_DefaultValue_xjal();    intercept = _intercept_DefaultValue_xjal();    educSecondaryComplete = _educSecondaryComplete_DefaultValue_xjal();    educSecondary = _educSecondary_DefaultValue_xjal();    logIncomeSq = _logIncomeSq_DefaultValue_xjal();    logIncome = _logIncome_DefaultValue_xjal();    maleSE = _maleSE_DefaultValue_xjal();    distanceSE = _distanceSE_DefaultValue_xjal();    educPrimaryCompleteSE = _educPrimaryCompleteSE_DefaultValue_xjal();    educPrimarySE = _educPrimarySE_DefaultValue_xjal();    age50SE = _age50SE_DefaultValue_xjal();    age2249SE = _age2249SE_DefaultValue_xjal();    age1521SE = _age1521SE_DefaultValue_xjal();    age514SE = _age514SE_DefaultValue_xjal();    qSE = _qSE_DefaultValue_xjal();    interceptSE = _interceptSE_DefaultValue_xjal();    educSecondaryCompleteSE = _educSecondaryCompleteSE_DefaultValue_xjal();    educSecondarySE = _educSecondarySE_DefaultValue_xjal();    logIncomeSqSE = _logIncomeSqSE_DefaultValue_xjal();    logIncomeSE = _logIncomeSE_DefaultValue_xjal();    iv = _iv_DefaultValue_xjal();    ivSE = _ivSE_DefaultValue_xjal();    phi = _phi_DefaultValue_xjal();    voucher = _voucher_DefaultValue_xjal();  }  @Override  public boolean setParameter(String _name_xjal, Object _value_xjal, boolean _callOnChange_xjal) {    switch ( _name_xjal ) {    case "male":      if ( _callOnChange_xjal ) {        set_male( ((Number) _value_xjal).doubleValue() );      } else {        male = ((Number) _value_xjal).doubleValue();      }      return true;    case "distance":      if ( _callOnChange_xjal ) {        set_distance( ((Number) _value_xjal).doubleValue() );      } else {        distance = ((Number) _value_xjal).doubleValue();      }      return true;    case "educPrimaryComplete":      if ( _callOnChange_xjal ) {        set_educPrimaryComplete( ((Number) _value_xjal).doubleValue() );      } else {        educPrimaryComplete = ((Number) _value_xjal).doubleValue();      }      return true;    case "educPrimary":      if ( _callOnChange_xjal ) {        set_educPrimary( ((Number) _value_xjal).doubleValue() );      } else {        educPrimary = ((Number) _value_xjal).doubleValue();      }      return true;    case "age50":      if ( _callOnChange_xjal ) {        set_age50( ((Number) _value_xjal).doubleValue() );      } else {        age50 = ((Number) _value_xjal).doubleValue();      }      return true;    case "age2249":      if ( _callOnChange_xjal ) {        set_age2249( ((Number) _value_xjal).doubleValue() );      } else {        age2249 = ((Number) _value_xjal).doubleValue();      }      return true;    case "age1521":      if ( _callOnChange_xjal ) {        set_age1521( ((Number) _value_xjal).doubleValue() );      } else {        age1521 = ((Number) _value_xjal).doubleValue();      }      return true;    case "age514":      if ( _callOnChange_xjal ) {        set_age514( ((Number) _value_xjal).doubleValue() );      } else {        age514 = ((Number) _value_xjal).doubleValue();      }      return true;    case "q":      if ( _callOnChange_xjal ) {        set_q( ((Number) _value_xjal).doubleValue() );      } else {        q = ((Number) _value_xjal).doubleValue();      }      return true;    case "intercept":      if ( _callOnChange_xjal ) {        set_intercept( ((Number) _value_xjal).doubleValue() );      } else {        intercept = ((Number) _value_xjal).doubleValue();      }      return true;    case "educSecondaryComplete":      if ( _callOnChange_xjal ) {        set_educSecondaryComplete( ((Number) _value_xjal).doubleValue() );      } else {        educSecondaryComplete = ((Number) _value_xjal).doubleValue();      }      return true;    case "educSecondary":      if ( _callOnChange_xjal ) {        set_educSecondary( ((Number) _value_xjal).doubleValue() );      } else {        educSecondary = ((Number) _value_xjal).doubleValue();      }      return true;    case "logIncomeSq":      if ( _callOnChange_xjal ) {        set_logIncomeSq( ((Number) _value_xjal).doubleValue() );      } else {        logIncomeSq = ((Number) _value_xjal).doubleValue();      }      return true;    case "logIncome":      if ( _callOnChange_xjal ) {        set_logIncome( ((Number) _value_xjal).doubleValue() );      } else {        logIncome = ((Number) _value_xjal).doubleValue();      }      return true;    case "maleSE":      if ( _callOnChange_xjal ) {        set_maleSE( ((Number) _value_xjal).doubleValue() );      } else {        maleSE = ((Number) _value_xjal).doubleValue();      }      return true;    case "distanceSE":      if ( _callOnChange_xjal ) {        set_distanceSE( ((Number) _value_xjal).doubleValue() );      } else {        distanceSE = ((Number) _value_xjal).doubleValue();      }      return true;    case "educPrimaryCompleteSE":      if ( _callOnChange_xjal ) {        set_educPrimaryCompleteSE( ((Number) _value_xjal).doubleValue() );      } else {        educPrimaryCompleteSE = ((Number) _value_xjal).doubleValue();      }      return true;    case "educPrimarySE":      if ( _callOnChange_xjal ) {        set_educPrimarySE( ((Number) _value_xjal).doubleValue() );      } else {        educPrimarySE = ((Number) _value_xjal).doubleValue();      }      return true;    case "age50SE":      if ( _callOnChange_xjal ) {        set_age50SE( ((Number) _value_xjal).doubleValue() );      } else {        age50SE = ((Number) _value_xjal).doubleValue();      }      return true;    case "age2249SE":      if ( _callOnChange_xjal ) {        set_age2249SE( ((Number) _value_xjal).doubleValue() );      } else {        age2249SE = ((Number) _value_xjal).doubleValue();      }      return true;    case "age1521SE":      if ( _callOnChange_xjal ) {        set_age1521SE( ((Number) _value_xjal).doubleValue() );      } else {        age1521SE = ((Number) _value_xjal).doubleValue();      }      return true;    case "age514SE":      if ( _callOnChange_xjal ) {        set_age514SE( ((Number) _value_xjal).doubleValue() );      } else {        age514SE = ((Number) _value_xjal).doubleValue();      }      return true;    case "qSE":      if ( _callOnChange_xjal ) {        set_qSE( ((Number) _value_xjal).doubleValue() );      } else {        qSE = ((Number) _value_xjal).doubleValue();      }      return true;    case "interceptSE":      if ( _callOnChange_xjal ) {        set_interceptSE( ((Number) _value_xjal).doubleValue() );      } else {        interceptSE = ((Number) _value_xjal).doubleValue();      }      return true;    case "educSecondaryCompleteSE":      if ( _callOnChange_xjal ) {        set_educSecondaryCompleteSE( ((Number) _value_xjal).doubleValue() );      } else {        educSecondaryCompleteSE = ((Number) _value_xjal).doubleValue();      }      return true;    case "educSecondarySE":      if ( _callOnChange_xjal ) {        set_educSecondarySE( ((Number) _value_xjal).doubleValue() );      } else {        educSecondarySE = ((Number) _value_xjal).doubleValue();      }      return true;    case "logIncomeSqSE":      if ( _callOnChange_xjal ) {        set_logIncomeSqSE( ((Number) _value_xjal).doubleValue() );      } else {        logIncomeSqSE = ((Number) _value_xjal).doubleValue();      }      return true;    case "logIncomeSE":      if ( _callOnChange_xjal ) {        set_logIncomeSE( ((Number) _value_xjal).doubleValue() );      } else {        logIncomeSE = ((Number) _value_xjal).doubleValue();      }      return true;    case "iv":      if ( _callOnChange_xjal ) {        set_iv( ((Number) _value_xjal).doubleValue() );      } else {        iv = ((Number) _value_xjal).doubleValue();      }      return true;    case "ivSE":      if ( _callOnChange_xjal ) {        set_ivSE( ((Number) _value_xjal).doubleValue() );      } else {        ivSE = ((Number) _value_xjal).doubleValue();      }      return true;    case "phi":      if ( _callOnChange_xjal ) {        set_phi( ((Number) _value_xjal).doubleValue() );      } else {        phi = ((Number) _value_xjal).doubleValue();      }      return true;    case "voucher":      if ( _callOnChange_xjal ) {        set_voucher( ((Number) _value_xjal).doubleValue() );      } else {        voucher = ((Number) _value_xjal).doubleValue();      }      return true;    default:      return super.setParameter( _name_xjal, _value_xjal, _callOnChange_xjal );    }  }  @Override  public <T> T getParameter(String _name_xjal) {    Object _result_xjal;    switch ( _name_xjal ) {    case "male": _result_xjal = male; break;    case "distance": _result_xjal = distance; break;    case "educPrimaryComplete": _result_xjal = educPrimaryComplete; break;    case "educPrimary": _result_xjal = educPrimary; break;    case "age50": _result_xjal = age50; break;    case "age2249": _result_xjal = age2249; break;    case "age1521": _result_xjal = age1521; break;    case "age514": _result_xjal = age514; break;    case "q": _result_xjal = q; break;    case "intercept": _result_xjal = intercept; break;    case "educSecondaryComplete": _result_xjal = educSecondaryComplete; break;    case "educSecondary": _result_xjal = educSecondary; break;    case "logIncomeSq": _result_xjal = logIncomeSq; break;    case "logIncome": _result_xjal = logIncome; break;    case "maleSE": _result_xjal = maleSE; break;    case "distanceSE": _result_xjal = distanceSE; break;    case "educPrimaryCompleteSE": _result_xjal = educPrimaryCompleteSE; break;    case "educPrimarySE": _result_xjal = educPrimarySE; break;    case "age50SE": _result_xjal = age50SE; break;    case "age2249SE": _result_xjal = age2249SE; break;    case "age1521SE": _result_xjal = age1521SE; break;    case "age514SE": _result_xjal = age514SE; break;    case "qSE": _result_xjal = qSE; break;    case "interceptSE": _result_xjal = interceptSE; break;    case "educSecondaryCompleteSE": _result_xjal = educSecondaryCompleteSE; break;    case "educSecondarySE": _result_xjal = educSecondarySE; break;    case "logIncomeSqSE": _result_xjal = logIncomeSqSE; break;    case "logIncomeSE": _result_xjal = logIncomeSE; break;    case "iv": _result_xjal = iv; break;    case "ivSE": _result_xjal = ivSE; break;    case "phi": _result_xjal = phi; break;    case "voucher": _result_xjal = voucher; break;    default: _result_xjal = super.getParameter( _name_xjal ); break;    }    return (T) _result_xjal;  }  @AnyLogicInternalCodegenAPI  private static String[] _parameterNames_xjal;  @Override  public String[] getParameterNames() {    String[] result = _parameterNames_xjal;    if (result == null) {      List<String> list = new ArrayList<>( Arrays.asList( super.getParameterNames() ) );      list.add( "male" );      list.add( "distance" );      list.add( "educPrimaryComplete" );      list.add( "educPrimary" );      list.add( "age50" );      list.add( "age2249" );      list.add( "age1521" );      list.add( "age514" );      list.add( "q" );      list.add( "intercept" );      list.add( "educSecondaryComplete" );      list.add( "educSecondary" );      list.add( "logIncomeSq" );      list.add( "logIncome" );      list.add( "maleSE" );      list.add( "distanceSE" );      list.add( "educPrimaryCompleteSE" );      list.add( "educPrimarySE" );      list.add( "age50SE" );      list.add( "age2249SE" );      list.add( "age1521SE" );      list.add( "age514SE" );      list.add( "qSE" );      list.add( "interceptSE" );      list.add( "educSecondaryCompleteSE" );      list.add( "educSecondarySE" );      list.add( "logIncomeSqSE" );      list.add( "logIncomeSE" );      list.add( "iv" );      list.add( "ivSE" );      list.add( "phi" );      list.add( "voucher" );      result = list.toArray( new String[ list.size() ] );      _parameterNames_xjal = result;    }    return result;  }  // Plain Variables  public double  vAgentID;  // Collection Variables  public ArrayList <Hospital > hospitalList = new ArrayList<Hospital>();  @AnyLogicInternalCodegenAPI  private static Map<String, IElementDescriptor> elementDesciptors_xjal = createElementDescriptors( Main.class );    @AnyLogicInternalCodegenAPI  @Override  public Map<String, IElementDescriptor> getElementDesciptors() {    return elementDesciptors_xjal;  }	  // Embedded Objects  @AnyLogicInternalCodegenAPI  private static final AgentAnimationSettings _hospitals_animationSettings_xjal = new AgentAnimationSettings(1000L, 1000000000L);  @AnyLogicInternalCodegenAPI  private static final AgentAnimationSettings _people_animationSettings_xjal = new AgentAnimationSettings(1000L, 1000000000L);  public String getNameOf( Agent ao ) {    return super.getNameOf( ao );  }  public AgentAnimationSettings getAnimationSettingsOf( Agent ao ) {    return super.getAnimationSettingsOf( ao );  }  public class _hospitals_Population extends AgentArrayList<Hospital> {    _hospitals_Population( Agent owner ) {      super( owner );    }    @AnyLogicInternalCodegenAPI    public Hospital instantiateAgent( int index ) {    	return instantiate_hospitals_xjal( index );    }    @AnyLogicInternalCodegenAPI    public void callSetupParameters( Hospital agent, int index, TableInput tableInput ) {      setupParameters_hospitals_xjal( agent, index, tableInput );    }    @AnyLogicInternalCodegenAPI    public void callCreate( Hospital agent, int index, TableInput tableInput ) {      create_hospitals_xjal( agent, index, tableInput );    }    @AnyLogicInternalCodegenAPI    public boolean isPresentationEnabled() {      return true;    }    public int vTooBig() {      return _hospitals_vTooBig_xjal();    }    public double avgCost() {      return _hospitals_avgCost_xjal();    }    public double avgQ() {      return _hospitals_avgQ_xjal();    }    public double costMinusVoucher() {      return _hospitals_costMinusVoucher_xjal();    }  }    @AnyLogicCustomProposalType(value = AnyLogicCustomProposalType.Label.POPULATION, customText = "Hospital")  public _hospitals_Population hospitals = new _hospitals_Population( this );  public class _people_Population extends AgentArrayList<Person> {    _people_Population( Agent owner ) {      super( owner );    }    @AnyLogicInternalCodegenAPI    public Person instantiateAgent( int index ) {    	return instantiate_people_xjal( index );    }    @AnyLogicInternalCodegenAPI    public void callSetupParameters( Person agent, int index, TableInput tableInput ) {      setupParameters_people_xjal( agent, index, tableInput );    }    @AnyLogicInternalCodegenAPI    public void callCreate( Person agent, int index, TableInput tableInput ) {      create_people_xjal( agent, index, tableInput );    }    @AnyLogicInternalCodegenAPI    public boolean isPresentationEnabled() {      return true;    }    public int travelTime() {      return _people_travelTime_xjal();    }    public int nPoorest() {      return _people_nPoorest_xjal();    }    public int nPoor() {      return _people_nPoor_xjal();    }    public int nMiddle() {      return _people_nMiddle_xjal();    }    public int nRich() {      return _people_nRich_xjal();    }    public int nRichest() {      return _people_nRichest_xjal();    }    public int hospitalAssigned() {      return _people_hospitalAssigned_xjal();    }    public int noCare() {      return _people_noCare_xjal();    }  }    @AnyLogicCustomProposalType(value = AnyLogicCustomProposalType.Label.POPULATION, customText = "Person")  public _people_Population people = new _people_Population( this );  public String getNameOf( AgentList<?> aolist ) {    if( aolist == hospitals ) return "hospitals";    if( aolist == people ) return "people";    return super.getNameOf( aolist );  }    public AgentAnimationSettings getAnimationSettingsOf( AgentList<?> aolist ) {    if( aolist == hospitals ) return _hospitals_animationSettings_xjal;    if( aolist == people ) return _people_animationSettings_xjal;    return super.getAnimationSettingsOf( aolist );  }  /**   * Returns the agent element at the specified position in hospitals   * population.   * @see com.anylogic.engine.AgentList#get(int)   * @since 7.3.7   */  public Hospital hospitals(int index) {    return hospitals.get( index );  }  /**   * This method creates and adds new embedded object in the replicated embedded object collection hospitals<br>   * @return newly created embedded object   */  public Hospital add_hospitals() {    int index = hospitals.size();    Hospital _result_xjal = instantiate_hospitals_xjal( index );    hospitals.callSetupParameters( _result_xjal, index );    hospitals.callCreate( _result_xjal, index );    _result_xjal.start();    return _result_xjal;  }  /**   * This method creates and adds new embedded object in the replicated embedded object collection hospitals<br>   * This method uses given parameter values to setup created embedded object<br>   * Index of this new embedded object instance can be obtained through calling <code>hospitals.size()</code> method <strong>before</strong> this method is called   * @param id   * @param latitude   * @param longitude   * @param q   * @param cost   * @return newly created embedded object   */  public Hospital add_hospitals( String id, double latitude, double longitude, double q, double cost ) {    int index = hospitals.size();    Hospital _result_xjal = instantiate_hospitals_xjal( index );    // Setup parameters    _result_xjal.markParametersAreSet();    _result_xjal.id = id;    _result_xjal.latitude = latitude;    _result_xjal.longitude = longitude;    _result_xjal.q = q;    _result_xjal.cost = cost;    // Finish embedded object creation    hospitals.callCreate( _result_xjal, index );    _result_xjal.start();    return _result_xjal;  }  /**   * This method removes the given embedded object from the replicated embedded object collection hospitals<br>   * The given object is destroyed, but not immediately in common case.   * @param object the active object - element of replicated embedded object hospitals - which should be removed   * @return <code>true</code> if object was removed successfully, <code>false</code> if it doesn't belong to hospitals   */  public boolean remove_hospitals( Hospital object ) {    if( ! hospitals._remove( object ) ) {      return false;    }    object.removeFromFlowchart();    object.setDestroyed();    return true;  }  /**   * Returns the agent element at the specified position in people   * population.   * @see com.anylogic.engine.AgentList#get(int)   * @since 7.3.7   */  public Person people(int index) {    return people.get( index );  }  /**   * This method creates and adds new embedded object in the replicated embedded object collection people<br>   * @return newly created embedded object   */  public Person add_people() {    int index = people.size();    Person _result_xjal = instantiate_people_xjal( index );    people.callSetupParameters( _result_xjal, index );    people.callCreate( _result_xjal, index );    _result_xjal.start();    return _result_xjal;  }  /**   * This method creates and adds new embedded object in the replicated embedded object collection people<br>   * This method uses given parameter values to setup created embedded object<br>   * Index of this new embedded object instance can be obtained through calling <code>people.size()</code> method <strong>before</strong> this method is called   * @param longitude   * @param latitude   * @param wealth   * @param educ   * @param age   * @param male   * @param income   * @return newly created embedded object   */  public Person add_people( double longitude, double latitude, int wealth, String educ, int age, int male, double income ) {    int index = people.size();    Person _result_xjal = instantiate_people_xjal( index );    // Setup parameters    _result_xjal.markParametersAreSet();    _result_xjal.longitude = longitude;    _result_xjal.latitude = latitude;    _result_xjal.wealth = wealth;    _result_xjal.educ = educ;    _result_xjal.age = age;    _result_xjal.male = male;    _result_xjal.income = income;    // Finish embedded object creation    people.callCreate( _result_xjal, index );    _result_xjal.start();    return _result_xjal;  }  /**   * This method removes the given embedded object from the replicated embedded object collection people<br>   * The given object is destroyed, but not immediately in common case.   * @param object the active object - element of replicated embedded object people - which should be removed   * @return <code>true</code> if object was removed successfully, <code>false</code> if it doesn't belong to people   */  public boolean remove_people( Person object ) {    if( ! people._remove( object ) ) {      return false;    }    object.removeFromFlowchart();    object.setDestroyed();    return true;  }  /**   * Creates an embedded object instance and adds it to the end of replicated embedded object list<br>   * <i>This method should not be called by user</i>   */  protected Hospital instantiate_hospitals_xjal( final int index ) {    Hospital _result_xjal = new Hospital( getEngine(), this, hospitals );        hospitals._add( _result_xjal );    return _result_xjal;  }  /**   * Setups parameters of an embedded object instance<br>   * This method should not be called by user   */  private void setupParameters_hospitals_xjal( final Hospital self, final int index ) {    setupParameters_hospitals_xjal( self, index, null );  }  /**   * Setups an embedded object instance<br>   * This method should not be called by user   */  @AnyLogicInternalCodegenAPI  private void create_hospitals_xjal( Hospital self, final int index ) {    create_hospitals_xjal(self, index, null );  }  /**   * Setups parameters of an embedded object instance<br>   * This method should not be called by user   */  private void setupParameters_hospitals_xjal( final Hospital self, final int index, TableInput _t ) {    if (_t != null) {      self.id = _t.getValue( "id", String.class );    } else {    self.id = self._id_DefaultValue_xjal();    }    if (_t != null) {      self.latitude = _t.getValue( "latitude", double.class );    } else {    self.latitude = self._latitude_DefaultValue_xjal();    }    if (_t != null) {      self.longitude = _t.getValue( "longitude", double.class );    } else {    self.longitude = self._longitude_DefaultValue_xjal();    }    if (_t != null) {      self.q = _t.getValue( "q_score", double.class );    } else {    self.q = self._q_DefaultValue_xjal();    }    if (_t != null) {      self.cost = _t.getValue( "cost_laparotomy", double.class );    } else {    self.cost = self._cost_DefaultValue_xjal();    }  }  /**   * Setups an embedded object instance<br>   * This method should not be called by user   */  @AnyLogicInternalCodegenAPI  private void create_hospitals_xjal( Hospital self, final int index, TableInput _t ) {	double _initial_speed = 0 ;	self.setSpeed(_initial_speed, MPS);    self.setEnvironment( this );    double _x_xjal = self.latitude ;    double _y_xjal = self.longitude ;    self.setXY( _x_xjal, _y_xjal );    self.create();    // Port connections  }  /**   * Creates an embedded object instance and adds it to the end of replicated embedded object list<br>   * <i>This method should not be called by user</i>   */  protected Person instantiate_people_xjal( final int index ) {    Person _result_xjal = new Person( getEngine(), this, people );        people._add( _result_xjal );    return _result_xjal;  }  /**   * Setups parameters of an embedded object instance<br>   * This method should not be called by user   */  private void setupParameters_people_xjal( final Person self, final int index ) {    setupParameters_people_xjal( self, index, null );  }  /**   * Setups an embedded object instance<br>   * This method should not be called by user   */  @AnyLogicInternalCodegenAPI  private void create_people_xjal( Person self, final int index ) {    create_people_xjal(self, index, null );  }  /**   * Setups parameters of an embedded object instance<br>   * This method should not be called by user   */  private void setupParameters_people_xjal( final Person self, final int index, TableInput _t ) {    if (_t != null) {      self.longitude = _t.getValue( "longitude", double.class );    } else {    self.longitude = self._longitude_DefaultValue_xjal();    }    if (_t != null) {      self.latitude = _t.getValue( "latitude", double.class );    } else {    self.latitude = self._latitude_DefaultValue_xjal();    }    if (_t != null) {      self.wealth = _t.getValue( "wealth", int.class );    } else {    self.wealth = self._wealth_DefaultValue_xjal();    }    if (_t != null) {      self.educ = _t.getValue( "educ", String.class );    } else {    self.educ = self._educ_DefaultValue_xjal();    }    if (_t != null) {      self.age = _t.getValue( "age", int.class );    } else {    self.age = self._age_DefaultValue_xjal();    }    if (_t != null) {      self.male = _t.getValue( "male", int.class );    } else {    self.male = self._male_DefaultValue_xjal();    }    if (_t != null) {      self.income = _t.getValue( "income", double.class );    } else {    self.income = self._income_DefaultValue_xjal();    }  }  /**   * Setups an embedded object instance<br>   * This method should not be called by user   */  @AnyLogicInternalCodegenAPI  private void create_people_xjal( Person self, final int index, TableInput _t ) {	double _initial_speed = 30 //(randomTrue(0.014)) ? 10.0 : 1.34 ;	self.setSpeed(_initial_speed, KPH);    self.setEnvironment( this );    double _x_xjal = self.latitude ;    double _y_xjal = self.longitude ;    self.setXY( _x_xjal, _y_xjal );    self.create();    // Port connections  }  /**   * <i>This method should not be called by user</i>   */  private int _hospitals_vTooBig_xjal() {    int _value = 0;    for ( Hospital item : hospitals ) {      boolean _t = item.vTooBig ;      if ( _t ) {        _value++;      }    }    return _value;  }  /**   * <i>This method should not be called by user</i>   */  private double _hospitals_avgCost_xjal() {    int _cnt = 0;    double _value = 0;    for ( Hospital item : hospitals ) {        _cnt++;        _value += item.cost ;    }    return _cnt > 0 ? _value / _cnt : 0;  }  /**   * <i>This method should not be called by user</i>   */  private double _hospitals_avgQ_xjal() {    int _cnt = 0;    double _value = 0;    for ( Hospital item : hospitals ) {        _cnt++;        _value += item.q ;    }    return _cnt > 0 ? _value / _cnt : 0;  }  /**   * <i>This method should not be called by user</i>   */  private double _hospitals_costMinusVoucher_xjal() {    int _cnt = 0;    double _value = 0;    for ( Hospital item : hospitals ) {        _cnt++;        _value += item.cost - voucher ;    }    return _cnt > 0 ? _value / _cnt : 0;  }  /**   * <i>This method should not be called by user</i>   */  private int _people_travelTime_xjal() {    int _value = 0;    for ( Person item : people ) {      boolean _t = item.travelTime > 2.0 ;      if ( _t ) {        _value++;      }    }    return _value;  }  /**   * <i>This method should not be called by user</i>   */  private int _people_nPoorest_xjal() {    int _value = 0;    for ( Person item : people ) {      boolean _t = item.wealth == 1 ;      if ( _t ) {        _value++;      }    }    return _value;  }  /**   * <i>This method should not be called by user</i>   */  private int _people_nPoor_xjal() {    int _value = 0;    for ( Person item : people ) {      boolean _t = item.wealth == 2 ;      if ( _t ) {        _value++;      }    }    return _value;  }  /**   * <i>This method should not be called by user</i>   */  private int _people_nMiddle_xjal() {    int _value = 0;    for ( Person item : people ) {      boolean _t = item.wealth == 3 ;      if ( _t ) {        _value++;      }    }    return _value;  }  /**   * <i>This method should not be called by user</i>   */  private int _people_nRich_xjal() {    int _value = 0;    for ( Person item : people ) {      boolean _t = item.wealth == 4 ;      if ( _t ) {        _value++;      }    }    return _value;  }  /**   * <i>This method should not be called by user</i>   */  private int _people_nRichest_xjal() {    int _value = 0;    for ( Person item : people ) {      boolean _t = item.wealth == 5 ;      if ( _t ) {        _value++;      }    }    return _value;  }  /**   * <i>This method should not be called by user</i>   */  private int _people_hospitalAssigned_xjal() {    int _value = 0;    for ( Person item : people ) {      boolean _t = item.destHospital != null ;      if ( _t ) {        _value++;      }    }    return _value;  }  /**   * <i>This method should not be called by user</i>   */  private int _people_noCare_xjal() {    int _value = 0;    for ( Person item : people ) {      boolean _t = item.noCare == 1 ;      if ( _t ) {        _value++;      }    }    return _value;  }  public int popSize;  public void update_popSize() {    popSize = people.size() ;  }  public void update_popSize(int _value) {    popSize = _value;  }  public int hospitalAssigned;  public void update_hospitalAssigned() {    hospitalAssigned = people.hospitalAssigned() ;  }  public void update_hospitalAssigned(int _value) {    hospitalAssigned = _value;  }  public double nNoCare;  public void update_nNoCare() {    nNoCare = (double) people.noCare() / (double) this.popSize ;  }  public void update_nNoCare(double _value) {    nNoCare = _value;  }  public double vTooBig;  public void update_vTooBig() {    vTooBig = (double) hospitals.vTooBig()  ;  }  public void update_vTooBig(double _value) {    vTooBig = _value;  }  public double costMinusVoucher;  public void update_costMinusVoucher() {    costMinusVoucher = (double) hospitals.costMinusVoucher() ;  }  public void update_costMinusVoucher(double _value) {    costMinusVoucher = _value;  }  // View areas  public ViewArea _origin_VA = new ViewArea( this, "[Origin]", 0, 0, 1550.0, 890.0 );  @Override  @AnyLogicInternalCodegenAPI  public int getViewAreas(Map<String, ViewArea> _output) {    if ( _output != null ) {      _output.put( "_origin_VA", this._origin_VA );    }    return 1 + super.getViewAreas( _output );  }  @AnyLogicInternalCodegenAPI  protected static final Font _text2_Font = new Font("Serif", 0, 18 );  @AnyLogicInternalCodegenAPI  protected static final Font _text3_Font = _text2_Font;  @AnyLogicInternalCodegenAPI  protected static final Font _MeanText1_Font = new Font("Serif", 0, 30 );  @AnyLogicInternalCodegenAPI  protected static final Font _MeanText2_Font = _MeanText1_Font;  @AnyLogicInternalCodegenAPI  protected static final int _rectangle2 = 1;  @AnyLogicInternalCodegenAPI  protected static final int _rectangle1 = 2;  @AnyLogicInternalCodegenAPI  protected static final int _map = 3;  @AnyLogicInternalCodegenAPI  protected static final int _people_presentation = 4;  @AnyLogicInternalCodegenAPI  protected static final int _hospitals_presentation = 5;  @AnyLogicInternalCodegenAPI  protected static final int _text2 = 6;  @AnyLogicInternalCodegenAPI  protected static final int _text3 = 7;  @AnyLogicInternalCodegenAPI  protected static final int _MeanText1 = 8;  @AnyLogicInternalCodegenAPI  protected static final int _MeanText2 = 9;  @AnyLogicInternalCodegenAPI  protected static final int _Blantyre = 10;  @AnyLogicInternalCodegenAPI  protected static final int _Lilongwe = 11;  /** Internal constant, shouldn't be accessed by user */  @AnyLogicInternalCodegenAPI  protected static final int _SHAPE_NEXT_ID_xjal = 12;   /**   * Top-level presentation group id   */	   @AnyLogicInternalCodegenAPI  protected static final int _presentation = 0;  @AnyLogicInternalCodegenAPI  public boolean isPublicPresentationDefined() {    return true;  }  @AnyLogicInternalCodegenAPI  public boolean isEmbeddedAgentPresentationVisible( Agent _a ) {    return super.isEmbeddedAgentPresentationVisible( _a );  }  /**   * Top-level icon group id   */	   @AnyLogicInternalCodegenAPI  protected static final int _icon = -1;      protected ShapeRectangle rectangle2;  protected ShapeRectangle rectangle1;  protected ShapeGISMap map;    /**   * <i>This method should not be called by user</i>   */  @AnyLogicInternalCodegenAPI  protected ShapeEmbeddedObjectPresentation _people_presentation_createShapeWithStaticProperties_xjal( final Agent _a, final int _index ) {    ShapeEmbeddedObjectPresentation shape = new ShapeEmbeddedObjectPresentation( Main.this, SHAPE_DRAW_2D3D, true, 9.5, -13.7, 0.0, 0.0,		false, true, _a );    return shape;  }  protected ShapeAgentPopulationGroup people_presentation;    /**   * <i>This method should not be called by user</i>   */  @AnyLogicInternalCodegenAPI  protected ShapeEmbeddedObjectPresentation _hospitals_presentation_createShapeWithStaticProperties_xjal( final Agent _a, final int _index ) {    ShapeEmbeddedObjectPresentation shape = new ShapeEmbeddedObjectPresentation( Main.this, SHAPE_DRAW_2D3D, true, 9.5, -13.7, 0.0, 0.0,		false, true, _a );    return shape;  }  protected ShapeAgentPopulationGroup hospitals_presentation;  protected ShapeText text2;  protected ShapeText text3;  protected ShapeText MeanText1;  protected ShapeText MeanText2;  protected GISRegion Blantyre;  protected GISRegion Lilongwe;  @AnyLogicInternalCodegenAPI  private void _createPersistentElementsBP0_xjal() {    rectangle2 = new ShapeRectangle(       SHAPE_DRAW_2D3D, true,-690.0, 420.0, 0.0, 0.0,             black, white,			450.0, 110.0, 10.0, 1.0, LINE_STYLE_SOLID );    rectangle1 = new ShapeRectangle(       SHAPE_DRAW_2D3D, true,-690.0, 10.0, 0.0, 0.0,             black, white,			450.0, 390.0, 10.0, 1.0, LINE_STYLE_SOLID );    text2 = new ShapeText(        SHAPE_DRAW_2D, true,-660.0, 70.0, 0.0, 0.0,         black,"Means",        _text2_Font, ALIGNMENT_LEFT );    text3 = new ShapeText(        SHAPE_DRAW_2D, true,-490.0, 70.0, 0.0, 0.0,         black,"SDs",        _text3_Font, ALIGNMENT_LEFT );    MeanText1 = new ShapeText(        SHAPE_DRAW_2D, true,-670.0, 30.0, 0.0, 0.0,         black,"Taste Parameters",        _MeanText1_Font, ALIGNMENT_LEFT );    MeanText2 = new ShapeText(        SHAPE_DRAW_2D, true,-680.0, 430.0, 0.0, 0.0,         black,"Other parameters",        _MeanText2_Font, ALIGNMENT_LEFT );    Blantyre = new GISRegion( map, true, this.<double[]>getElementProperty("Blantyre", IElementDescriptor.LAT_LON_PAIRS), defaultGisFillColor, brown, 1.0, LINE_STYLE_DASHED, "Blantyre, Southern Region, Malawi", 2.3209617548125E9 );    Lilongwe = new GISRegion( map, true, this.<double[]>getElementProperty("Lilongwe", IElementDescriptor.LAT_LON_PAIRS), defaultGisFillColor, brown, 1.0, LINE_STYLE_DASHED, "Lilongwe, Central Region, Malawi", 6.205527466875E9 );  }  @AnyLogicInternalCodegenAPI  private void _createPersistentElementsAP0_xjal() {    people_presentation = new ShapeAgentPopulationGroup(this, SHAPE_DRAW_2D3D, true, people) {          @Override      public ShapeEmbeddedObjectPresentation createShapeWithStaticProperties_xjal( final Agent _a, int index ) {        ShapeEmbeddedObjectPresentation _e = _people_presentation_createShapeWithStaticProperties_xjal( _a, index );        return _e;      }    };    hospitals_presentation = new ShapeAgentPopulationGroup(this, SHAPE_DRAW_2D3D, true, hospitals) {          @Override      public ShapeEmbeddedObjectPresentation createShapeWithStaticProperties_xjal( final Agent _a, int index ) {        ShapeEmbeddedObjectPresentation _e = _hospitals_presentation_createShapeWithStaticProperties_xjal( _a, index );        return _e;      }    };  }  // Static initialization of persistent elements  {    map = new ShapeGISMap(		Main.this, SHAPE_DRAW_2D3D, true, 0.0, 0.0,			530.0, 480.0, "/malawi_recentralization_model_2017_06_04/", new ShapeGISMap.Layer[] { }, 9.805066814588644, -13.332815730562562, 1.0E-6, silver, 		white, true, 		TileURLProviderType.HUMANITARIAN, 		ShapeGISMap.STRAIGHT, 		1, false );    _createPersistentElementsBP0_xjal();  }  protected ShapeTopLevelPresentationGroup presentation;  protected ShapeModelElementsGroup icon;   @Override  @AnyLogicInternalCodegenAPI  public ShapeTopLevelPresentationGroup getPresentationShape() {    return presentation;  }  @Override  @AnyLogicInternalCodegenAPI  public ShapeModelElementsGroup getModelElementsShape() {    return icon;  }  @Override  @AnyLogicInternalCodegenAPI  public Object getPersistentShape( int _shape ) {    switch (_shape) {      case _presentation: return presentation;      case _icon: return icon;      case _rectangle2: return rectangle2;      case _rectangle1: return rectangle1;      case _map: return map;      case _people_presentation: return people_presentation;      case _hospitals_presentation: return hospitals_presentation;      case _text2: return text2;      case _text3: return text3;      case _MeanText1: return MeanText1;      case _MeanText2: return MeanText2;      case _Blantyre: return Blantyre;      case _Lilongwe: return Lilongwe;      default: return super.getPersistentShape( _shape );     }  }  @Override  @AnyLogicInternalCodegenAPI  public String getNameOfShape_xjal( Object _shape ) {    try {      if ( _shape == null ) return null;      String _name_xjal;      _name_xjal = checkNameOfShape_xjal( _shape, presentation, "presentation" ); if (_name_xjal != null) return _name_xjal;      _name_xjal = checkNameOfShape_xjal( _shape, icon, "icon" ); if (_name_xjal != null) return _name_xjal;      _name_xjal = checkNameOfShape_xjal( _shape, rectangle2, "rectangle2" ); if (_name_xjal != null) return _name_xjal;      _name_xjal = checkNameOfShape_xjal( _shape, rectangle1, "rectangle1" ); if (_name_xjal != null) return _name_xjal;      _name_xjal = checkNameOfShape_xjal( _shape, map, "map" ); if (_name_xjal != null) return _name_xjal;      _name_xjal = checkNameOfShape_xjal( _shape, people_presentation, "people_presentation" ); if (_name_xjal != null) return _name_xjal;      _name_xjal = checkNameOfShape_xjal( _shape, hospitals_presentation, "hospitals_presentation" ); if (_name_xjal != null) return _name_xjal;      _name_xjal = checkNameOfShape_xjal( _shape, text2, "text2" ); if (_name_xjal != null) return _name_xjal;      _name_xjal = checkNameOfShape_xjal( _shape, text3, "text3" ); if (_name_xjal != null) return _name_xjal;      _name_xjal = checkNameOfShape_xjal( _shape, MeanText1, "MeanText1" ); if (_name_xjal != null) return _name_xjal;      _name_xjal = checkNameOfShape_xjal( _shape, MeanText2, "MeanText2" ); if (_name_xjal != null) return _name_xjal;      _name_xjal = checkNameOfShape_xjal( _shape, Blantyre, "Blantyre" ); if (_name_xjal != null) return _name_xjal;      _name_xjal = checkNameOfShape_xjal( _shape, Lilongwe, "Lilongwe" ); if (_name_xjal != null) return _name_xjal;    } catch (Exception e) {      return null;    }    return super.getNameOfShape_xjal( _shape );  }  @AnyLogicInternalCodegenAPI  private void drawModelElements_Parameters_xjal(Panel _panel, Graphics2D _g, boolean _publicOnly, boolean _isSuperClass ) {    if (!_publicOnly) {      drawParameter( _panel, _g, -650, 120, 10, 0, "male", male, 0 );    }    if (!_publicOnly) {      drawParameter( _panel, _g, -650, 300, 10, 0, "distance", distance, 0 );    }    if (!_publicOnly) {      drawParameter( _panel, _g, -650, 240, 10, 0, "educPrimaryComplete", educPrimaryComplete, 0 );    }    if (!_publicOnly) {      drawParameter( _panel, _g, -650, 220, 10, 0, "educPrimary", educPrimary, 0 );    }    if (!_publicOnly) {      drawParameter( _panel, _g, -650, 200, 10, 0, "age50", age50, 0 );    }    if (!_publicOnly) {      drawParameter( _panel, _g, -650, 180, 10, 0, "age2249", age2249, 0 );    }    if (!_publicOnly) {      drawParameter( _panel, _g, -650, 160, 10, 0, "age1521", age1521, 0 );    }    if (!_publicOnly) {      drawParameter( _panel, _g, -650, 140, 10, 0, "age514", age514, 0 );    }    if (!_publicOnly) {      drawParameter( _panel, _g, -650, 320, 10, 0, "q", q, 0 );    }    if (!_publicOnly) {      drawParameter( _panel, _g, -650, 100, 10, 0, "intercept", intercept, 0 );    }    if (!_publicOnly) {      drawParameter( _panel, _g, -650, 280, 10, 0, "educSecondaryComplete", educSecondaryComplete, 0 );    }    if (!_publicOnly) {      drawParameter( _panel, _g, -650, 260, 10, 0, "educSecondary", educSecondary, 0 );    }    if (!_publicOnly) {      drawParameter( _panel, _g, -650, 360, 10, 0, "logIncomeSq", logIncomeSq, 0 );    }    if (!_publicOnly) {      drawParameter( _panel, _g, -650, 340, 10, 0, "logIncome", logIncome, 0 );    }    if (!_publicOnly) {      drawParameter( _panel, _g, -480, 120, 10, 0, "maleSE", maleSE, 0 );    }    if (!_publicOnly) {      drawParameter( _panel, _g, -480, 300, 10, 0, "distanceSE", distanceSE, 0 );    }    if (!_publicOnly) {      drawParameter( _panel, _g, -480, 240, 10, 0, "educPrimaryCompleteSE", educPrimaryCompleteSE, 0 );    }    if (!_publicOnly) {      drawParameter( _panel, _g, -480, 220, 10, 0, "educPrimarySE", educPrimarySE, 0 );    }    if (!_publicOnly) {      drawParameter( _panel, _g, -480, 200, 10, 0, "age50SE", age50SE, 0 );    }    if (!_publicOnly) {      drawParameter( _panel, _g, -480, 180, 10, 0, "age2249SE", age2249SE, 0 );    }    if (!_publicOnly) {      drawParameter( _panel, _g, -480, 160, 10, 0, "age1521SE", age1521SE, 0 );    }    if (!_publicOnly) {      drawParameter( _panel, _g, -480, 140, 10, 0, "age514SE", age514SE, 0 );    }    if (!_publicOnly) {      drawParameter( _panel, _g, -480, 320, 10, 0, "qSE", qSE, 0 );    }    if (!_publicOnly) {      drawParameter( _panel, _g, -480, 100, 10, 0, "interceptSE", interceptSE, 0 );    }    if (!_publicOnly) {      drawParameter( _panel, _g, -480, 280, 10, 0, "educSecondaryCompleteSE", educSecondaryCompleteSE, 0 );    }    if (!_publicOnly) {      drawParameter( _panel, _g, -480, 260, 10, 0, "educSecondarySE", educSecondarySE, 0 );    }    if (!_publicOnly) {      drawParameter( _panel, _g, -480, 360, 10, 0, "logIncomeSqSE", logIncomeSqSE, 0 );    }    if (!_publicOnly) {      drawParameter( _panel, _g, -480, 340, 10, 0, "logIncomeSE", logIncomeSE, 0 );    }    if (!_publicOnly) {      drawParameter( _panel, _g, -650, 380, 10, 0, "iv", iv, 0 );    }    if (!_publicOnly) {      drawParameter( _panel, _g, -480, 380, 10, 0, "ivSE", ivSE, 0 );    }    if (!_publicOnly) {      drawParameter( _panel, _g, -660, 470, 10, 0, "phi", phi, 0 );    }    if (!_publicOnly) {      drawParameter( _panel, _g, 750, 150, 10, 0, "voucher", voucher, 0 );    }  }  @AnyLogicInternalCodegenAPI  private void drawModelElements_PlainVariables_xjal(Panel _panel, Graphics2D _g, boolean _publicOnly, boolean _isSuperClass ) {    if (!_publicOnly) {      drawPlainVariable( _panel, _g, -160, 100, 10, 0, "vAgentID", vAgentID, false );    }  }  @AnyLogicInternalCodegenAPI  private void drawModelElements_CollectionVariables_xjal(Panel _panel, Graphics2D _g, boolean _publicOnly, boolean _isSuperClass ) {    if (!_publicOnly) {      drawCollection( _panel, _g, -160, 260, 10, 0, "hospitalList", hospitalList );    }  }  @AnyLogicInternalCodegenAPI  private void drawModelElements_Outputs_xjal(Panel _panel, Graphics2D _g, boolean _publicOnly, boolean _isSuperClass ) {    if (!_publicOnly) {      drawOutput( _panel, _g, 750, 30, 15, 0, "popSize", popSize );    }    if (!_publicOnly) {      drawOutput( _panel, _g, 850, 30, 15, 0, "hospitalAssigned", hospitalAssigned );    }    if (!_publicOnly) {      drawOutput( _panel, _g, 1000, 30, 15, 0, "nNoCare", nNoCare );    }    if (!_publicOnly) {      drawOutput( _panel, _g, 750, 70, 15, 0, "vTooBig", vTooBig );    }    if (!_publicOnly) {      drawOutput( _panel, _g, 850, 70, 15, 0, "costMinusVoucher", costMinusVoucher );    }  }  @AnyLogicInternalCodegenAPI  private void drawModelElements_EmbeddeObjects_xjal(Panel _panel, Graphics2D _g, boolean _publicOnly, boolean _isSuperClass ) {    // Embedded object "hospitals"    if (!_publicOnly) {      drawEmbeddedObjectModelDefault( _panel, _g, -160 , 40 , 10, 0, "hospitals", this.hospitals );    }    // Embedded object "people"    if (!_publicOnly) {      drawEmbeddedObjectModelDefault( _panel, _g, -160 , 70 , 10, 0, "people", this.people );    }  }  @AnyLogicInternalCodegenAPI  private void drawModelElements_AgentLinks_xjal(Panel _panel, Graphics2D _g, boolean _publicOnly, boolean _isSuperClass ) {    if (_publicOnly) { return; }      drawLinkToAgent( _panel, _g, 50, -50, 15, 0, "connections", true, connections );  }  @Override  @AnyLogicInternalCodegenAPI  public void drawModelElements( Panel _panel, Graphics2D _g, boolean _publicOnly, boolean _isSuperClass ) {  	super.drawModelElements( _panel, _g, _publicOnly, true );    drawModelElements_Parameters_xjal( _panel, _g, _publicOnly, _isSuperClass );    drawModelElements_PlainVariables_xjal( _panel, _g, _publicOnly, _isSuperClass );    drawModelElements_CollectionVariables_xjal( _panel, _g, _publicOnly, _isSuperClass );    drawModelElements_Outputs_xjal( _panel, _g, _publicOnly, _isSuperClass );    drawModelElements_EmbeddeObjects_xjal( _panel, _g, _publicOnly, _isSuperClass );    drawModelElements_AgentLinks_xjal( _panel, _g, _publicOnly, _isSuperClass );  }  @AnyLogicInternalCodegenAPI  private boolean onClickModelAt_EmbeddedObjects_xjal( Panel _panel, double _x, double _y, int _clickCount, boolean _publicOnly, boolean _isSuperClass ) {    if ( !hospitals.isEmpty() && modelElementContains(_x, _y, -160, 40) ) {      if ( _clickCount == 2 ) {        _panel.browseAgent_xjal( -160, 40, this, "hospitals" );      } else {        _panel.addInspect( -160, 40, this, "hospitals" );      }      return true;    }    if ( !people.isEmpty() && modelElementContains(_x, _y, -160, 70) ) {      if ( _clickCount == 2 ) {        _panel.browseAgent_xjal( -160, 70, this, "people" );      } else {        _panel.addInspect( -160, 70, this, "people" );      }      return true;    }    return false;  }  @AnyLogicInternalCodegenAPI  private boolean onClickModelAt_AgentLinks_xjal( Panel _panel, double _x, double _y, int _clickCount, boolean _publicOnly, boolean _isSuperClass ) {    if ( modelElementContains(_x, _y, 50, -50) ) {        _panel.addInspect_xjal( 50, -50, this, "connections", Panel.INSPECT_CONNECTIONS_xjal );      return true;    }    return false;  }  @AnyLogicInternalCodegenAPI  private boolean onClickModelAt_Parameters_xjal( Panel _panel, double _x, double _y, int _clickCount, boolean _publicOnly, boolean _isSuperClass ) {    if( !_publicOnly && modelElementContains(_x, _y, -650, 120) ) {      _panel.addInspect( -650, 120, this, "male" );       return true;    }    if( !_publicOnly && modelElementContains(_x, _y, -650, 300) ) {      _panel.addInspect( -650, 300, this, "distance" );       return true;    }    if( !_publicOnly && modelElementContains(_x, _y, -650, 240) ) {      _panel.addInspect( -650, 240, this, "educPrimaryComplete" );       return true;    }    if( !_publicOnly && modelElementContains(_x, _y, -650, 220) ) {      _panel.addInspect( -650, 220, this, "educPrimary" );       return true;    }    if( !_publicOnly && modelElementContains(_x, _y, -650, 200) ) {      _panel.addInspect( -650, 200, this, "age50" );       return true;    }    if( !_publicOnly && modelElementContains(_x, _y, -650, 180) ) {      _panel.addInspect( -650, 180, this, "age2249" );       return true;    }    if( !_publicOnly && modelElementContains(_x, _y, -650, 160) ) {      _panel.addInspect( -650, 160, this, "age1521" );       return true;    }    if( !_publicOnly && modelElementContains(_x, _y, -650, 140) ) {      _panel.addInspect( -650, 140, this, "age514" );       return true;    }    if( !_publicOnly && modelElementContains(_x, _y, -650, 320) ) {      _panel.addInspect( -650, 320, this, "q" );       return true;    }    if( !_publicOnly && modelElementContains(_x, _y, -650, 100) ) {      _panel.addInspect( -650, 100, this, "intercept" );       return true;    }    if( !_publicOnly && modelElementContains(_x, _y, -650, 280) ) {      _panel.addInspect( -650, 280, this, "educSecondaryComplete" );       return true;    }    if( !_publicOnly && modelElementContains(_x, _y, -650, 260) ) {      _panel.addInspect( -650, 260, this, "educSecondary" );       return true;    }    if( !_publicOnly && modelElementContains(_x, _y, -650, 360) ) {      _panel.addInspect( -650, 360, this, "logIncomeSq" );       return true;    }    if( !_publicOnly && modelElementContains(_x, _y, -650, 340) ) {      _panel.addInspect( -650, 340, this, "logIncome" );       return true;    }    if( !_publicOnly && modelElementContains(_x, _y, -480, 120) ) {      _panel.addInspect( -480, 120, this, "maleSE" );       return true;    }    if( !_publicOnly && modelElementContains(_x, _y, -480, 300) ) {      _panel.addInspect( -480, 300, this, "distanceSE" );       return true;    }    if( !_publicOnly && modelElementContains(_x, _y, -480, 240) ) {      _panel.addInspect( -480, 240, this, "educPrimaryCompleteSE" );       return true;    }    if( !_publicOnly && modelElementContains(_x, _y, -480, 220) ) {      _panel.addInspect( -480, 220, this, "educPrimarySE" );       return true;    }    if( !_publicOnly && modelElementContains(_x, _y, -480, 200) ) {      _panel.addInspect( -480, 200, this, "age50SE" );       return true;    }    if( !_publicOnly && modelElementContains(_x, _y, -480, 180) ) {      _panel.addInspect( -480, 180, this, "age2249SE" );       return true;    }    if( !_publicOnly && modelElementContains(_x, _y, -480, 160) ) {      _panel.addInspect( -480, 160, this, "age1521SE" );       return true;    }    if( !_publicOnly && modelElementContains(_x, _y, -480, 140) ) {      _panel.addInspect( -480, 140, this, "age514SE" );       return true;    }    if( !_publicOnly && modelElementContains(_x, _y, -480, 320) ) {      _panel.addInspect( -480, 320, this, "qSE" );       return true;    }    if( !_publicOnly && modelElementContains(_x, _y, -480, 100) ) {      _panel.addInspect( -480, 100, this, "interceptSE" );       return true;    }    if( !_publicOnly && modelElementContains(_x, _y, -480, 280) ) {      _panel.addInspect( -480, 280, this, "educSecondaryCompleteSE" );       return true;    }    if( !_publicOnly && modelElementContains(_x, _y, -480, 260) ) {      _panel.addInspect( -480, 260, this, "educSecondarySE" );       return true;    }    if( !_publicOnly && modelElementContains(_x, _y, -480, 360) ) {      _panel.addInspect( -480, 360, this, "logIncomeSqSE" );       return true;    }    if( !_publicOnly && modelElementContains(_x, _y, -480, 340) ) {      _panel.addInspect( -480, 340, this, "logIncomeSE" );       return true;    }    if( !_publicOnly && modelElementContains(_x, _y, -650, 380) ) {      _panel.addInspect( -650, 380, this, "iv" );       return true;    }    if( !_publicOnly && modelElementContains(_x, _y, -480, 380) ) {      _panel.addInspect( -480, 380, this, "ivSE" );       return true;    }    if( !_publicOnly && modelElementContains(_x, _y, -660, 470) ) {      _panel.addInspect( -660, 470, this, "phi" );       return true;    }    if( !_publicOnly && modelElementContains(_x, _y, 750, 150) ) {      _panel.addInspect( 750, 150, this, "voucher" );       return true;    }    return false;  }  @AnyLogicInternalCodegenAPI  private boolean onClickModelAt_PlainVariables_xjal( Panel _panel, double _x, double _y, int _clickCount, boolean _publicOnly, boolean _isSuperClass ) {    if( !_publicOnly && modelElementContains(_x, _y, -160, 100) ) {      _panel.addInspect( -160, 100, this, "vAgentID" );       return true;    }    return false;  }  @AnyLogicInternalCodegenAPI  private boolean onClickModelAt_CollectionVariables_xjal( Panel _panel, double _x, double _y, int _clickCount, boolean _publicOnly, boolean _isSuperClass ) {    if( !_publicOnly && modelElementContains(_x, _y, -160, 260) ) {      _panel.addInspect( -160, 260, this, "hospitalList" );       return true;    }    return false;  }  @AnyLogicInternalCodegenAPI  private boolean onClickModelAt_Outputs_xjal( Panel _panel, double _x, double _y, int _clickCount, boolean _publicOnly, boolean _isSuperClass ) {    if( !_publicOnly && modelElementContains(_x, _y, 750, 30) ) {      _panel.addInspect( 750, 30, this, "popSize" );       return true;    }    if( !_publicOnly && modelElementContains(_x, _y, 850, 30) ) {      _panel.addInspect( 850, 30, this, "hospitalAssigned" );       return true;    }    if( !_publicOnly && modelElementContains(_x, _y, 1000, 30) ) {      _panel.addInspect( 1000, 30, this, "nNoCare" );       return true;    }    if( !_publicOnly && modelElementContains(_x, _y, 750, 70) ) {      _panel.addInspect( 750, 70, this, "vTooBig" );       return true;    }    if( !_publicOnly && modelElementContains(_x, _y, 850, 70) ) {      _panel.addInspect( 850, 70, this, "costMinusVoucher" );       return true;    }    return false;  }  @Override  @AnyLogicInternalCodegenAPI  public boolean onClickModelAt( Panel _panel, double _x, double _y, int _clickCount, boolean _publicOnly, boolean _isSuperClass ) {    if ( onClickModelAt_EmbeddedObjects_xjal( _panel, _x, _y, _clickCount, _publicOnly, _isSuperClass ) ) { return true; }    if ( onClickModelAt_AgentLinks_xjal( _panel, _x, _y, _clickCount, _publicOnly, _isSuperClass ) ) { return true; }    if ( onClickModelAt_Parameters_xjal( _panel, _x, _y, _clickCount, _publicOnly, _isSuperClass ) ) { return true; }    if ( onClickModelAt_PlainVariables_xjal( _panel, _x, _y, _clickCount, _publicOnly, _isSuperClass ) ) { return true; }    if ( onClickModelAt_CollectionVariables_xjal( _panel, _x, _y, _clickCount, _publicOnly, _isSuperClass ) ) { return true; }    if ( onClickModelAt_Outputs_xjal( _panel, _x, _y, _clickCount, _publicOnly, _isSuperClass ) ) { return true; }    return super.onClickModelAt( _panel, _x, _y, _clickCount, _publicOnly, true );  }  /**   * Constructor   */  public Main( Engine engine, Agent owner, AgentList<? extends Main> ownerPopulation ) {    super( engine, owner, ownerPopulation );    instantiateBaseStructureThis_xjal();  }  @AnyLogicInternalCodegenAPI  public void onOwnerChanged_xjal() {    super.onOwnerChanged_xjal();    setupReferences_xjal();  }  @AnyLogicInternalCodegenAPI  public void instantiateBaseStructure_xjal() {    super.instantiateBaseStructure_xjal();    instantiateBaseStructureThis_xjal();  }    @AnyLogicInternalCodegenAPI  private void instantiateBaseStructureThis_xjal() {    setupReferences_xjal();  }    @AnyLogicInternalCodegenAPI  private void setupReferences_xjal() {  }    /**   * Simple constructor. Please add created agent to some population by calling goToPopulation() function   */  public Main() {  }    /**   * Simple constructor. Please add created agent to some population by calling goToPopulation() function   */  public Main( double male, double distance, double educPrimaryComplete, double educPrimary, double age50, double age2249, double age1521, double age514, double q, double intercept, double educSecondaryComplete, double educSecondary, double logIncomeSq, double logIncome, double maleSE, double distanceSE, double educPrimaryCompleteSE, double educPrimarySE, double age50SE, double age2249SE, double age1521SE, double age514SE, double qSE, double interceptSE, double educSecondaryCompleteSE, double educSecondarySE, double logIncomeSqSE, double logIncomeSE, double iv, double ivSE, double phi, double voucher ) {    markParametersAreSet();    this.male = male;    this.distance = distance;    this.educPrimaryComplete = educPrimaryComplete;    this.educPrimary = educPrimary;    this.age50 = age50;    this.age2249 = age2249;    this.age1521 = age1521;    this.age514 = age514;    this.q = q;    this.intercept = intercept;    this.educSecondaryComplete = educSecondaryComplete;    this.educSecondary = educSecondary;    this.logIncomeSq = logIncomeSq;    this.logIncome = logIncome;    this.maleSE = maleSE;    this.distanceSE = distanceSE;    this.educPrimaryCompleteSE = educPrimaryCompleteSE;    this.educPrimarySE = educPrimarySE;    this.age50SE = age50SE;    this.age2249SE = age2249SE;    this.age1521SE = age1521SE;    this.age514SE = age514SE;    this.qSE = qSE;    this.interceptSE = interceptSE;    this.educSecondaryCompleteSE = educSecondaryCompleteSE;    this.educSecondarySE = educSecondarySE;    this.logIncomeSqSE = logIncomeSqSE;    this.logIncomeSE = logIncomeSE;    this.iv = iv;    this.ivSE = ivSE;    this.phi = phi;    this.voucher = voucher;  }    /**   * Creating embedded object instances   */  @AnyLogicInternalCodegenAPI  private void instantiatePopulations_xjal() {  }  @Override  @AnyLogicInternalCodegenAPI  public void doCreate() {    super.doCreate();    // Creating embedded object instances    instantiatePopulations_xjal();    // Assigning initial values for plain variables    setupPlainVariables_Main_xjal();    // Dynamic initialization of persistent elements    _createPersistentElementsAP0_xjal();    presentation = new ShapeTopLevelPresentationGroup( Main.this, true, 0, 0, 0, 0 , rectangle2, rectangle1, map, people_presentation, hospitals_presentation, text2, text3, MeanText1, MeanText2 );    // Creating contents for replicated shapes    hospitals_presentation.createShapes();    people_presentation.createShapes();    // Creating embedded object instances    instantiatePopulations_xjal();    icon = new ShapeModelElementsGroup( Main.this, getElementProperty( "malawi_recentralization_model_2017_06_04.Main.icon", IElementDescriptor.MODEL_ELEMENT_DESCRIPTORS )  );    icon.setIconOffsets( 0.0, 0.0 );    // Environments setup    setupSpace( map );    disableSteps();    setNetworkUserDefined();	 // Port connectors with non-replicated objects    // Creating replicated embedded objects    hospitals.setEnvironment( this );    hospitals.fillFromTable( new TableInput( this, (java.util.function.Supplier<ResultSet> & Serializable) () -> selectResultSet(	"SELECT * FROM hospitals;")), null, true, false );    people.setEnvironment( this );    people.fillFromTable( new TableInput( this, (java.util.function.Supplier<ResultSet> & Serializable) () -> selectResultSet(	"SELECT * FROM pop_rand;")), null, true, false );    setupInitialConditions_xjal( Main.class );  }  @AnyLogicInternalCodegenAPI  public void setupExt_xjal(AgentExtension _ext) {    // Agent properties setup    if ( _ext instanceof ExtAgentWithSpatialMetrics && _ext instanceof ExtWithSpaceType ) {      double _value;      _value = 10 ;      ((ExtAgentWithSpatialMetrics) _ext).setSpeed( _value, MPS );    }  }  @Override  @AnyLogicInternalCodegenAPI  public void doStart() {    super.doStart();    for (Agent embeddedObject : hospitals){      embeddedObject.start();    }    for (Agent embeddedObject : people){      embeddedObject.start();    }  }   @AnyLogicInternalCodegenAPI  public void onStartup() {    super.onStartup();    update_popSize();//System.out.println("Agent\tHospital\tbIntercept\tbMale\tMale\tbAge\tbEduc\tCost\tbLogIncome\tbLogIncomeSq\tIncome\tc\tbDistance\tDistance\tbQ\tq\tbIV\tU");   }  /**   * Assigning initial values for plain variables<br>   * <em>This method isn't designed to be called by user and may be removed in future releases.</em>   */  @AnyLogicInternalCodegenAPI  public void setupPlainVariables_xjal() {    setupPlainVariables_Main_xjal();  }  /**   * Assigning initial values for plain variables<br>   * <em>This method isn't designed to be called by user and may be removed in future releases.</em>   */  @AnyLogicInternalCodegenAPI  private void setupPlainVariables_Main_xjal() {    vAgentID = 0 ;  }  // User API -----------------------------------------------------  @AnyLogicInternalCodegenAPI  static LinkToAgentAnimationSettings _connections_commonAnimationSettings_xjal = new LinkToAgentAnimationSettingsImpl( false, black, 1.0, LINE_STYLE_SOLID, ARROW_NONE, 0.0 );  public LinkToAgentCollection<Agent, Agent> connections = new LinkToAgentStandardImpl<Agent, Agent>(this, _connections_commonAnimationSettings_xjal);  @Override  public LinkToAgentCollection<? extends Agent, ? extends Agent> getLinkToAgentStandard_xjal() {    return connections;  }  @AnyLogicInternalCodegenAPI  public void drawLinksToAgents(boolean _underAgents_xjal, LinkToAgentAnimator _animator_xjal) {    super.drawLinksToAgents(_underAgents_xjal, _animator_xjal);    if ( _underAgents_xjal ) {      _animator_xjal.drawLink( this, connections, true, true );    }  }  public List<Object> getEmbeddedObjects() {    List<Object> list = super.getEmbeddedObjects();    if (list == null) {      list = new LinkedList<Object>();    }    list.add( hospitals );    list.add( people );    return list;  }  public AgentList<? extends Main> getPopulation() {    return (AgentList<? extends Main>) super.getPopulation();  }  public List<? extends Main> agentsInRange( double distance ) {    return (List<? extends Main>) super.agentsInRange( distance );  }  @AnyLogicInternalCodegenAPI  public void onDestroy() {    for (Agent _item : hospitals) {      _item.onDestroy();    }    for (Agent _item : people) {      _item.onDestroy();    }    map.destroy();    super.onDestroy();  }  @AnyLogicInternalCodegenAPI  @Override  public void doFinish() {    for (Agent _item : hospitals) {      _item.doFinish();    }    super.doFinish();    for (Agent _item : people) {      _item.doFinish();    }    super.doFinish();  }}package malawi_recentralization_model_2017_06_04;import java.io.Serializable;import java.sql.Connection;import java.sql.SQLException;import java.util.ArrayDeque;import java.util.ArrayList;import java.util.Arrays;import java.util.Calendar;import java.util.Collection;import java.util.Collections;import java.util.Comparator;import java.util.Currency;import java.util.Date;import java.util.Enumeration;import java.util.HashMap;import java.util.HashSet;import java.util.Hashtable;import java.util.Iterator;import java.util.LinkedHashMap;import java.util.LinkedHashSet;import java.util.LinkedList;import java.util.List;import java.util.ListIterator;import java.util.Locale;import java.util.Map;import java.util.PriorityQueue;import java.util.Random;import java.util.Set;import java.util.SortedMap;import java.util.SortedSet;import java.util.Stack;import java.util.Timer;import java.util.TreeMap;import java.util.TreeSet;import java.util.Vector;import java.awt.Color;import java.awt.Font;import java.awt.Graphics2D;import java.awt.geom.AffineTransform;import com.anylogic.engine.connectivity.ResultSet;import com.anylogic.engine.connectivity.Statement;import com.anylogic.engine.elements.*;import com.anylogic.engine.markup.Network;import com.anylogic.engine.Position;import com.anylogic.engine.markup.PedFlowStatistics;import com.anylogic.engine.markup.DensityMap;import static java.lang.Math.*;import static com.anylogic.engine.UtilitiesArray.*;import static com.anylogic.engine.UtilitiesCollection.*;import static com.anylogic.engine.presentation.UtilitiesColor.*;import static com.anylogic.engine.HyperArray.*;import com.anylogic.engine.*;import com.anylogic.engine.analysis.*;import com.anylogic.engine.connectivity.*;import com.anylogic.engine.database.*;import com.anylogic.engine.gis.*;import com.anylogic.engine.markup.*;import com.anylogic.engine.presentation.*;import com.mysema.query.Tuple;import com.mysema.query.sql.SQLBindings;import static malawi_recentralization_model_2017_06_04.DBDescriptor.*;import java.awt.geom.Arc2D;import com.mysema.query.BooleanBuilder;import com.mysema.query.types.expr.MathExpressions;import org.dom4j.IllegalAddException;import org.apache.tools.ant.filters.TokenFilter.Trim;import org.uncommons.maths.random.MersenneTwisterRNG;import org.uncommons.maths.random.GaussianGenerator;import org.apache.commons.math3.distribution.AbstractRealDistribution;import org.apache.commons.math3.distribution.NormalDistribution;import org.apache.commons.math3.distribution.LogNormalDistribution;import org.apache.commons.math3.distribution.GammaDistribution; public class Person extends Agent{  // Parameters  public double  longitude;  /**   * Returns default value for parameter <code>longitude</code>.   * <i>This method should not be called by user</i>   */  @AnyLogicInternalCodegenAPI  public double _longitude_DefaultValue_xjal() {    final Person self = this;    return 0.0;  }  public void set_longitude( double longitude ) {    if (longitude == this.longitude) {      return;    }    double _oldValue_xjal = this.longitude;    this.longitude = longitude;    onChange_longitude_xjal( _oldValue_xjal );    onChange();  }  /**   * Calls "On change" action for parameter longitude.<br>   * Note that 'oldValue' in that action will be unavailable if this method is called by user   * (current parameter value will be passed as 'oldValue').<br>   * Please call <code>set_longitude()</code> method instead.   */  protected void onChange_longitude() {    onChange_longitude_xjal( longitude );  }  @AnyLogicInternalCodegenAPI  protected void onChange_longitude_xjal( double oldValue ) {    }  public double  latitude;  /**   * Returns default value for parameter <code>latitude</code>.   * <i>This method should not be called by user</i>   */  @AnyLogicInternalCodegenAPI  public double _latitude_DefaultValue_xjal() {    final Person self = this;    return 0.0;  }  public void set_latitude( double latitude ) {    if (latitude == this.latitude) {      return;    }    double _oldValue_xjal = this.latitude;    this.latitude = latitude;    onChange_latitude_xjal( _oldValue_xjal );    onChange();  }  /**   * Calls "On change" action for parameter latitude.<br>   * Note that 'oldValue' in that action will be unavailable if this method is called by user   * (current parameter value will be passed as 'oldValue').<br>   * Please call <code>set_latitude()</code> method instead.   */  protected void onChange_latitude() {    onChange_latitude_xjal( latitude );  }  @AnyLogicInternalCodegenAPI  protected void onChange_latitude_xjal( double oldValue ) {    }  public int  wealth;  /**   * Returns default value for parameter <code>wealth</code>.   * <i>This method should not be called by user</i>   */  @AnyLogicInternalCodegenAPI  public int _wealth_DefaultValue_xjal() {    final Person self = this;    return 0;  }  public void set_wealth( int wealth ) {    if (wealth == this.wealth) {      return;    }    int _oldValue_xjal = this.wealth;    this.wealth = wealth;    onChange_wealth_xjal( _oldValue_xjal );    onChange();  }  /**   * Calls "On change" action for parameter wealth.<br>   * Note that 'oldValue' in that action will be unavailable if this method is called by user   * (current parameter value will be passed as 'oldValue').<br>   * Please call <code>set_wealth()</code> method instead.   */  protected void onChange_wealth() {    onChange_wealth_xjal( wealth );  }  @AnyLogicInternalCodegenAPI  protected void onChange_wealth_xjal( int oldValue ) {    }  public String  educ;  /**   * Returns default value for parameter <code>educ</code>.   * <i>This method should not be called by user</i>   */  @AnyLogicInternalCodegenAPI  public String _educ_DefaultValue_xjal() {    final Person self = this;    return null;  }  public void set_educ( String educ ) {    if (educ == this.educ) {      return;    }    String _oldValue_xjal = this.educ;    this.educ = educ;    onChange_educ_xjal( _oldValue_xjal );    onChange();  }  /**   * Calls "On change" action for parameter educ.<br>   * Note that 'oldValue' in that action will be unavailable if this method is called by user   * (current parameter value will be passed as 'oldValue').<br>   * Please call <code>set_educ()</code> method instead.   */  protected void onChange_educ() {    onChange_educ_xjal( educ );  }  @AnyLogicInternalCodegenAPI  protected void onChange_educ_xjal( String oldValue ) {    }  public int  age;  /**   * Returns default value for parameter <code>age</code>.   * <i>This method should not be called by user</i>   */  @AnyLogicInternalCodegenAPI  public int _age_DefaultValue_xjal() {    final Person self = this;    return 0;  }  public void set_age( int age ) {    if (age == this.age) {      return;    }    int _oldValue_xjal = this.age;    this.age = age;    onChange_age_xjal( _oldValue_xjal );    onChange();  }  /**   * Calls "On change" action for parameter age.<br>   * Note that 'oldValue' in that action will be unavailable if this method is called by user   * (current parameter value will be passed as 'oldValue').<br>   * Please call <code>set_age()</code> method instead.   */  protected void onChange_age() {    onChange_age_xjal( age );  }  @AnyLogicInternalCodegenAPI  protected void onChange_age_xjal( int oldValue ) {    }  public int  male;  /**   * Returns default value for parameter <code>male</code>.   * <i>This method should not be called by user</i>   */  @AnyLogicInternalCodegenAPI  public int _male_DefaultValue_xjal() {    final Person self = this;    return 0;  }  public void set_male( int male ) {    if (male == this.male) {      return;    }    int _oldValue_xjal = this.male;    this.male = male;    onChange_male_xjal( _oldValue_xjal );    onChange();  }  /**   * Calls "On change" action for parameter male.<br>   * Note that 'oldValue' in that action will be unavailable if this method is called by user   * (current parameter value will be passed as 'oldValue').<br>   * Please call <code>set_male()</code> method instead.   */  protected void onChange_male() {    onChange_male_xjal( male );  }  @AnyLogicInternalCodegenAPI  protected void onChange_male_xjal( int oldValue ) {    }  public double  income;  /**   * Returns default value for parameter <code>income</code>.   * <i>This method should not be called by user</i>   */  @AnyLogicInternalCodegenAPI  public double _income_DefaultValue_xjal() {    final Person self = this;    return 0.0;  }  public void set_income( double income ) {    if (income == this.income) {      return;    }    double _oldValue_xjal = this.income;    this.income = income;    onChange_income_xjal( _oldValue_xjal );    onChange();  }  /**   * Calls "On change" action for parameter income.<br>   * Note that 'oldValue' in that action will be unavailable if this method is called by user   * (current parameter value will be passed as 'oldValue').<br>   * Please call <code>set_income()</code> method instead.   */  protected void onChange_income() {    onChange_income_xjal( income );  }  @AnyLogicInternalCodegenAPI  protected void onChange_income_xjal( double oldValue ) {    }  @Override  public void setParametersToDefaultValues() {    super.setParametersToDefaultValues();    longitude = _longitude_DefaultValue_xjal();    latitude = _latitude_DefaultValue_xjal();    wealth = _wealth_DefaultValue_xjal();    educ = _educ_DefaultValue_xjal();    age = _age_DefaultValue_xjal();    male = _male_DefaultValue_xjal();    income = _income_DefaultValue_xjal();  }  @Override  public boolean setParameter(String _name_xjal, Object _value_xjal, boolean _callOnChange_xjal) {    switch ( _name_xjal ) {    case "longitude":      if ( _callOnChange_xjal ) {        set_longitude( ((Number) _value_xjal).doubleValue() );      } else {        longitude = ((Number) _value_xjal).doubleValue();      }      return true;    case "latitude":      if ( _callOnChange_xjal ) {        set_latitude( ((Number) _value_xjal).doubleValue() );      } else {        latitude = ((Number) _value_xjal).doubleValue();      }      return true;    case "wealth":      if ( _callOnChange_xjal ) {        set_wealth( ((Number) _value_xjal).intValue() );      } else {        wealth = ((Number) _value_xjal).intValue();      }      return true;    case "educ":      if ( _callOnChange_xjal ) {        set_educ( (String) _value_xjal );      } else {        educ = (String) _value_xjal;      }      return true;    case "age":      if ( _callOnChange_xjal ) {        set_age( ((Number) _value_xjal).intValue() );      } else {        age = ((Number) _value_xjal).intValue();      }      return true;    case "male":      if ( _callOnChange_xjal ) {        set_male( ((Number) _value_xjal).intValue() );      } else {        male = ((Number) _value_xjal).intValue();      }      return true;    case "income":      if ( _callOnChange_xjal ) {        set_income( ((Number) _value_xjal).doubleValue() );      } else {        income = ((Number) _value_xjal).doubleValue();      }      return true;    default:      return super.setParameter( _name_xjal, _value_xjal, _callOnChange_xjal );    }  }  @Override  public <T> T getParameter(String _name_xjal) {    Object _result_xjal;    switch ( _name_xjal ) {    case "longitude": _result_xjal = longitude; break;    case "latitude": _result_xjal = latitude; break;    case "wealth": _result_xjal = wealth; break;    case "educ": _result_xjal = educ; break;    case "age": _result_xjal = age; break;    case "male": _result_xjal = male; break;    case "income": _result_xjal = income; break;    default: _result_xjal = super.getParameter( _name_xjal ); break;    }    return (T) _result_xjal;  }  @AnyLogicInternalCodegenAPI  private static String[] _parameterNames_xjal;  @Override  public String[] getParameterNames() {    String[] result = _parameterNames_xjal;    if (result == null) {      List<String> list = new ArrayList<>( Arrays.asList( super.getParameterNames() ) );      list.add( "longitude" );      list.add( "latitude" );      list.add( "wealth" );      list.add( "educ" );      list.add( "age" );      list.add( "male" );      list.add( "income" );      result = list.toArray( new String[ list.size() ] );      _parameterNames_xjal = result;    }    return result;  }  // Plain Variables  public double  startTime;  public double  travelTime;  /**   * beta on wealth for class assignment   */  public double  bEduc;  /**   * beta on wealth for class assignment   */  public double  bMale;  /**   * beta on wealth for class assignment   */  public double  bAge;  /**   * beta on wealth for class assignment   */  public double  bDistance;  /**   * beta on wealth for class assignment   */  public double  bQ;  /**   * beta on wealth for class assignment   */  public double  bLogIncome;  /**   * beta on wealth for class assignment   */  public double  bLogIncomeSq;  /**   * beta on wealth for class assignment   */  public double  bIntercept;  /**   * beta on wealth for class assignment   */  public double  bIV;  /**   * Destination hospital   */  public Hospital  destHospital;  /**   * Destination hospital   */  public int  noCare;  // Collection Variables  public java.util.ArrayList <Hospital > provs = new java.util.ArrayList<Hospital>();  @AnyLogicInternalCodegenAPI  private static Map<String, IElementDescriptor> elementDesciptors_xjal = createElementDescriptors( Person.class );    @AnyLogicInternalCodegenAPI  @Override  public Map<String, IElementDescriptor> getElementDesciptors() {    return elementDesciptors_xjal;  }  @AnyLogicCustomProposalPriority(type = AnyLogicCustomProposalPriority.Type.STATIC_ELEMENT)  public static final Scale scale = new Scale( 10.0 );  @Override  public Scale getScale() {    return scale;  }	  // Statecharts  public Statechart<statechart_state> statechart = new Statechart<>( this, (short)1 );  @Override  @AnyLogicInternalCodegenAPI  public String getNameOf( Statechart _s ) {    if(_s == this.statechart) return "statechart";    return super.getNameOf( _s );  }  @Override  @AnyLogicInternalCodegenAPI  public void executeActionOf( Statechart _s ) {    if( _s == this.statechart ) {      enterState( WaitingToStart, true );      return;    }    super.executeActionOf( _s );  }   // States of all statecharts     public enum statechart_state implements IStatechartState<Person, statechart_state> {    WaitingToStart,    Deciding,    Traveling,    atHospital;    @AnyLogicInternalCodegenAPI    private Collection<statechart_state> _simpleStatesDeep_xjal;    @AnyLogicInternalCodegenAPI    private Set<statechart_state> _fullState_xjal;    @AnyLogicInternalCodegenAPI    private Set<statechart_state> _statesInside_xjal;    @Override    @AnyLogicInternalCodegenAPI    public Collection<statechart_state> getSimpleStatesDeep() {      Collection<statechart_state> result = _simpleStatesDeep_xjal;      if (result == null) {        _simpleStatesDeep_xjal = result = calculateAllSimpleStatesDeep();      }      return result;    }        @Override    public Set<statechart_state> getFullState() {      Set<statechart_state> result = _fullState_xjal;      if (result == null) {        _fullState_xjal = result = calculateFullState();      }      return result;    }        @Override    @AnyLogicInternalCodegenAPI    public Set<statechart_state> getStatesInside() {      Set<statechart_state> result = _statesInside_xjal;      if (result == null) {        _statesInside_xjal = result = calculateStatesInside();      }      return result;    }    @Override    @AnyLogicInternalCodegenAPI    public Statechart<statechart_state> getStatechart( Person _a ) {      return _a.statechart;    }  }  @AnyLogicCustomProposalPriority(type = AnyLogicCustomProposalPriority.Type.STATIC_ELEMENT)  public static final statechart_state WaitingToStart = statechart_state.WaitingToStart;  @AnyLogicCustomProposalPriority(type = AnyLogicCustomProposalPriority.Type.STATIC_ELEMENT)  public static final statechart_state Deciding = statechart_state.Deciding;  @AnyLogicCustomProposalPriority(type = AnyLogicCustomProposalPriority.Type.STATIC_ELEMENT)  public static final statechart_state Traveling = statechart_state.Traveling;  @AnyLogicCustomProposalPriority(type = AnyLogicCustomProposalPriority.Type.STATIC_ELEMENT)  public static final statechart_state atHospital = statechart_state.atHospital;  @AnyLogicInternalCodegenAPI  private void enterState( statechart_state _state, boolean _destination ) {    switch( _state ) {      case WaitingToStart:  	    logToDBEnterState(statechart, _state);        // (Simple state (not composite))        statechart.setActiveState_xjal( WaitingToStart );        {this.destHospital = null;Random r = new MersenneTwisterRNG();  //from uncommon maths package (found in the /Mark/Models folder)		switch (this.wealth) {	case 5:		this.circle.setFillColor(new Color(255,0,0, 40));		this.circle.setLineColor(new Color(255,0,0, 100));		break;	case 4:		this.circle.setFillColor(new Color(192,0,64, 40));		this.circle.setLineColor(new Color(192,0,64, 100));		break;	case 3:		this.circle.setFillColor(new Color(128,0,128, 40));		this.circle.setLineColor(new Color(128,0,128, 100));		break;	case 2:		this.circle.setFillColor(new Color(64,0,192, 40));		this.circle.setLineColor(new Color(64,0,192, 100));		break;	case 1:		this.circle.setFillColor(new Color(0,0,255, 40));		this.circle.setLineColor(new Color(0,0,255, 100));		break;	default: throw new IllegalAddException("No one with wealth "+ this.wealth);	}bEduc = main.educPrimaryComplete;/*	switch (this.educ) {	case "Secondary":		bEduc = new GaussianGenerator(main.educSecondaryComplete, 	main.educSecondaryCompleteSE, 	r).nextValue();		break;	case "Primary":		bEduc = new GaussianGenerator(main.educPrimaryComplete, 	main.educPrimaryCompleteSE, 	r).nextValue();		break;	case "None":		bEduc = 0.0;		break;	default: throw new IllegalAddException("No one with education "+ this.educ);	}*/bMale = new GaussianGenerator(main.male, 	main.maleSE, 	r).nextValue();bAge = 0.0;	switch (this.age) {	case 1:		bAge = new GaussianGenerator(main.age514, 	main.age514SE, 	r).nextValue();		break;	case 2:		bAge = new GaussianGenerator(main.age1521, 	main.age1521SE, 	r).nextValue();		break;	case 3:		bAge = new GaussianGenerator(main.age2249, 	main.age2249SE, 	r).nextValue();		break;	case 4:		bAge = new GaussianGenerator(main.age50, 	main.age50SE, 	r).nextValue();		break;	default: throw new IllegalAddException("No one with age "+ this.age);	}	bDistance 	= new GaussianGenerator(main.distance,	 	main.distanceSE, 	r).nextValue();bQ 			= new GaussianGenerator(main.q, 			main.qSE, 			r).nextValue();bLogIncome 	= new GaussianGenerator(main.logIncome, 	main.logIncomeSE, 	r).nextValue();bLogIncomeSq= new GaussianGenerator(main.logIncomeSq, 	main.logIncomeSqSE, r).nextValue();bIntercept	= new GaussianGenerator(main.intercept, 	main.interceptSE, 	r).nextValue();//bIV			= new GaussianGenerator(main.iv,		 	main.ivSE,		 	r).nextValue();bIV			= main.iv; //not currently allowing the IV to vary. ;}        transition2.start();        return;      case Deciding:  	    logToDBEnterState(statechart, _state);        // (Simple state (not composite))        statechart.setActiveState_xjal( Deciding );        {/**We assume nested multinomial logit, with two nests: No care, Hospital.  No care is degenerate.  The coefficient on the inclusive value (lambda[k]) is 0.442 from Ssewanyana (updated).From Train, eq. 4.4 - 4.eThe upper model—the probability of choosing nest k—is calculated aspBk = exp(Wnk + lambda[k]*Ink) / (sum over all nests (exp(Wnl + lambda[l]*Inl))where Ink, the inclusive value for each nest, is the log of the denominator of each nest:Ink = ln( sum over all choices, j, in nest k (exp(Vnj / lambda[k])Because the first nest is degenerate, its lambda is 1, and the deterministic part of its utility is assumed to be 0, meaning we can re-write the upper-level model as:pHome = 1 / (1 + exp(Wnk + 0.442 * Ink))andpHospital = exp(Wnk + 0.442 * Ink) / (1 + exp(Wnk + 0.442 * Ink))I will make the assumption that Wnk is beta * (average values of Q, distance, and cost)Calculating the lower model is a simple logit, except that lambda[k] has to be included as a correlation parameter.  So, GIVEN that a person chooses to be in a hospital:p(j|Hospital) = exp(Vj/lambda[k]) / (sum over all twigs (exp(Vt / lambda[k])))**///Add hospitals	provs.clear();	provs.addAll(main.hospitalList);//Select hospitals 	double denom = 0.0;  //lower model	double avgDist = 0.0;		for(Hospital h : provs)	{		//double cost 		= new GammaDistribution(main.phi, h.cost / main.phi).sample();  //draw a cost		//double testMean		= new GammaDistribution(main.phi, h.cost / main.phi).getNumericalMean();		//System.out.println("Sample: "+cost+"\tMean: "+ testMean + "\thCost: " + h.cost);		//cost				= max(cost - main.voucher, 0); //because decision-making is based on perceived cost, not actual cost!! *********		//cost				= cost - main.voucher; //because decision-making is based on perceived cost, not actual cost!! *********		//double cost			= h.cost - main.voucher; //*** THIS IS THE ONE YOU WANT *** testing what happens when you're allowed to pay more than the total cost. Note...not drawing from Gamma because the choice to proceed is based on perceived cost		double cost = 0.0;		//double lnConsumpt	= log(max(0.99, this.income - cost)); //assumes only a very slight negative effect if perceived / predicted cost is higher than income		//double c			= this.bLogIncome * lnConsumpt + this.bLogIncomeSq * lnConsumpt * lnConsumpt; //from p. 13 of the updated Ssewanyana (https://mpra.ub.uni-muenchen.de/8558/1/Demand_Health_Uganda_Ssewanyana_Kasirye.pdf)		//double c			= this.bLogIncome * (log(this.income) - cost / this.income) + this.bLogIncomeSq * (log(this.income)*log(this.income) - 2 * log(this.income)*(cost / this.income));  //using eqn (7) of the updated Ssewanyana (https://mpra.ub.uni-muenchen.de/8558/1/Demand_Health_Uganda_Ssewanyana_Kasirye.pdf)		double c 			= this.bLogIncome * (log(this.income) + log(1.0 - (cost / this.income)))								+ this.bLogIncomeSq * (log(this.income)*log(this.income) + 2*log(this.income)*log(1.0 - cost / this.income) + log(1.0 - cost / this.income)*log(1.0 - cost / this.income)); //Using Sahn 2003: https://onlinelibrary-wiley-com.ezp-prod1.hul.harvard.edu/doi/pdf/10.1111/1468-0084.t01-2-00046				avgDist += this.distanceByRoute(h) / 1000.0;				h.u		 	= exp((this.bIntercept + this.bMale * (double)this.male + this.bAge + this.bEduc + c + this.bDistance * this.distanceByRoute(h) / 1000.0							+ this.bQ * h.q) / this.bIV); //note distanceByRoute() returns meters		denom		+= h.u;			} //		avgDist = avgDist / (double)provs.size();		double Ink = log(denom);	double avgCost = max(main.hospitals.avgCost() - main.voucher, 0);	double avgLnConsumpt = log(max(0.99, this.income - avgCost));	double avgC = this.bLogIncome * avgLnConsumpt + this.bLogIncomeSq * avgLnConsumpt * avgLnConsumpt; 		double upperDenom = 1.0 + exp(Ink * this.bIV + this.bDistance * avgDist + this.bQ * main.hospitals.avgQ() + avgC);			if(randomFalse(1.0 / upperDenom)) //if people *don't* choose home	{ 		for(Hospital h : provs) 		{			h.p = h.u / denom;		}				double r = Math.random();		double cdf = 0.0;				for(Hospital h : provs)		{			cdf += h.p;//						System.out.print(this.getId() + "\t" + h.getId() + "\t" + this.bIntercept + "\t" + this.bMale + "\t" + this.male//								 + "\t" + this.bAge + "\t" + this.bEduc + "\t" + (max(h.cost - main.voucher, 0))//								 + "\t" + this.bLogIncome + "\t" + this.bLogIncomeSq + "\t" + this.income + "\t" + //								 (this.bLogIncome * log(max(0.99, this.income - max(h.cost - main.voucher, 0))) + this.bLogIncomeSq * log(max(0.99, this.income - max(h.cost - main.voucher, 0))) * log(max(0.99, this.income - max(h.cost - main.voucher, 0)))) //								 + "\t" + this.bDistance//								 + "\t" + (this.distanceByRoute(h)/1000.0) + "\t" + this.bQ + "\t" + h.q + "\t" + this.bIV + "\t" + h.u + "\t" + h.p//								 + "\t" + r + "\t" + cdf);			if(r <= cdf)			{  				this.destHospital = h;				main.update_hospitalAssigned();//				System.out.println("\t"+this.destHospital.getId());				break;			}//			else {System.out.println("\tnull");}		}				} else 	{		this.noCare = 1;		main.update_nNoCare();	}					 ;}        transition.start();        return;      case Traveling:  	    logToDBEnterState(statechart, _state);        // (Simple state (not composite))        statechart.setActiveState_xjal( Traveling );        {this.startTime = time(SECOND);this.moveTo(this.destHospital); ;}        transition1.start();        return;      case atHospital:  	    logToDBEnterState(statechart, _state);        // (Simple state (not composite))        statechart.setActiveState_xjal( atHospital );        {this.travelTime = (time(SECOND) - this.startTime) / 3600.0;//main.update_travelTime(); ;}        return;      default:        return;    }  }  @AnyLogicInternalCodegenAPI  private void exitState( statechart_state _state, Transition _t, boolean _source ) {    switch( _state ) {      case WaitingToStart:   	    logToDBExitState(statechart, _state);  	    logToDB(statechart, _t, _state);      // (Simple state (not composite))        if ( !_source || _t != transition2) transition2.cancel();        return;      case Deciding:   	    logToDBExitState(statechart, _state);  	    logToDB(statechart, _t, _state);      // (Simple state (not composite))        if ( !_source || _t != transition) transition.cancel();        return;      case Traveling:   	    logToDBExitState(statechart, _state);  	    logToDB(statechart, _t, _state);      // (Simple state (not composite))        if ( !_source || _t != transition1) transition1.cancel();        return;      case atHospital:   	    logToDBExitState(statechart, _state);  	    logToDB(statechart, _t, _state);      // (Simple state (not composite))        return;      default:        return;    }  }  @AnyLogicInternalCodegenAPI  private void exitInnerStates( statechart_state _destination ) {    statechart_state _state = statechart.getActiveSimpleState();    while( _state != _destination ) {			exitState( _state, null, false );			_state = _state.getContainerState();		}	}    public TransitionCondition transition2 = new TransitionCondition( this );  public TransitionCondition transition = new TransitionCondition( this );  @Override  @AnyLogicInternalCodegenAPI  public String getNameOf( TransitionCondition _t ) {    if ( _t == transition2 ) return "transition2";    if ( _t == transition ) return "transition";    return super.getNameOf( _t );  }  @Override  @AnyLogicInternalCodegenAPI  public Statechart getStatechartOf( TransitionCondition _t ) {     if ( _t == transition2 ) return statechart;    if ( _t == transition ) return statechart;    return super.getStatechartOf( _t );  }  @Override  @AnyLogicInternalCodegenAPI  public void executeActionOf( TransitionCondition _t ) {    if ( _t == transition2 ) {      exitState( WaitingToStart, _t, true );          enterState( Deciding, true );      return;    }    if ( _t == transition ) {      exitState( Deciding, _t, true );          enterState( Traveling, true );      return;    }    super.executeActionOf( _t );  }  @Override  @AnyLogicInternalCodegenAPI  public boolean testConditionOf( TransitionCondition _t ) {    if ( _t == transition2 ) return true ;    if ( _t == transition ) return //this.destHospital != nullfalse ;    return super.testConditionOf( _t );  }  public TransitionMessage transition1 = new TransitionMessage( this );  @Override  @AnyLogicInternalCodegenAPI  public String getNameOf( TransitionMessage _t ) {    if ( _t == transition1 ) return "transition1";    return super.getNameOf( _t );  }  @Override  @AnyLogicInternalCodegenAPI  public Statechart getStatechartOf( TransitionMessage _t ) {     if ( _t == transition1 ) return statechart;    return super.getStatechartOf( _t );  }  @Override  @AnyLogicInternalCodegenAPI  public void executeActionOf( TransitionMessage _t, Object _msg ) {    if ( _t == transition1 ) {      exitState( Traveling, _t, true );          enterState( atHospital, true );      return;    }    super.executeActionOf( _t, _msg );  }  @Override  @AnyLogicInternalCodegenAPI  public boolean testMessageOf( TransitionMessage _t, Object _msg ) {    if ( _t == transition1 ) {      return _msg == _ARRIVAL_message_xjal;    }    return super.testMessageOf( _t, _msg );  }  // View areas  public ViewArea _origin_VA = new ViewArea( this, "[Origin]", 0, 0, 1550.0, 890.0 );  @Override  @AnyLogicInternalCodegenAPI  public int getViewAreas(Map<String, ViewArea> _output) {    if ( _output != null ) {      _output.put( "_origin_VA", this._origin_VA );    }    return 1 + super.getViewAreas( _output );  }  protected static final Color _circle_Fill_Color = new Color( 0x1DFF00FF, true );  @AnyLogicInternalCodegenAPI  protected static final int _circle = 1;  /** Internal constant, shouldn't be accessed by user */  @AnyLogicInternalCodegenAPI  protected static final int _SHAPE_NEXT_ID_xjal = 2;   /**   * Top-level presentation group id   */	   @AnyLogicInternalCodegenAPI  protected static final int _presentation = 0;  @AnyLogicInternalCodegenAPI  public boolean isPublicPresentationDefined() {    return true;  }  @AnyLogicInternalCodegenAPI  public boolean isEmbeddedAgentPresentationVisible( Agent _a ) {    return super.isEmbeddedAgentPresentationVisible( _a );  }  /**   * Top-level icon group id   */	   @AnyLogicInternalCodegenAPI  protected static final int _icon = -1;    @Override  @AnyLogicInternalCodegenAPI  public boolean onShapeClick( int _shape, int index, double clickx, double clicky ){    switch( _shape ){      case _circle:        if (true) {          ShapeOval self = this.circle;          main.vAgentID = this.getLatitude();         }        break;      default: return super.onShapeClick( _shape, index, clickx, clicky ); 	} 	return false;  }      protected ShapeOval circle;  @AnyLogicInternalCodegenAPI  private void _createPersistentElementsBP0_xjal() {    circle = new ShapeOval(       SHAPE_DRAW_2D3D, true,0.0, 0.0, 0.0, 0.0,        purple, _circle_Fill_Color,	   2.0, 2.0, 0.0, 1.0, LINE_STYLE_SOLID ) {      @Override      @AnyLogicInternalCodegenAPI      public boolean onClick( double clickx, double clicky ) {        return onShapeClick( _circle, 0, clickx, clicky );      }    };  }  @AnyLogicInternalCodegenAPI  private void _createPersistentElementsAP0_xjal() {  }  // Static initialization of persistent elements  {    _createPersistentElementsBP0_xjal();  }  protected ShapeTopLevelPresentationGroup presentation;  protected ShapeModelElementsGroup icon;   @Override  @AnyLogicInternalCodegenAPI  public ShapeTopLevelPresentationGroup getPresentationShape() {    return presentation;  }  @Override  @AnyLogicInternalCodegenAPI  public ShapeModelElementsGroup getModelElementsShape() {    return icon;  }  @Override  @AnyLogicInternalCodegenAPI  public Object getPersistentShape( int _shape ) {    switch (_shape) {      case _presentation: return presentation;      case _icon: return icon;      case _circle: return circle;      default: return super.getPersistentShape( _shape );     }  }  @Override  @AnyLogicInternalCodegenAPI  public String getNameOfShape_xjal( Object _shape ) {    try {      if ( _shape == null ) return null;      String _name_xjal;      _name_xjal = checkNameOfShape_xjal( _shape, presentation, "presentation" ); if (_name_xjal != null) return _name_xjal;      _name_xjal = checkNameOfShape_xjal( _shape, icon, "icon" ); if (_name_xjal != null) return _name_xjal;      _name_xjal = checkNameOfShape_xjal( _shape, circle, "circle" ); if (_name_xjal != null) return _name_xjal;    } catch (Exception e) {      return null;    }    return super.getNameOfShape_xjal( _shape );  }  @AnyLogicInternalCodegenAPI  protected static final int[] _transition2_pointsX_xjal = {310, 310, };  @AnyLogicInternalCodegenAPI  protected static final int[] _transition2_pointsY_xjal = {90, 140, };  @AnyLogicInternalCodegenAPI  protected static final int[] _transition_pointsX_xjal = {310, 310, };  @AnyLogicInternalCodegenAPI  protected static final int[] _transition_pointsY_xjal = {170, 220, };  @AnyLogicInternalCodegenAPI  protected static final int[] _transition1_pointsX_xjal = {310, 310, };  @AnyLogicInternalCodegenAPI  protected static final int[] _transition1_pointsY_xjal = {250, 300, };  @AnyLogicInternalCodegenAPI  private void drawModelElements_Statecharts_xjal(Panel _panel, Graphics2D _g, boolean _publicOnly, boolean _isSuperClass ) {    if (!_publicOnly) {      drawState( _panel, _g, 260, 220, 100, 30, 10, 10, "Traveling", GOLD, Traveling, statechart );    }    if (!_publicOnly) {      drawState( _panel, _g, 260, 300, 100, 30, 10, 10, "atHospital", GOLD, atHospital, statechart );    }    if (!_publicOnly) {      drawState( _panel, _g, 260, 60, 100, 30, 10, 10, "WaitingToStart", GOLD, WaitingToStart, statechart );    }    if (!_publicOnly) {      drawState( _panel, _g, 260, 140, 110, 30, 10, 10, "Deciding", GOLD, Deciding, statechart );    }    if (!_publicOnly) {      drawStatechartEntryPoint( _panel, _g, 310, 20, 310, 60, 320, 20, "statechart",  statechart );    }    if (!_publicOnly) {      drawTransition(_panel, _g, _transition_pointsX_xjal, _transition_pointsY_xjal, 320, 170, null, transition, TransitionIcon.CONDITION, 310, 190 );    }    if (!_publicOnly) {      drawTransition(_panel, _g, _transition1_pointsX_xjal, _transition1_pointsY_xjal, 320, 250, null, transition1, TransitionIcon.ARRIVAL, 310, 270 );    }    if (!_publicOnly) {      drawTransition(_panel, _g, _transition2_pointsX_xjal, _transition2_pointsY_xjal, 320, 90, null, transition2, TransitionIcon.CONDITION, 310, 110 );    }  }  @AnyLogicInternalCodegenAPI  private void drawModelElements_Parameters_xjal(Panel _panel, Graphics2D _g, boolean _publicOnly, boolean _isSuperClass ) {    if (!_publicOnly) {      drawParameter( _panel, _g, 50, 50, 10, 0, "longitude", longitude, 0 );    }    if (!_publicOnly) {      drawParameter( _panel, _g, 50, 100, 10, 0, "latitude", latitude, 0 );    }    if (!_publicOnly) {      drawParameter( _panel, _g, 50, 150, 10, 0, "wealth", wealth, 0 );    }    if (!_publicOnly) {      drawParameter( _panel, _g, 50, 200, 10, 0, "educ", educ, 0 );    }    if (!_publicOnly) {      drawParameter( _panel, _g, 50, 300, 10, 0, "age", age, 0 );    }    if (!_publicOnly) {      drawParameter( _panel, _g, 50, 250, 10, 0, "male", male, 0 );    }    if (!_publicOnly) {      drawParameter( _panel, _g, 50, 350, 10, 0, "income", income, 0 );    }  }  @AnyLogicInternalCodegenAPI  private void drawModelElements_PlainVariables_xjal(Panel _panel, Graphics2D _g, boolean _publicOnly, boolean _isSuperClass ) {    if (!_publicOnly) {      drawPlainVariable( _panel, _g, 150, 150, 10, 0, "startTime", startTime, false );    }    if (!_publicOnly) {      drawPlainVariable( _panel, _g, 150, 200, 10, 0, "travelTime", travelTime, false );    }    if (!_publicOnly) {      drawPlainVariable( _panel, _g, 50, 400, 10, 0, "bEduc", bEduc, false );    }    if (!_publicOnly) {      drawPlainVariable( _panel, _g, 50, 450, 10, 0, "bMale", bMale, false );    }    if (!_publicOnly) {      drawPlainVariable( _panel, _g, 50, 500, 10, 0, "bAge", bAge, false );    }    if (!_publicOnly) {      drawPlainVariable( _panel, _g, 150, 400, 10, 0, "bDistance", bDistance, false );    }    if (!_publicOnly) {      drawPlainVariable( _panel, _g, 150, 450, 10, 0, "bQ", bQ, false );    }    if (!_publicOnly) {      drawPlainVariable( _panel, _g, 150, 500, 10, 0, "bLogIncome", bLogIncome, false );    }    if (!_publicOnly) {      drawPlainVariable( _panel, _g, 150, 550, 10, 0, "bLogIncomeSq", bLogIncomeSq, false );    }    if (!_publicOnly) {      drawPlainVariable( _panel, _g, 50, 600, 10, 0, "bIntercept", bIntercept, false );    }    if (!_publicOnly) {      drawPlainVariable( _panel, _g, 50, 550, 10, 0, "bIV", bIV, false );    }    if (!_publicOnly) {      drawPlainVariable( _panel, _g, 150, 50, 10, 0, "destHospital", destHospital, false );    }    if (!_publicOnly) {      drawPlainVariable( _panel, _g, 150, 100, 10, 0, "noCare", noCare, false );    }  }  @AnyLogicInternalCodegenAPI  private void drawModelElements_CollectionVariables_xjal(Panel _panel, Graphics2D _g, boolean _publicOnly, boolean _isSuperClass ) {    if (!_publicOnly) {      drawCollection( _panel, _g, 150, 250, 10, 0, "provs", provs );    }  }  @AnyLogicInternalCodegenAPI  private void drawModelElements_AgentLinks_xjal(Panel _panel, Graphics2D _g, boolean _publicOnly, boolean _isSuperClass ) {    if (_publicOnly) { return; }      drawLinkToContainer( _panel, _g, 50, -100, 10, 0, "main", main );      drawLinkToAgent( _panel, _g, 50, -50, 15, 0, "connections", true, connections );  }  @Override  @AnyLogicInternalCodegenAPI  public void drawModelElements( Panel _panel, Graphics2D _g, boolean _publicOnly, boolean _isSuperClass ) {  	super.drawModelElements( _panel, _g, _publicOnly, true );    drawModelElements_Statecharts_xjal( _panel, _g, _publicOnly, _isSuperClass );    drawModelElements_Parameters_xjal( _panel, _g, _publicOnly, _isSuperClass );    drawModelElements_PlainVariables_xjal( _panel, _g, _publicOnly, _isSuperClass );    drawModelElements_CollectionVariables_xjal( _panel, _g, _publicOnly, _isSuperClass );    drawModelElements_AgentLinks_xjal( _panel, _g, _publicOnly, _isSuperClass );  }  @AnyLogicInternalCodegenAPI  private boolean onClickModelAt_AgentLinks_xjal( Panel _panel, double _x, double _y, int _clickCount, boolean _publicOnly, boolean _isSuperClass ) {    if ( modelElementContains(_x, _y, 50, -100) ) {      if ( _clickCount == 2 ) {        _panel.browseAgent_xjal( 50, -100, this, "main" );      } else {        _panel.addInspect( 50, -100, this, "main" );      }      return true;    }    if ( modelElementContains(_x, _y, 50, -50) ) {        _panel.addInspect_xjal( 50, -50, this, "connections", Panel.INSPECT_CONNECTIONS_xjal );      return true;    }    return false;  }  @AnyLogicInternalCodegenAPI  private boolean onClickModelAt_Parameters_xjal( Panel _panel, double _x, double _y, int _clickCount, boolean _publicOnly, boolean _isSuperClass ) {    if( !_publicOnly && modelElementContains(_x, _y, 50, 50) ) {      _panel.addInspect( 50, 50, this, "longitude" );       return true;    }    if( !_publicOnly && modelElementContains(_x, _y, 50, 100) ) {      _panel.addInspect( 50, 100, this, "latitude" );       return true;    }    if( !_publicOnly && modelElementContains(_x, _y, 50, 150) ) {      _panel.addInspect( 50, 150, this, "wealth" );       return true;    }    if( !_publicOnly && modelElementContains(_x, _y, 50, 200) ) {      _panel.addInspect( 50, 200, this, "educ" );       return true;    }    if( !_publicOnly && modelElementContains(_x, _y, 50, 300) ) {      _panel.addInspect( 50, 300, this, "age" );       return true;    }    if( !_publicOnly && modelElementContains(_x, _y, 50, 250) ) {      _panel.addInspect( 50, 250, this, "male" );       return true;    }    if( !_publicOnly && modelElementContains(_x, _y, 50, 350) ) {      _panel.addInspect( 50, 350, this, "income" );       return true;    }    return false;  }  @AnyLogicInternalCodegenAPI  private boolean onClickModelAt_PlainVariables_xjal( Panel _panel, double _x, double _y, int _clickCount, boolean _publicOnly, boolean _isSuperClass ) {    if( !_publicOnly && modelElementContains(_x, _y, 150, 150) ) {      _panel.addInspect( 150, 150, this, "startTime" );       return true;    }    if( !_publicOnly && modelElementContains(_x, _y, 150, 200) ) {      _panel.addInspect( 150, 200, this, "travelTime" );       return true;    }    if( !_publicOnly && modelElementContains(_x, _y, 50, 400) ) {      _panel.addInspect( 50, 400, this, "bEduc" );       return true;    }    if( !_publicOnly && modelElementContains(_x, _y, 50, 450) ) {      _panel.addInspect( 50, 450, this, "bMale" );       return true;    }    if( !_publicOnly && modelElementContains(_x, _y, 50, 500) ) {      _panel.addInspect( 50, 500, this, "bAge" );       return true;    }    if( !_publicOnly && modelElementContains(_x, _y, 150, 400) ) {      _panel.addInspect( 150, 400, this, "bDistance" );       return true;    }    if( !_publicOnly && modelElementContains(_x, _y, 150, 450) ) {      _panel.addInspect( 150, 450, this, "bQ" );       return true;    }    if( !_publicOnly && modelElementContains(_x, _y, 150, 500) ) {      _panel.addInspect( 150, 500, this, "bLogIncome" );       return true;    }    if( !_publicOnly && modelElementContains(_x, _y, 150, 550) ) {      _panel.addInspect( 150, 550, this, "bLogIncomeSq" );       return true;    }    if( !_publicOnly && modelElementContains(_x, _y, 50, 600) ) {      _panel.addInspect( 50, 600, this, "bIntercept" );       return true;    }    if( !_publicOnly && modelElementContains(_x, _y, 50, 550) ) {      _panel.addInspect( 50, 550, this, "bIV" );       return true;    }    if( !_publicOnly && modelElementContains(_x, _y, 150, 50) ) {      _panel.addInspect( 150, 50, this, "destHospital" );       return true;    }    if( !_publicOnly && modelElementContains(_x, _y, 150, 100) ) {      _panel.addInspect( 150, 100, this, "noCare" );       return true;    }    return false;  }  @AnyLogicInternalCodegenAPI  private boolean onClickModelAt_CollectionVariables_xjal( Panel _panel, double _x, double _y, int _clickCount, boolean _publicOnly, boolean _isSuperClass ) {    if( !_publicOnly && modelElementContains(_x, _y, 150, 250) ) {      _panel.addInspect( 150, 250, this, "provs" );       return true;    }    return false;  }  @Override  @AnyLogicInternalCodegenAPI  public boolean onClickModelAt( Panel _panel, double _x, double _y, int _clickCount, boolean _publicOnly, boolean _isSuperClass ) {    if ( onClickModelAt_AgentLinks_xjal( _panel, _x, _y, _clickCount, _publicOnly, _isSuperClass ) ) { return true; }    if ( onClickModelAt_Parameters_xjal( _panel, _x, _y, _clickCount, _publicOnly, _isSuperClass ) ) { return true; }    if ( onClickModelAt_PlainVariables_xjal( _panel, _x, _y, _clickCount, _publicOnly, _isSuperClass ) ) { return true; }    if ( onClickModelAt_CollectionVariables_xjal( _panel, _x, _y, _clickCount, _publicOnly, _isSuperClass ) ) { return true; }    return super.onClickModelAt( _panel, _x, _y, _clickCount, _publicOnly, true );  }  @Override  @AnyLogicInternalCodegenAPI  public void onArrival() {    super.onArrival();    statechart.fireEvent( _ARRIVAL_message_xjal );  }  /**   * Constructor   */  public Person( Engine engine, Agent owner, AgentList<? extends Person> ownerPopulation ) {    super( engine, owner, ownerPopulation );    instantiateBaseStructureThis_xjal();  }  @AnyLogicInternalCodegenAPI  public void onOwnerChanged_xjal() {    super.onOwnerChanged_xjal();    setupReferences_xjal();  }  @AnyLogicInternalCodegenAPI  public void instantiateBaseStructure_xjal() {    super.instantiateBaseStructure_xjal();    instantiateBaseStructureThis_xjal();  }    @AnyLogicInternalCodegenAPI  private void instantiateBaseStructureThis_xjal() {    setupReferences_xjal();  }    @AnyLogicInternalCodegenAPI  private void setupReferences_xjal() {    main = get_Main();  }    /**   * Simple constructor. Please add created agent to some population by calling goToPopulation() function   */  public Person() {  }    /**   * Simple constructor. Please add created agent to some population by calling goToPopulation() function   */  public Person( double longitude, double latitude, int wealth, String educ, int age, int male, double income ) {    markParametersAreSet();    this.longitude = longitude;    this.latitude = latitude;    this.wealth = wealth;    this.educ = educ;    this.age = age;    this.male = male;    this.income = income;  }    @Override  @AnyLogicInternalCodegenAPI  public void doCreate() {    super.doCreate();    // Assigning initial values for plain variables    setupPlainVariables_Person_xjal();    // Dynamic initialization of persistent elements    _createPersistentElementsAP0_xjal();    presentation = new ShapeTopLevelPresentationGroup( Person.this, true, 0, 0, 0, 0 , circle );    icon = new ShapeModelElementsGroup( Person.this, getElementProperty( "malawi_recentralization_model_2017_06_04.Person.icon", IElementDescriptor.MODEL_ELEMENT_DESCRIPTORS )  );    icon.setIconOffsets( 0.0, 0.0 );	 // Port connectors with non-replicated objects    // Creating replicated embedded objects    setupInitialConditions_xjal( Person.class );  }  @AnyLogicInternalCodegenAPI  public void setupExt_xjal(AgentExtension _ext) {    // Agent properties setup    if ( _ext instanceof ExtAgentWithSpatialMetrics && _ext instanceof ExtWithSpaceType ) {      double _value;      _value = 30 ;      ((ExtAgentWithSpatialMetrics) _ext).setSpeed( _value, KPH );    }  }  @Override  @AnyLogicInternalCodegenAPI  public void doStart() {    super.doStart();    statechart.start();  }   /**   * Assigning initial values for plain variables<br>   * <em>This method isn't designed to be called by user and may be removed in future releases.</em>   */  @AnyLogicInternalCodegenAPI  public void setupPlainVariables_xjal() {    setupPlainVariables_Person_xjal();  }  /**   * Assigning initial values for plain variables<br>   * <em>This method isn't designed to be called by user and may be removed in future releases.</em>   */  @AnyLogicInternalCodegenAPI  private void setupPlainVariables_Person_xjal() {    destHospital = null ;    noCare = 0 ;  }  // User API -----------------------------------------------------  public Main get_Main() {    {      Agent owner = getOwner();      if ( owner instanceof Main ) return (Main) owner;    }    return null;  }  /**   * Read-only variable. <em>Shouldn't be modified by user.</em>   */  @AnyLogicCustomSerialization(AnyLogicCustomSerializationMode.REFERENCE)  public transient malawi_recentralization_model_2017_06_04.Main main;  @AnyLogicInternalCodegenAPI  static LinkToAgentAnimationSettings _connections_commonAnimationSettings_xjal = new LinkToAgentAnimationSettingsImpl( false, black, 1.0, LINE_STYLE_SOLID, ARROW_NONE, 0.0 );  public LinkToAgentCollection<Agent, Agent> connections = new LinkToAgentStandardImpl<Agent, Agent>(this, _connections_commonAnimationSettings_xjal);  @Override  public LinkToAgentCollection<? extends Agent, ? extends Agent> getLinkToAgentStandard_xjal() {    return connections;  }  @Override  @AnyLogicInternalCodegenAPI  public void onReceive( Object _msg_xjal, Agent _sender_xjal ) {    super.onReceive( _msg_xjal, _sender_xjal );    statechart.fireEvent( _msg_xjal );  }  @AnyLogicInternalCodegenAPI  public void drawLinksToAgents(boolean _underAgents_xjal, LinkToAgentAnimator _animator_xjal) {    super.drawLinksToAgents(_underAgents_xjal, _animator_xjal);    if ( _underAgents_xjal ) {      _animator_xjal.drawLink( this, connections, true, true );    }  }  public AgentList<? extends Person> getPopulation() {    return (AgentList<? extends Person>) super.getPopulation();  }  public List<? extends Person> agentsInRange( double distance ) {    return (List<? extends Person>) super.agentsInRange( distance );  }  // Reaction on changes -------------------------------------  public void onChange() {    super.onChange();    statechart.onChange();  }  @AnyLogicInternalCodegenAPI  public void onDestroy() {    statechart.onDestroy();    super.onDestroy();  }}package malawi_recentralization_model_2017_06_04;import java.io.Serializable;import java.sql.Connection;import java.sql.SQLException;import java.util.ArrayDeque;import java.util.ArrayList;import java.util.Arrays;import java.util.Calendar;import java.util.Collection;import java.util.Collections;import java.util.Comparator;import java.util.Currency;import java.util.Date;import java.util.Enumeration;import java.util.HashMap;import java.util.HashSet;import java.util.Hashtable;import java.util.Iterator;import java.util.LinkedHashMap;import java.util.LinkedHashSet;import java.util.LinkedList;import java.util.List;import java.util.ListIterator;import java.util.Locale;import java.util.Map;import java.util.PriorityQueue;import java.util.Random;import java.util.Set;import java.util.SortedMap;import java.util.SortedSet;import java.util.Stack;import java.util.Timer;import java.util.TreeMap;import java.util.TreeSet;import java.util.Vector;import java.awt.Color;import java.awt.Font;import java.awt.Graphics2D;import java.awt.geom.AffineTransform;import com.anylogic.engine.connectivity.ResultSet;import com.anylogic.engine.connectivity.Statement;import com.anylogic.engine.elements.*;import com.anylogic.engine.markup.Network;import com.anylogic.engine.Position;import com.anylogic.engine.markup.PedFlowStatistics;import com.anylogic.engine.markup.DensityMap;import static java.lang.Math.*;import static com.anylogic.engine.UtilitiesArray.*;import static com.anylogic.engine.UtilitiesCollection.*;import static com.anylogic.engine.presentation.UtilitiesColor.*;import static com.anylogic.engine.HyperArray.*;import com.anylogic.engine.*;import com.anylogic.engine.analysis.*;import com.anylogic.engine.connectivity.*;import com.anylogic.engine.database.*;import com.anylogic.engine.gis.*;import com.anylogic.engine.markup.*;import com.anylogic.engine.presentation.*;import com.mysema.query.Tuple;import com.mysema.query.sql.SQLBindings;import static malawi_recentralization_model_2017_06_04.DBDescriptor.*;import java.awt.geom.Arc2D;public class Hospital extends Agent{  // Parameters  public String  id;  /**   * Returns default value for parameter <code>id</code>.   * <i>This method should not be called by user</i>   */  @AnyLogicInternalCodegenAPI  public String _id_DefaultValue_xjal() {    final Hospital self = this;    return null;  }  public void set_id( String id ) {    if (id == this.id) {      return;    }    String _oldValue_xjal = this.id;    this.id = id;    onChange_id_xjal( _oldValue_xjal );    onChange();  }  /**   * Calls "On change" action for parameter id.<br>   * Note that 'oldValue' in that action will be unavailable if this method is called by user   * (current parameter value will be passed as 'oldValue').<br>   * Please call <code>set_id()</code> method instead.   */  protected void onChange_id() {    onChange_id_xjal( id );  }  @AnyLogicInternalCodegenAPI  protected void onChange_id_xjal( String oldValue ) {    }  public double  latitude;  /**   * Returns default value for parameter <code>latitude</code>.   * <i>This method should not be called by user</i>   */  @AnyLogicInternalCodegenAPI  public double _latitude_DefaultValue_xjal() {    final Hospital self = this;    return 0.0;  }  public void set_latitude( double latitude ) {    if (latitude == this.latitude) {      return;    }    double _oldValue_xjal = this.latitude;    this.latitude = latitude;    onChange_latitude_xjal( _oldValue_xjal );    onChange();  }  /**   * Calls "On change" action for parameter latitude.<br>   * Note that 'oldValue' in that action will be unavailable if this method is called by user   * (current parameter value will be passed as 'oldValue').<br>   * Please call <code>set_latitude()</code> method instead.   */  protected void onChange_latitude() {    onChange_latitude_xjal( latitude );  }  @AnyLogicInternalCodegenAPI  protected void onChange_latitude_xjal( double oldValue ) {    }  public double  longitude;  /**   * Returns default value for parameter <code>longitude</code>.   * <i>This method should not be called by user</i>   */  @AnyLogicInternalCodegenAPI  public double _longitude_DefaultValue_xjal() {    final Hospital self = this;    return 0.0;  }  public void set_longitude( double longitude ) {    if (longitude == this.longitude) {      return;    }    double _oldValue_xjal = this.longitude;    this.longitude = longitude;    onChange_longitude_xjal( _oldValue_xjal );    onChange();  }  /**   * Calls "On change" action for parameter longitude.<br>   * Note that 'oldValue' in that action will be unavailable if this method is called by user   * (current parameter value will be passed as 'oldValue').<br>   * Please call <code>set_longitude()</code> method instead.   */  protected void onChange_longitude() {    onChange_longitude_xjal( longitude );  }  @AnyLogicInternalCodegenAPI  protected void onChange_longitude_xjal( double oldValue ) {    }  public double  q;  /**   * Returns default value for parameter <code>q</code>.   * <i>This method should not be called by user</i>   */  @AnyLogicInternalCodegenAPI  public double _q_DefaultValue_xjal() {    final Hospital self = this;    return 0.0;  }  public void set_q( double q ) {    if (q == this.q) {      return;    }    double _oldValue_xjal = this.q;    this.q = q;    onChange_q_xjal( _oldValue_xjal );    onChange();  }  /**   * Calls "On change" action for parameter q.<br>   * Note that 'oldValue' in that action will be unavailable if this method is called by user   * (current parameter value will be passed as 'oldValue').<br>   * Please call <code>set_q()</code> method instead.   */  protected void onChange_q() {    onChange_q_xjal( q );  }  @AnyLogicInternalCodegenAPI  protected void onChange_q_xjal( double oldValue ) {    }  public double  cost;  /**   * Returns default value for parameter <code>cost</code>.   * <i>This method should not be called by user</i>   */  @AnyLogicInternalCodegenAPI  public double _cost_DefaultValue_xjal() {    final Hospital self = this;    return 0.0;  }  public void set_cost( double cost ) {    if (cost == this.cost) {      return;    }    double _oldValue_xjal = this.cost;    this.cost = cost;    onChange_cost_xjal( _oldValue_xjal );    onChange();  }  /**   * Calls "On change" action for parameter cost.<br>   * Note that 'oldValue' in that action will be unavailable if this method is called by user   * (current parameter value will be passed as 'oldValue').<br>   * Please call <code>set_cost()</code> method instead.   */  protected void onChange_cost() {    onChange_cost_xjal( cost );  }  @AnyLogicInternalCodegenAPI  protected void onChange_cost_xjal( double oldValue ) {    }  @Override  public void setParametersToDefaultValues() {    super.setParametersToDefaultValues();    id = _id_DefaultValue_xjal();    latitude = _latitude_DefaultValue_xjal();    longitude = _longitude_DefaultValue_xjal();    q = _q_DefaultValue_xjal();    cost = _cost_DefaultValue_xjal();  }  @Override  public boolean setParameter(String _name_xjal, Object _value_xjal, boolean _callOnChange_xjal) {    switch ( _name_xjal ) {    case "id":      if ( _callOnChange_xjal ) {        set_id( (String) _value_xjal );      } else {        id = (String) _value_xjal;      }      return true;    case "latitude":      if ( _callOnChange_xjal ) {        set_latitude( ((Number) _value_xjal).doubleValue() );      } else {        latitude = ((Number) _value_xjal).doubleValue();      }      return true;    case "longitude":      if ( _callOnChange_xjal ) {        set_longitude( ((Number) _value_xjal).doubleValue() );      } else {        longitude = ((Number) _value_xjal).doubleValue();      }      return true;    case "q":      if ( _callOnChange_xjal ) {        set_q( ((Number) _value_xjal).doubleValue() );      } else {        q = ((Number) _value_xjal).doubleValue();      }      return true;    case "cost":      if ( _callOnChange_xjal ) {        set_cost( ((Number) _value_xjal).doubleValue() );      } else {        cost = ((Number) _value_xjal).doubleValue();      }      return true;    default:      return super.setParameter( _name_xjal, _value_xjal, _callOnChange_xjal );    }  }  @Override  public <T> T getParameter(String _name_xjal) {    Object _result_xjal;    switch ( _name_xjal ) {    case "id": _result_xjal = id; break;    case "latitude": _result_xjal = latitude; break;    case "longitude": _result_xjal = longitude; break;    case "q": _result_xjal = q; break;    case "cost": _result_xjal = cost; break;    default: _result_xjal = super.getParameter( _name_xjal ); break;    }    return (T) _result_xjal;  }  @AnyLogicInternalCodegenAPI  private static String[] _parameterNames_xjal;  @Override  public String[] getParameterNames() {    String[] result = _parameterNames_xjal;    if (result == null) {      List<String> list = new ArrayList<>( Arrays.asList( super.getParameterNames() ) );      list.add( "id" );      list.add( "latitude" );      list.add( "longitude" );      list.add( "q" );      list.add( "cost" );      result = list.toArray( new String[ list.size() ] );      _parameterNames_xjal = result;    }    return result;  }  // Plain Variables  public double  u;  public double  p;  /**   * is the voucher bigger than the hospital's cost?   */  public boolean  vTooBig;  @AnyLogicInternalCodegenAPI  private static Map<String, IElementDescriptor> elementDesciptors_xjal = createElementDescriptors( Hospital.class );    @AnyLogicInternalCodegenAPI  @Override  public Map<String, IElementDescriptor> getElementDesciptors() {    return elementDesciptors_xjal;  }  @AnyLogicCustomProposalPriority(type = AnyLogicCustomProposalPriority.Type.STATIC_ELEMENT)  public static final Scale scale = new Scale( 10.0 );  @Override  public Scale getScale() {    return scale;  }	  // View areas  public ViewArea _origin_VA = new ViewArea( this, "[Origin]", 0, 0, 1550.0, 890.0 );  @Override  @AnyLogicInternalCodegenAPI  public int getViewAreas(Map<String, ViewArea> _output) {    if ( _output != null ) {      _output.put( "_origin_VA", this._origin_VA );    }    return 1 + super.getViewAreas( _output );  }  @AnyLogicInternalCodegenAPI  protected static final int _rectangle = 1;  /** Internal constant, shouldn't be accessed by user */  @AnyLogicInternalCodegenAPI  protected static final int _SHAPE_NEXT_ID_xjal = 2;   /**   * Top-level presentation group id   */	   @AnyLogicInternalCodegenAPI  protected static final int _presentation = 0;  @AnyLogicInternalCodegenAPI  public boolean isPublicPresentationDefined() {    return true;  }  @AnyLogicInternalCodegenAPI  public boolean isEmbeddedAgentPresentationVisible( Agent _a ) {    return super.isEmbeddedAgentPresentationVisible( _a );  }  /**   * Top-level icon group id   */	   @AnyLogicInternalCodegenAPI  protected static final int _icon = -1;    @Override  @AnyLogicInternalCodegenAPI  public boolean onShapeClick( int _shape, int index, double clickx, double clicky ){    switch( _shape ){      case _rectangle:        if (true) {          ShapeRectangle self = this.rectangle;          main.vAgentID = this.getLatitude();         }        break;      default: return super.onShapeClick( _shape, index, clickx, clicky ); 	} 	return false;  }      protected ShapeRectangle rectangle;  @AnyLogicInternalCodegenAPI  private void _createPersistentElementsBP0_xjal() {    rectangle = new ShapeRectangle(       SHAPE_DRAW_2D3D, true,-5.0, -5.0, 0.0, 0.0,             black, black,			6.0, 6.0, 0.0, 1.0, LINE_STYLE_SOLID ) {      @Override      @AnyLogicInternalCodegenAPI      public boolean onClick( double clickx, double clicky ) {        return onShapeClick( _rectangle, 0, clickx, clicky );      }    };  }  @AnyLogicInternalCodegenAPI  private void _createPersistentElementsAP0_xjal() {  }  // Static initialization of persistent elements  {    _createPersistentElementsBP0_xjal();  }  protected ShapeTopLevelPresentationGroup presentation;  protected ShapeModelElementsGroup icon;   @Override  @AnyLogicInternalCodegenAPI  public ShapeTopLevelPresentationGroup getPresentationShape() {    return presentation;  }  @Override  @AnyLogicInternalCodegenAPI  public ShapeModelElementsGroup getModelElementsShape() {    return icon;  }  @Override  @AnyLogicInternalCodegenAPI  public Object getPersistentShape( int _shape ) {    switch (_shape) {      case _presentation: return presentation;      case _icon: return icon;      case _rectangle: return rectangle;      default: return super.getPersistentShape( _shape );     }  }  @Override  @AnyLogicInternalCodegenAPI  public String getNameOfShape_xjal( Object _shape ) {    try {      if ( _shape == null ) return null;      String _name_xjal;      _name_xjal = checkNameOfShape_xjal( _shape, presentation, "presentation" ); if (_name_xjal != null) return _name_xjal;      _name_xjal = checkNameOfShape_xjal( _shape, icon, "icon" ); if (_name_xjal != null) return _name_xjal;      _name_xjal = checkNameOfShape_xjal( _shape, rectangle, "rectangle" ); if (_name_xjal != null) return _name_xjal;    } catch (Exception e) {      return null;    }    return super.getNameOfShape_xjal( _shape );  }  @AnyLogicInternalCodegenAPI  private void drawModelElements_Parameters_xjal(Panel _panel, Graphics2D _g, boolean _publicOnly, boolean _isSuperClass ) {    if (!_publicOnly) {      drawParameter( _panel, _g, 50, 50, 10, 0, "id", id, 0 );    }    if (!_publicOnly) {      drawParameter( _panel, _g, 50, 100, 10, 0, "latitude", latitude, 0 );    }    if (!_publicOnly) {      drawParameter( _panel, _g, 50, 150, 10, 0, "longitude", longitude, 0 );    }    if (!_publicOnly) {      drawParameter( _panel, _g, 50, 200, 10, 0, "q", q, 0 );    }    if (!_publicOnly) {      drawParameter( _panel, _g, 50, 250, 10, 0, "cost", cost, 0 );    }  }  @AnyLogicInternalCodegenAPI  private void drawModelElements_PlainVariables_xjal(Panel _panel, Graphics2D _g, boolean _publicOnly, boolean _isSuperClass ) {    if (!_publicOnly) {      drawPlainVariable( _panel, _g, 150, 50, 10, 0, "u", u, false );    }    if (!_publicOnly) {      drawPlainVariable( _panel, _g, 150, 100, 10, 0, "p", p, false );    }    if (!_publicOnly) {      drawPlainVariable( _panel, _g, 150, 150, 10, 0, "vTooBig", vTooBig, false );    }  }  @AnyLogicInternalCodegenAPI  private void drawModelElements_AgentLinks_xjal(Panel _panel, Graphics2D _g, boolean _publicOnly, boolean _isSuperClass ) {    if (_publicOnly) { return; }      drawLinkToContainer( _panel, _g, 50, -100, 10, 0, "main", main );      drawLinkToAgent( _panel, _g, 50, -50, 15, 0, "connections", true, connections );  }  @Override  @AnyLogicInternalCodegenAPI  public void drawModelElements( Panel _panel, Graphics2D _g, boolean _publicOnly, boolean _isSuperClass ) {  	super.drawModelElements( _panel, _g, _publicOnly, true );    drawModelElements_Parameters_xjal( _panel, _g, _publicOnly, _isSuperClass );    drawModelElements_PlainVariables_xjal( _panel, _g, _publicOnly, _isSuperClass );    drawModelElements_AgentLinks_xjal( _panel, _g, _publicOnly, _isSuperClass );  }  @AnyLogicInternalCodegenAPI  private boolean onClickModelAt_AgentLinks_xjal( Panel _panel, double _x, double _y, int _clickCount, boolean _publicOnly, boolean _isSuperClass ) {    if ( modelElementContains(_x, _y, 50, -100) ) {      if ( _clickCount == 2 ) {        _panel.browseAgent_xjal( 50, -100, this, "main" );      } else {        _panel.addInspect( 50, -100, this, "main" );      }      return true;    }    if ( modelElementContains(_x, _y, 50, -50) ) {        _panel.addInspect_xjal( 50, -50, this, "connections", Panel.INSPECT_CONNECTIONS_xjal );      return true;    }    return false;  }  @AnyLogicInternalCodegenAPI  private boolean onClickModelAt_Parameters_xjal( Panel _panel, double _x, double _y, int _clickCount, boolean _publicOnly, boolean _isSuperClass ) {    if( !_publicOnly && modelElementContains(_x, _y, 50, 50) ) {      _panel.addInspect( 50, 50, this, "id" );       return true;    }    if( !_publicOnly && modelElementContains(_x, _y, 50, 100) ) {      _panel.addInspect( 50, 100, this, "latitude" );       return true;    }    if( !_publicOnly && modelElementContains(_x, _y, 50, 150) ) {      _panel.addInspect( 50, 150, this, "longitude" );       return true;    }    if( !_publicOnly && modelElementContains(_x, _y, 50, 200) ) {      _panel.addInspect( 50, 200, this, "q" );       return true;    }    if( !_publicOnly && modelElementContains(_x, _y, 50, 250) ) {      _panel.addInspect( 50, 250, this, "cost" );       return true;    }    return false;  }  @AnyLogicInternalCodegenAPI  private boolean onClickModelAt_PlainVariables_xjal( Panel _panel, double _x, double _y, int _clickCount, boolean _publicOnly, boolean _isSuperClass ) {    if( !_publicOnly && modelElementContains(_x, _y, 150, 50) ) {      _panel.addInspect( 150, 50, this, "u" );       return true;    }    if( !_publicOnly && modelElementContains(_x, _y, 150, 100) ) {      _panel.addInspect( 150, 100, this, "p" );       return true;    }    if( !_publicOnly && modelElementContains(_x, _y, 150, 150) ) {      _panel.addInspect( 150, 150, this, "vTooBig" );       return true;    }    return false;  }  @Override  @AnyLogicInternalCodegenAPI  public boolean onClickModelAt( Panel _panel, double _x, double _y, int _clickCount, boolean _publicOnly, boolean _isSuperClass ) {    if ( onClickModelAt_AgentLinks_xjal( _panel, _x, _y, _clickCount, _publicOnly, _isSuperClass ) ) { return true; }    if ( onClickModelAt_Parameters_xjal( _panel, _x, _y, _clickCount, _publicOnly, _isSuperClass ) ) { return true; }    if ( onClickModelAt_PlainVariables_xjal( _panel, _x, _y, _clickCount, _publicOnly, _isSuperClass ) ) { return true; }    return super.onClickModelAt( _panel, _x, _y, _clickCount, _publicOnly, true );  }  /**   * Constructor   */  public Hospital( Engine engine, Agent owner, AgentList<? extends Hospital> ownerPopulation ) {    super( engine, owner, ownerPopulation );    instantiateBaseStructureThis_xjal();  }  @AnyLogicInternalCodegenAPI  public void onOwnerChanged_xjal() {    super.onOwnerChanged_xjal();    setupReferences_xjal();  }  @AnyLogicInternalCodegenAPI  public void instantiateBaseStructure_xjal() {    super.instantiateBaseStructure_xjal();    instantiateBaseStructureThis_xjal();  }    @AnyLogicInternalCodegenAPI  private void instantiateBaseStructureThis_xjal() {    setupReferences_xjal();  }    @AnyLogicInternalCodegenAPI  private void setupReferences_xjal() {    main = get_Main();  }    /**   * Simple constructor. Please add created agent to some population by calling goToPopulation() function   */  public Hospital() {  }    /**   * Simple constructor. Please add created agent to some population by calling goToPopulation() function   */  public Hospital( String id, double latitude, double longitude, double q, double cost ) {    markParametersAreSet();    this.id = id;    this.latitude = latitude;    this.longitude = longitude;    this.q = q;    this.cost = cost;  }    @Override  @AnyLogicInternalCodegenAPI  public void doCreate() {    super.doCreate();    // Assigning initial values for plain variables    setupPlainVariables_Hospital_xjal();    // Dynamic initialization of persistent elements    _createPersistentElementsAP0_xjal();    presentation = new ShapeTopLevelPresentationGroup( Hospital.this, true, 0, 0, 0, 0 , rectangle );    icon = new ShapeModelElementsGroup( Hospital.this, getElementProperty( "malawi_recentralization_model_2017_06_04.Hospital.icon", IElementDescriptor.MODEL_ELEMENT_DESCRIPTORS )  );    icon.setIconOffsets( 0.0, 0.0 );	 // Port connectors with non-replicated objects    // Creating replicated embedded objects    setupInitialConditions_xjal( Hospital.class );  }  @AnyLogicInternalCodegenAPI  public void setupExt_xjal(AgentExtension _ext) {    // Agent properties setup    if ( _ext instanceof ExtAgentWithSpatialMetrics && _ext instanceof ExtWithSpaceType ) {      double _value;      _value = 10 ;      ((ExtAgentWithSpatialMetrics) _ext).setSpeed( _value, MPS );    }  }  @Override  @AnyLogicInternalCodegenAPI  public void doStart() {    super.doStart();  }   @AnyLogicInternalCodegenAPI  public void onStartup() {    super.onStartup();    main.hospitalList.add(this);if(this.cost - main.voucher < 0) {	this.vTooBig = true;	main.update_vTooBig();	main.update_costMinusVoucher();}/*switch (this.type) {	case "Central hospital":		this.rectangle.setFillColor(Color.red);		doIExist = true;		this.bType1 = main.centralHosp1;		this.bType2 = main.centralHosp2;		//this.bType2 = main.bCentral2;				break;	case "District hospital":		this.rectangle.setFillColor(Color.blue);		doIExist = true;		this.bType1 = main.districtHosp1;		this.bType2 = main.districtHosp2;		//this.bType2 = main.bDistrict2;		break;	case "Community hospital":		this.rectangle.setFillColor(Color.orange);		doIExist = true;		this.bType1 = main.communityHosp1;		this.bType2 = main.communityHosp2;		//this.bType2 = main.bCommunity2;		break;	case "Other hospital":		this.rectangle.setFillColor(Color.yellow);		doIExist = true;		this.bType1 = main.otherHosp1;		this.bType2 = main.otherHosp2;		//this.bType2 = main.bOther2;		break;	case "Health center":		this.rectangle.setFillColor(Color.lightGray);		if(main.allowAll) {doIExist = true;}		this.bType1 = main.healthCenter1;		this.bType2 = main.healthCenter2;		//this.bType2 = main.bHealthCenter2;		break;	case "Maternity":		this.rectangle.setFillColor(Color.pink);		if(main.allowAll) {doIExist = true;}		this.bType1 = main.maternity1;		this.bType2 = main.maternity2;		//this.bType2 = main.bMaternity2;		break;	case "Clinic":		this.rectangle.setFillColor(Color.green);		if(main.allowAll) {doIExist = true;}		this.bType1 = main.clinic1;		this.bType2 = main.clinic2;		//this.bType2 = main.bClinic2;		break;	default: throw new IllegalArgumentException("Hospital type not found: " + this.type);}*///double b = (this.bobsCounterfactual - 0.151515156) / (1.0 - 0.151515156);//Color c = new Color(round(255f * (float)b), round(255f * (float)b), round(255f * (float)b));//this.rectangle.setFillColor(c);//this.rectangle.setHeight(7);//this.rectangle.setWidth(7);/*if(!main.allowAll){	this.bobs = min(1.0, this.bobsCounterfactual + main.QI);} else {	this.bobs = this.bobsCounterfactual;}if(main.allowFees) {	this.fees = feesCounterfactual;} else {	this.fees = 0;}*///main.update_avgBobs();//main.update_avgFees();   }  /**   * Assigning initial values for plain variables<br>   * <em>This method isn't designed to be called by user and may be removed in future releases.</em>   */  @AnyLogicInternalCodegenAPI  public void setupPlainVariables_xjal() {    setupPlainVariables_Hospital_xjal();  }  /**   * Assigning initial values for plain variables<br>   * <em>This method isn't designed to be called by user and may be removed in future releases.</em>   */  @AnyLogicInternalCodegenAPI  private void setupPlainVariables_Hospital_xjal() {    vTooBig = false ;  }  // User API -----------------------------------------------------  public Main get_Main() {    {      Agent owner = getOwner();      if ( owner instanceof Main ) return (Main) owner;    }    return null;  }  /**   * Read-only variable. <em>Shouldn't be modified by user.</em>   */  @AnyLogicCustomSerialization(AnyLogicCustomSerializationMode.REFERENCE)  public transient malawi_recentralization_model_2017_06_04.Main main;  @AnyLogicInternalCodegenAPI  static LinkToAgentAnimationSettings _connections_commonAnimationSettings_xjal = new LinkToAgentAnimationSettingsImpl( false, black, 1.0, LINE_STYLE_SOLID, ARROW_NONE, 0.0 );  public LinkToAgentCollection<Agent, Agent> connections = new LinkToAgentStandardImpl<Agent, Agent>(this, _connections_commonAnimationSettings_xjal);  @Override  public LinkToAgentCollection<? extends Agent, ? extends Agent> getLinkToAgentStandard_xjal() {    return connections;  }  @AnyLogicInternalCodegenAPI  public void drawLinksToAgents(boolean _underAgents_xjal, LinkToAgentAnimator _animator_xjal) {    super.drawLinksToAgents(_underAgents_xjal, _animator_xjal);    if ( _underAgents_xjal ) {      _animator_xjal.drawLink( this, connections, true, true );    }  }  public AgentList<? extends Hospital> getPopulation() {    return (AgentList<? extends Hospital>) super.getPopulation();  }  public List<? extends Hospital> agentsInRange( double distance ) {    return (List<? extends Hospital>) super.agentsInRange( distance );  }}
